# Periphery Exploration around 2,6-Diazaspiro[3.4]octane Core Identifies a Potent Nitrofuran Antitubercular Lead

**DOI:** 10.3390/molecules28062529

**Published:** 2023-03-10

**Authors:** Alexei Lukin, Kristina Komarova, Lyubov Vinogradova, Marine Dogonadze, Tatiana Vinogradova, Piotr Yablonsky, Alexander Kazantsev, Mikhail Krasavin

**Affiliations:** 1 Lomonosov Institute of Fine Chemical Technologies, MIREA—Russian Technological University, 119454 Moscow, Russia; 2 Saint Petersburg Research Institute of Phthisiopulmonology, 2-4 Ligovsky Prospekt, 191036 Saint Petersburg, Russia; 3 Institute of Chemistry, Saint Petersburg State University, 26 Universitetskii Prospect, 198504 Peterhof, Russia; 4 Institute of Living Systems, Immanuel Kant Baltic Federal University, 236041 Kaliningrad, Russia

**Keywords:** 2,6-diazaspiro[3.4]octane, diversity-oriented synthesis, molecular periphery, azoles, antitubercular activity

## Abstract

A small set of twelve compounds of a nitrofuran carboxamide chemotype was elaborated from a readily available 2,6-diazaspiro[3.4]octane building block, exploring diverse variants of the molecular periphery, including various azole substituents. The in vitro inhibitory activities of the synthesized compounds were assessed against *Mycobacterium tuberculosis* H37Rv. As a result, a remarkably potent antitubercular lead displaying a minimal inhibitory concentration of 0.016 μg/mL was identified.

## 1. Introduction

5-Nitrofuryl-substituted chemotypes have been a prolific source of compounds endowed with antimycobacterial activity due to the ability of the nitrofuran moiety to undergo reduction with the bacterial enzyme machinery and generate reactive intermediates that are lethal to the bacterium itself [1]. The same mode of biotransformation of nitrofurans by mammalian cells is considered the likely origin of the potential toxicity associated with this and other nitroheterocycles [2]. However, the toxicity of nitrofurans is highly dependent on the specific molecular moiety to which these heterocycles are conjugated, and by optimizing the nitrofuran’s molecular periphery, it is possible not only to increase the potency of nitrofuran antibacterials but also alleviate the systemic toxicity of this class of drugs. Indeed, we have previously shown that by linking the 5-nitrofuryl moiety to various heterocyclic motifs, potent antimycobacterial compounds efficacious against multidrug-resistant strains can be developed that are also non-toxic to rodents [3]. Later, it became evident that the conjugation of the 5-nitrofuryl fragment with saturated spirocyclic piperidines was very important for the implementation of this strategy [4]. This motivated us to continue studies along this line of inquiry.

**Figure 1:**
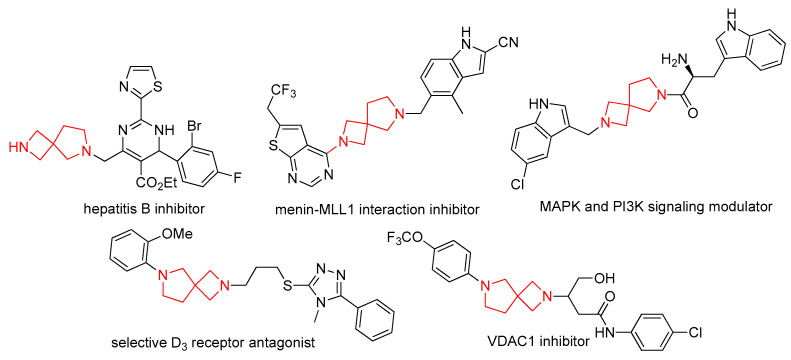
Examples of diverse biologically active compounds containing 2,6-diazaspiro[3.4]octane motif.

The fully saturated, high-F_sp3_ 2,6-diazaspiro[3.4]octane core is an emerging privileged structure, taking into account its recent frequent appearance in compounds possessing diverse biological activities. Notable recently reported examples include a hepatitis B capsid protein inhibitor [5], a menin-MLL1 interaction inhibitor for the treatment of cancer [6], a MAP and PI3K signaling modulator [7], a selective dopamine D_3_ receptor antagonist [8], and VDAC1 inhibitors for the treatment of diabetes [9] (Figure 1).

Considering the significance of the 2,6-diazaspiro[3.4]octane motif in recent drug discovery efforts and the availability of convenient procedures in the literature to access this spirocycle in a functionalized form, we were prompted to synthesize a diverse set of nitrofuran derivatives based on this core that would contain various periphery groups, including a range of azoles. The idea was to demonstrate that by broadly exploring the molecular periphery, potent antitubercular compound(s) could be identified within a relatively small screening set. Herein, we report our findings gathered in the course of realizing this idea.

## 2. Results and Discussion

**Scheme 1:**
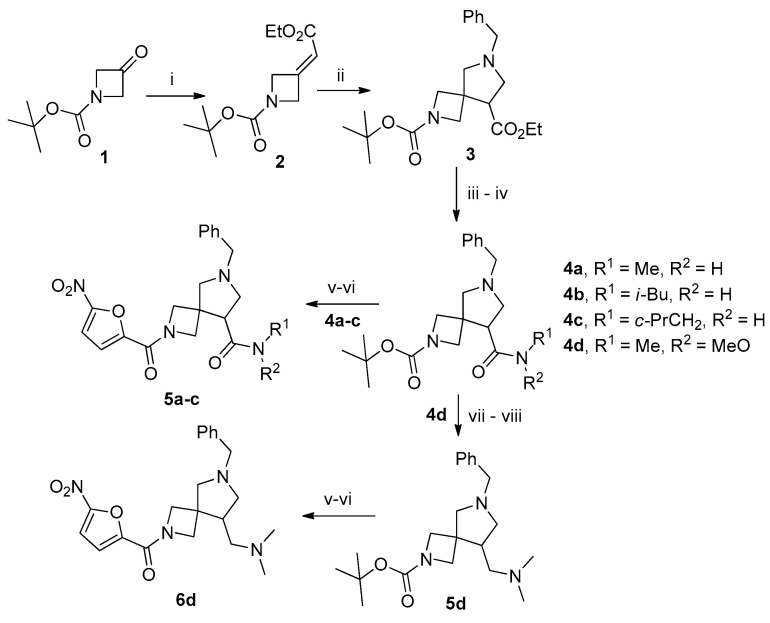
Synthesis of core building block **3** and manipulation of the ester group via amidation. *Reagents and conditions:* i. EtO_2_CCH_2_P(O)(OEt)_2_, NaH, THF, 0 °C→r. t. (92%); ii. MeOCH_2_N(Bn)CH_2_SiMe_3_, LiF, MeCN, 60 °C, 18 h (56%); iii. LiOH∙H2O, aq. 1,4-dioxane, r. t., 12 h; iv. RNHR′, HOBt, EDC∙HCl, r. t., 12 h (**4a** 66%, **4b** 68%, **4c** 72%, **4d** 72%, 2 steps); v. TFA, CH_2_Cl_2_, 0 °C→r. t., 1 h; vi. 5-nitro-2-furoic acid, CDI, DMF, r. t., 1 h (**5a** 56%, **5b** 56%, **5c** 48%, **6d** 38%, 2 steps); vii. LiAlH_4_, THF, −70 °C→−5 °C; viii. NaBH(OAc)_3_, CH_2_Cl_2_, r. t., 18 h (72%, 2 steps).

The synthesis of core building block **3** on a multigram scale was achieved from commercially available *N*-Boc-protected azetidine-3-one **1**, as described in the literature [10]. The initial Horner-Wadsworth-Emmons olefination proceeded in high yield and gave α,β-unsaturated ester **2** following chromatographic purification. A [3 + 2] cycloaddition with **2** and *N*-(methoxymethyl)-*N*-(trimethylsilylmethyl) benzylamine in the presence of lithium fluoride afforded intermediate core building block **3** at 56% yield, which was sufficiently pure without chromatography. The ester functionality of **3** was manipulated first by one-pot alkaline hydrolysis and then transformation to amides **4a**–**d**. Amides **4a**–**c** were transformed into 5-nitrofuroyl-substituted compounds **5a**–**c** via a two-step deprotection-acylation protocol. Weinreb amide **4d** was reduced to the respective aldehyde, which was subjected to reductive amination with dimethylamine to give compound **5d** at 72% yield over two steps. Finally, compound **5d** was transformed into 5-nitrofuroyl derivative **6d** analogously to amides **4a**–**c** (Scheme 1).

**Scheme 2:**
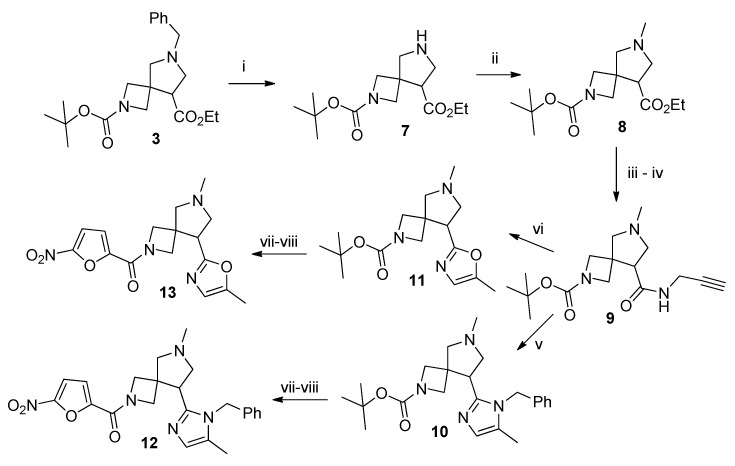
Synthesis of imidazole (**12**) and oxazole (**13**) derivatives. *Reagents and conditions:* i. H_2_, 10% Pd-C, 100 atm, r. t., 12 h (quant.); ii. 37% aq. formaldehyde, CH_2_Cl_2_, NaBH(OAc)_3_, r. t., 18 h (72%); iii. LiOH∙H_2_O, aq. 1,4-dioxane, r. t., 12 h; iv. propargylamine, HOBt, EDC∙HCl, r. t., 12 h (66%, 2 steps); v. BnNH_2_, Zn(OTf)_2_ (0.25 mol.%), toluene, reflux with Dean-Stark trap, 8 h (75%); vi. Zn(OTf)_2_ (0.25 mol.%), toluene, reflux with Dean-Stark trap, 16 h (47%); vii. TFA, CH_2_Cl_2_, 0 °C→r. t., 1 h; viii. 5-nitro-2-furoic acid, CDI, DMF, r. t., 1 h (28%, 2 steps).

Next, we aimed to elaborate the ester functionality in **3** into an imidazole and an oxazole moiety. In order to reduce the lipophilicity of the scaffold, we removed the benzyl group by hydrogenation (to obtain **7**) and replaced it with a methyl group using a reductive alkylation procedure. After ester hydrolysis in intermediate **8**, we prepared propargylamide **9**, which was viewed as a starting material for both imidazole and oxazole. Following the protocol from the Beller group [11], propargylamide **9**, without purification, underwent Zn(OTf)_2_-catalyzed hydroamination with benzylamine, which led to cyclodehydrative aromatization and the formation of imidazole **10** at 75% yield. In the absence of benzylamine (or any other primary amine), cycloaromatization under the same conditions led to the formation of oxazole **11** [12]. Compounds **10** and **11** were transformed into their *N*-(5-nitrofuroyl) derivatives using the same one-pot deprotection-acylation protocol as described above to furnish nitrofurans **12** and **13**, respectively (Scheme 2).

**Scheme 3:**
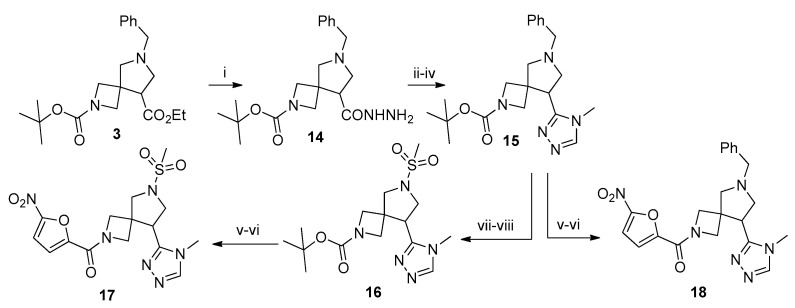
Synthesis of 4-methyl-1,2,4-triazol-3-yl derivatives. *Reagents and conditions:* i. 64% aq. N_2_H_4_, ethanol, reflux, 8 h (quant.); ii. MeNCS, ethanol, reflux, 2 h; iii. sat. aq. K_2_CO_3_, reflux, 8 h iv. Raney Ni, ethanol, reflux, 12 h (58%, 3 steps); v. TFA, CH_2_Cl_2_, 0 °C→r. t., 1 h; vi. 5-nitro-2-furoic acid, CDI, DMF, r. t., 1 h (**17** 56%, **18** 62%, 2 steps); vii. H_2_, 10% Pd-C, 100 atm, r. t., 12 h; viii. MsCl, Et_3_N, CH_2_Cl_2_, 0 °C→r. t., 18 h (53%, 2 steps).

Next, we turned our attention to elaborating the ester functionality in **3** into the 4-methyl-1,2,4-triazole group while also varying the substituent on the pyrrolidine nitrogen atom. Compound **3** was converted to hydrazide **14** by refluxing with hydrazine in ethanol and used without purification. Hydrazide **14** was reacted with methyl isothiocyanate and the intermediate hydrazine carbothioamide was treated with base and the cyclized intermediate with Raney nickel, which triggered the conversion into 1,2,4-triazole **15**. On the one hand, the latter was converted into 5-nitrofuroyl derivative **18**, and on the other hand, **15** was subjected to benzyl-to-mesyl group swap on the nitrogen atom to give *N*-mesyl derivative **16**, which was converted into 5-nitrofuroyl compound **17** (Scheme 3).

**Scheme 4:**
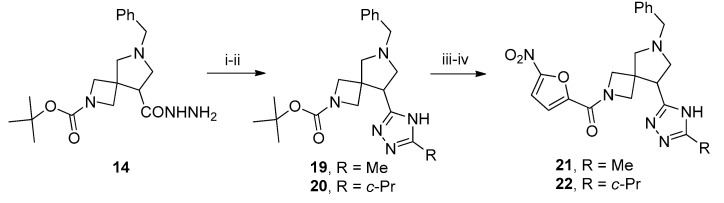
Synthesis of 5-substituted 1,2,4-triazol-3-yl derivatives. *Reagents and conditions:* i. acetamidine (cyclopropane carboxamidine) HCl, MeONa, MeOH, r. t., 18 h; ii 170 °C in argon stream (**19** 48%, **20** 42%, 2 steps); iii. TFA, CH_2_Cl_2_, 0 °C→r. t., 1 h; iv. 5-nitro-2-furoic acid, CDI, DMF, r. t., 1 h (**21** 43%, **22** 38%, 2 steps).

Further, we were interested in replacing the ester functionality in **3** with a differently substituted 1,2,4-triazole moiety. To this end, hydrazide **14** was reacted with two amidines (acetamidine and cyclopropane carboxamidine). The initial adduct was cyclized on heating to 170 °C to give 1,2,4-triazoles **19** and **20**, respectively. Each of the latter was converted into a 5-nitrofuroyl derivative (**21** and **22**, respectively) using the already established one-pot, two-step protocol (Scheme 4).

**Scheme 5:**
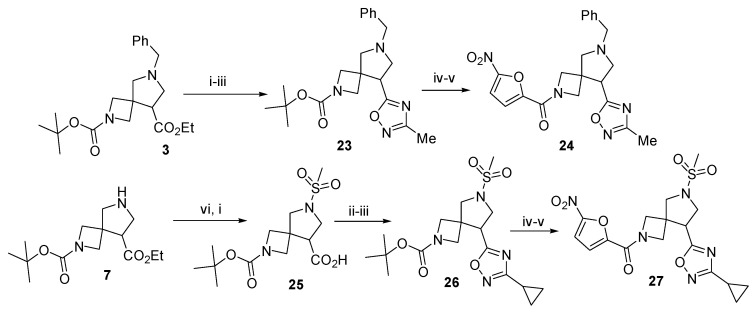
Synthesis of 1,2,4-oxadiazole derivatives. *Reagents and conditions:* i. LiOH∙H_2_O, aq. 1,4-dioxane, r. t., 12 h; ii. acetamidoxime, HOBt, EDC∙HCl, r. t., 12 h; iii. TBAF, toluene, Dean-Stark trap, reflux, 6 h (**23** 41%, **26** 63%, 3 steps); iv. TFA, CH_2_Cl_2_, 0 °C→r. t., 1 h; v. 5-nitro-2-furoic acid, CDI, DMF, r. t., 1 h (**24** 48%, **27** 63%, 2 steps); vi. MsCl, Et_3_N, CH_2_Cl_2_, 0 °C→r. t., 18 h (quant.).

Finally, we were interested in grafting a 1,2,4-oxadiazole moiety in lieu of the ester group on the 2,6-diazaspiro[3.4]octane building block **3** while also varying the substituent on the pyrrolidine nitrogen atom. To this end, we hydrolyzed the ester functionality in **3**, acylated acetamidoxime with the resulting acid, and subjected the resulting intermediate to TBAF-promoted cyclodehydration. The resulting 1,2,4-oxadiazole **23** was transformed into 5-nitrofuroyl derivative **24**. *N*-Unsubstituted ester **7** from our previous synthesis depicted in Scheme 2 was *N*-mesylated and subjected to ester hydrolysis to furnish carboxylic acid **25** in quantitative yield over two steps. The latter was transformed to 3-cyclopropyl-1,2,4-oxadiazol-5-yl derivative **26** using the same sequence of steps as was applied towards **23**. *N*-Boc protected compound **26** was transformed into 5-nitrofuroyl compound **27** (Scheme 5).

Having thus synthesized twelve 5-nitrofuroyl derivatives (**5a**–**c**, **6d**, **12**–**13**, **17**–**18**, **21**–**22**, **24**, and **27**), we proceeded to evaluate their activity against the drug-sensitive strain of *Mycobacterium tuberculosis* H37Rv using the resazurin microtiter plate assay (REMA) [13]. The resulting data expressed as minimum inhibitory concentrations (MIC) are collated in Table 1.

As follows from the data summarized in Table 1, most of the compounds were either weakly active or inactive against this strain of *M. tuberculosis*. However, two specific compounds (out of only twelve)—1,2,4-oxadiazole **24** and especially 1,2,4-triazole **17**—displayed quite respectable levels of activity. In fact, compound **17** was very active and caused the bacteria to die at a minimum inhibitory concentration of 0.016 μg/mL.

Comparing the activity of compounds **17** and **18**, it would be assumed that the N-mesyl group strongly promoted activity. On the other hand, when comparing compounds **18** and **24**, one can note an increase in activity when replacing 1,2,4- triazole with 1,2,4-oxadiazole. Based on these two comparisons, one should expect outstanding results from compound **27**, but this was not reflected in the data in Table 1. Therefore, there was no unambiguous correlation between structure and property, and further research is required.

## 3. Material and Methods

### 3.1. Synthesis—General

All commercial reagents were used without purification. NMR spectra were recorded using a Bruker DPX-300 spectrometer in CDCl_3_ (^1^H: 300 MHz; ^13^C: 75 MHz). Chemical shifts are reported as parts per million (δ, ppm). The residual solvent peak (CHCl_3_ or DMSO-*d*_6_) was used as the internal standard: 7.28 or 2.51 for ^1^H and 77.07 or 40.00 ppm for ^13^C. Multiplicities are abbreviated as follows: s = singlet, d = doublet, t = triplet, q = quartet, m = multiplet, br = broad, dd = doublet of doublets, dt = doublet of triplets, ddd = doublet/doublets of doublets (see Supplementary Materials). Coupling constants, J, are reported in Hz. Mass spectra were recorded using a Bruker microTOF spectrometer (ionization by electrospray, positive ion detection). Melting points were determined in open capillary tubes using a Stuart SMP50 Automatic Melting Point Apparatus. Analytical thin-layer chromatography was carried out on UV-254 silica gel plates using appropriate eluents. Compounds were visualized with short-wavelength UV light. Column chromatography was performed using silica gel Merk grade 60 (0.040–0.063 mm) 230–400 mesh. All reactions were conducted under an atmosphere of argon.

### 3.2. 2-(tert-Butyl) 8-ethyl 6-benzyl-2,6-diazaspiro[3.4]octane-2,8-dicarboxylate (***3***)

Prepared according to the literature procedure [10] with minor modifications. To a suspension of NaH (60% dispersion in mineral oil, 1.88 g, 0.047 mol, 1.15 equiv.) in THF (150 mL) at 0 °C, triethylphosphonoacetate (11 g, 0.054 mol, 1.2 equiv.) was added. The resulting mixture was allowed to warm up to r. t. and stirred at that temperature for 30 min. It was cooled down to 0 °C, at which point *tert*-butyl 3-oxazetidine-1-carboxylate (7 g, 0.041 mol, 1.0 equiv.) in THF (50 mL) was added. The reaction mixture was allowed to reach r. t. again and stirred at that temperature overnight. It was then diluted with ethyl acetate and washed with sat. aq. NaHCO_3_, water, and brine. The organic phase was separated and dried over anhydrous Na_2_SO_4_, filtered and concentrated in vacuo. The residue was purified by column chromatography on silica, eluting with 0→10% ethyl acetate in hexane. Yield of **2**—9 g (92%), colorless oil. The spectral data of compound **2** were in accordance with those reported in the literature: ^1^H NMR (300 MHz, CDCl_3_) δ 5.74 (dd, *J* = 4.5, 2.2 Hz, 1H), 4.80 (dd, *J* = 6.3, 2.9 Hz, 2H), 4.57 (dt, *J* = 5.3, 2.7 Hz, 2H), 4.31–4.02 (m, 2H), 1.45 (s, 9H), 1.26 (t, *J* = 7.1 Hz, 3H); LCMS (ESI): *m/z* (M + H) calcd, 242.3; found, 242.2.

To a solution of **2** (5 g, 20.75 mmol, 1 equiv.) in acetonitrile (50 mL), LiF (2.15 g, 83 mmol, 4 equiv.) and (methoxymethyl)-1-phenyl-*N*-(trimethylsilylmethyl)methanamine (6.25 g, 25 mmol, 1.2 equiv.) were added and the resulting mixture was stirred at 60 °C overnight. The solvent was removed in vacuo and the residue was dissolved in ethyl acetate (50 mL). The solution was washed with sat. aq. citric acid (3 × 25 mL). The combined aqueous phases were extracted with ethyl acetate (2 × 100 mL). The pH of the aqueous phase was brought to 8 with sat. aq. K_2_CO_3_ and it was extracted with ethyl acetate (2 × 100 mL). The combined organic phases were dried over anhydrous Na_2_SO_4_, filtered, and concentrated in vacuo to give 4.3 g (56%) of the title product as a colorless oil. This product was used in subsequent steps without further purification. The spectral data of compound **3** were in accordance with those reported in the literature: ^1^H NMR (300 MHz, DMSO-*d*_6_) δ 7.46–7.14 (m, 5H), 4.22–4.00 (m, 2H), 3.94 (dd, *J* = 16.6, 8.4 Hz, 2H), 3.78–3.51 (m, 5H), 3.12 (t, *J* = 7.6 Hz, 1H), 2.83–2.66 (m, 4H), 1.36 (s, 9H), 1.19 (t, *J* = 7.1 Hz, 3H); LCMS (ESI): *m/z* (M + H^+^) calcd, 375.5; found, 375.4.

### 3.3. 2-tert-Butoxycarbonyl-6-benzyl-8-(methylcarbamoyl)-2,6-diazaspiro[3.4]octane (***4a***)

General Procedure A. To a solution of **3** (1 g, 3.35 mmol, 1.0 equiv.) in 1,4-dioxane (10 mL) was added aqueous solution (1 mL) of LiOH∙H_2_O (0.175 g, 4.2 mmol, 1.25 equiv.) and the resulting mixture was stirred for 12 h. HOBt (0.56 g, 4.2 mmol,1.25 equiv.), EDC∙HCl (0.81 g, 4.2 mmol, 1.25 equiv.), and methylamine (1.4 equiv.) were added and the resulting mixture was stirred for 12 h. The solvent was removed in vacuo and the residue was dissolved in ethyl acetate (50 mL). The solution was washed with 10% aqueous K_2_CO_3_, brine, dried over anhydrous Na_2_SO_4,_, filtered, and concentrated in vacuo to obtain 0.68 g (66%) of the title compound as a colorless oil. ^1^H NMR (300 MHz, CDCl_3_) δ 7.38–7.19 (m, 5H), 7.00 (s, 1H), 3.88 (d, *J* = 4.4 Hz, 1H), 3.85 (d, *J* = 5.0 Hz, 1H), 3.79 (d, *J* = 8.4 Hz, 1H), 3.69 (d, *J* = 9.1 Hz, 1H), 3.63 (s, 1H), 3.01 (d, *J* = 9.7 Hz, 1H), 2.89–2.62 (m, 6H), 1.39 (s, 9H); ^13^C NMR (75 MHz, CDCl3) δ 173.52, 156.08, 138.07, 128.53, 128.45, 127.33, 79.56, 65.33, 59.44, 57.16, 53.85, 42.07, 28.28, 25.99; HRMS (ESI) *m/z* calcd for C_20_H_30_N_3_O_3_ [M + H^+^] 360.2287, found 360.2291.

### 3.4. 2-tert-Butoxycarbonyl-6-benzyl-8-(isobutylcarbamoyl)-2,6-diazaspiro[3.4]octane (***4b***)

This compound was synthesized according to general procedure A from **3** (0.5 g, 1.33 mmol). Yield 0.36 g (68%), colorless oil. ^1^H NMR (300 MHz, CDCl_3_) δ 7.40–7.10 (m, 6H), 3.90 (d, *J* = 8.9 Hz, 2H), 3.80 (d, *J* = 8.5 Hz, 1H), 3.69 (d, *J* = 9.1 Hz, 1H), 3.63 (s, 2H), 3.15–2.98 (m, 3H), 2.92 (dd, *J* = 9.6, 2.7 Hz, 1H), 2.80 (dd, *J* = 6.5, 2.7 Hz, 1H), 2.73–2.52 (m, 2H), 1.82–1.60 (m, 1H), 1.39 (s, 9H), 0.87 (dd, *J* = 6.7, 1.0 Hz, 6H); ^13^C NMR (75 MHz, CDCl_3_) δ 178.85, 167.17, 155.96, 138.18, 128.53, 128.37, 127.24, 79.69, 64.33, 59.41, 57.47, 43.93, 43.22, 29.65, 28.26, 11.60, −0.05; HRMS (ESI) *m/z* calcd for C_23_H_36_N_3_O_3_ [M + H^+^] 402.2756, found 402.2761.

### 3.5. 2-tert-Butoxycarbonyl-6-benzyl-N-(cyclopropylmethyl)-2,6-diazaspiro[3.4]octane-8-carboxamide (***4c***)

This compound was synthesized according to general procedure A from **3** (0.5 g, 1.33 mmol). Yield 0.38 g (72%), colorless oil. ^1^H NMR (300 MHz, CDCl_3_) δ 7.40–7.14 (m, 5H), 3.91 (d, *J* = 9.2 Hz, 2H), 3.81 (d, *J* = 8.5 Hz, 1H), 3.70 (d, *J* = 9.0 Hz, 1H), 3.64 (d, *J* = 1.8 Hz, 1H), 3.14–3.02 (m, 2H), 2.92 (dd, *J* = 9.5, 2.8 Hz, 1H), 2.80 (dd, *J* = 6.6, 2.9 Hz, 1H), 2.72–2.57 (m, 2H), 1.03–0.77 (m, 1H), 0.57–0.35 (m, 2H), 0.25–0.08 (m, 2H); ^13^C NMR (75 MHz, CDCl_3_) δ 172.72, 138.13, 128.48, 128.44, 127.35, 79.52, 65.67, 59.49, 57.17, 54.05, 43.98, 41.98, 28.27, 10.71, 3.39; HRMS (ESI) *m*/*z* calcd for C_23_H_34_N_3_O_3_ [M + H^+^] 400.2600, found 400.2597.

### 3.6. 2-tert-Butoxycarbonyl-1-(6-benzyl-2,6-diazaspiro[3.4]oct-8-yl)-N,N-dimethylmethanamine (***5d***)

Compound **4d** was synthesized according to general procedure A from **3** (1 g, 2.66 mmol) and used in the next step without further purification. A solution of this compound (0.7 g, 1.8 mmol, 1 equiv.) in absolute THF (3 mL) was added to a suspension of LAH (0.07 g, 1.8 mmol, 1 equiv.) in THF (10 mL) at −70 °C. The reaction mixture was stirred at that temperature for 30 min and allowed to reach −5 °C. Then, it was cooled to −30 °C and decomposed by adding water (0.1 mL), 15% aq. NaOH (0.1 mL), and water (0.3 mL). The resulting precipitate was filtered off and the filtrate was concentrated in vacuo. The resulting aldehyde was used immediately in the next step. It was dissolved in CH_2_Cl_2_ (10 mL) and the solution was treated, on vigorous stirring, with 33% aq. dimethylamine (0.5 mL). Sodium triacetoxyborohydride (0.96 g, 4.5 mmol, 2.5 equiv.) was added in portions and the resulting mixture was stirred overnight. Sat. aq. NaHCO_3_ was added and the resulting mixture was washed with 10% aq. K_2_CO_3_, brine, dried over anhydrous Na_2_SO_4_, filtered, and concentrated in vacuo. The residue was purified by column chromatography on silica, eluting with 10% methanol in CH_2_Cl_2_ to give 0.25 g (72%) of the title compound as a colorless oil. ^1^H NMR (300 MHz, CDCl_3_) δ 7.47–7.06 (m, 5H), 4.07 (d, *J* = 8.8 Hz, 1H), 3.78 (s, 2H), 3.66–3.48 (m, 3H), 3.01–2.91 (m, 1H), 2.89 (d, *J* = 9.3 Hz, 1H), 2.55 (d, *J* = 9.3 Hz, 1H), 2.45–2.30 (m, 1H), 2.30–2.15 (m, 9H), 1.42 (s, 9H); ^13^C NMR (75 MHz, CDCl_3_) δ 156.38, 138.61, 128.65, 128.22, 126.97, 79.28, 65.56, 61.35, 60.07, 59.18, 45.67, 41.99, 41.73, 28.33; HRMS (ESI) *m/z* calcd for C_21_H_34_N_3_O_2_ [M + H^+^] 360.2651, found 360.2655.

### 3.7. 2-tert-Butoxycarbonyl-8-(1-benzyl-5-methyl-1H-imidazol-2-yl)-6-methyl-2,6-diazaspiro[3.4]octane (***10***)

To a solution of **3** (2 g, 5.3 mmol) in ethanol (25 mL) was added 10% Pd/C (0.25 g) and the mixture was hydrogenated in an autoclave at a start pressure of 100 atm and r. t. for 12 h. The reaction mixture was filtered through a plug of Celite and the filtrate was concentrated in vacuo. The yield of compound **7** was 1.6 g (quantitative, assuming analytical purity), obtained as a colorless oil. It was used directly in the next step without further purification.

To a vigorously stirred solution of **7** (1.6 g, 5.3 mmol, 1 equiv.) in CH_2_Cl_2_ (25 mL), 37% aq. formaldehyde solution (1 mL) was added followed by portion-wise addition of sodium triacetoxyborohydride (2.8 g, 13.25 mmol, 2.5 equiv.). The resulting mixture was stirred overnight. Sat. aq. NaHCO_3_ (10 mL) was added, whereupon the mixture was washed with 10% aq. K_2_CO_3_, brine, dried over anhydrous Na_2_SO_4_, filtered, and concentrated in vacuo. The resulting product **8** (1.2 g, 72%, assuming analytical purity) was used in the next step without further purification.

To a solution of **8** (1 g, 3.35 mmol, 1.0 equiv.) in 1,4-dioxane (10 mL), aq. solution of LiOH∙H_2_O (1 mL, 0.175 g, 4.2 mmol, 1.25 equiv.) was added and the resulting mixture was stirred for 12 h. Thereupon HOBt (0.56 g, 4.2 mmol, 1.25 equiv.), EDC∙HCl (0.81 g, 4.2 mmol, 1.25 equiv.), and propargylamine (0.29 g, 4.7 mmol, 1.4 equiv.) were added. The resulting mixture was stirred for 12 h. The solvent was removed in vacuo and the residue was dissolved in ethyl acetate (50 mL). The solution was washed with 10% aq. K_2_CO_3_, brine, dried over anhydrous Na_2_SO_4_, filtered, and concentrated in vacuo to give 0.68 g (66%, assuming analytical purity) of product **9** as a colorless oil, which was used in the next step without further purification.

To a solution of compound **9** (0.3 g, 0.97 mmol, 1 equiv.) in toluene (10 mL), benzylamine (0.13 g, 0.24 mmol, 1.25 equiv.) was added followed by Zn(OTf)_2_ (0.088 g, 1.21 mmol, 0.25 equiv.). The mixture was heated under reflux with a Dean-Stark trap over 8 h. Upon cooling to r. t., the reaction mixture was washed with 10% aq. K_2_CO_3_, brine, dried over anhydrous Na_2_SO_4_, filtered, and concentrated in vacuo. The residue was purified by column chromatography on silica gel using 10% methanol in CH_2_Cl_2_ to give the title compound (0.29 g, 75%) as a colorless oil. ^1^H NMR (300 MHz, CDCl_3_) δ 7.35–7.19 (m, 3H), 6.96–6.86 (m, 2H), 6.83 (s, 1H), 5.19–4.93 (m, 2H), 3.77 (d, *J* = 8.6 Hz, 1H), 3.60 (d, *J* = 9.5 Hz, 1H), 3.47–3.29 (m, 3H), 3.11 (d, *J* = 9.3 Hz, 1H), 3.05 (t, *J* = 8.6 Hz, 1H), 2.33 (s, 3H), 2.15 (d, *J* = 0.7 Hz, 3H), 1.39 (s, 9H); ^13^C NMR (75 MHz, CDCl_3_) δ 155.97, 147.40, 136.58, 129.02, 127.85, 127.40, 125.84, 125.47, 79.24, 68.07, 62.57, 46.39, 44.59, 44.36, 41.55, 28.31, 9.70; HRMS (ESI) *m/z* calcd for C_23_H_33_N_4_O_2_ [M + H^+^] 397.2603, found 397.2598.

### 3.8. 2-tert-Butoxycarbonyl-6-methyl-8-(5-methyloxazol-2-yl)-2,6-diazaspiro[3.4]octane (***11***)

To a solution of compound **9** (0.3 g, 0.97 mmol, 1 equiv.) in toluene (10 mL), Zn(OTf)_2_ (0.088 g, 1.21 mmol, 0.25 equiv.) was added and the mixture was heated under reflux with a Dean-Stark trap over 16 h. Upon cooling to r. t., the mixture was washed with 10% aq. K_2_CO_3_, brine, dried over anhydrous Na_2_SO_4_, filtered, and concentrated in vacuo. The residue was purified by column chromatography on silica gel using 10% methanol in CH_2_Cl_2_ to give the title compound (0.14 g, 47%) as a colorless oil. ^1^H NMR (300 MHz, CDCl_3_) δ 6.63 (d, *J* = 1.2 Hz, 1H), 4.00 (d, *J* = 8.6 Hz, 1H), 3.89 (d, *J* = 8.6 Hz, 1H), 3.60 (d, *J* = 9.2 Hz, 1H), 3.51 (t, *J* = 8.0 Hz, 1H), 3.41 (d, *J* = 9.2 Hz, 1H), 3.18–3.10 (m, 1H), 3.06 (d, *J* = 9.4 Hz, 1H), 2.82–2.70 (m, 2H), 2.39 (s, 3H), 2.27 (s, 3H), 1.40 (s, 9H); ^13^C NMR (75 MHz, CDCl3) δ 162.33, 156.09, 148.93, 122.69, 79.38, 67.35, 59.58, 46.23, 43.93, 41.86, 28.26, 10.79; HRMS (ESI) *m/z* calcd for C_16_H_26_N_3_O_3_ [M + H^+^] 308.1974, found 308.1980.

### 3.9. 2-tert-Butyl 6-benzyl-8-(4-methyl-4H-1,2,4-triazol-3-yl)-2,6-diazaspiro[3.4]octane-2-carboxylate (***15***)

To a solution of **3** (3 g, 8 mmol) in ethanol (25 mL), N_2_H_4_ (64% aq. solution, 1 mL) was added. The reaction mixture was heated under reflux for 8 h. Upon cooling to r. t., the mixture was concentrated in vacuo to give compound **14** (2.8 g, quant., assuming analytical purity), which was used in the next step without further purification.

To a solution of **14** (2 g, 5.5 mmol, 1 equiv.) in ethanol (25 mL), CH_3_NCS (0.5 g, 6.8 mmol, 1.25 equiv.) was added dropwise and the resulting mixture was heated under reflux for 2 h. Sat. aq. K_2_CO_3_ (5 mL) was added and refluxing continued for 8 h. The reaction mixture was concentrated in vacuo, the residue was dissolved in water (25 mL), and the solution was acidified to pH 7 with 5% aq. HCl. The resulting precipitate was filtered off and dissolved in ethanol (25 mL). A suspension of freshly prepared Raney nickel in a minimum amount of ethanol was added and the mixture was heated under reflux with vigorous stirring for 12 h. Upon cooling to r. t., the reaction mixture was filtered through a plug of Celite and the filtrate was concentrated in vacuo. The residue was purified by column chromatography on silica gel using 10% methanol in CH_2_Cl_2_ to give the title compound (1.22 g, 58%) as a colorless oil. ^1^H NMR (300 MHz, CDCl_3_) δ 8.08 (s, 1H), 7.39–7.13 (m, 5H), 4.01–3.84 (m, 2H), 3.74–3.59 (m, 6H), 3.53 (t, *J* = 8.0 Hz, 1H), 3.35 (d, *J* = 9.5 Hz, 1H), 3.28 (t, *J* = 8.8 Hz, 1H), 3.12 (d, *J* = 9.4 Hz, 1H), 2.97–2.86 (m, 1H), 2.80 (d, *J* = 9.4 Hz, 1H), 1.37 (s, 9H); ^13^C NMR (75 MHz, CDCl_3_) δ 156.25, 154.24, 144.60, 138.35, 128.65, 128.34, 127.19, 79.74, 64.86, 59.57, 58.88, 43.59, 42.08, 30.78, 28.23; HRMS (ESI) *m/z* calcd for C_21_H_30_N_5_O_2_ [M + H^+^] 384.2399, found 384.2405.

### 3.10. 2-tert-Butoxycarbonyl-6-(methylsulfonyl)-8-(4-methyl-4H-1,2,4-triazol-3-yl)-2,6-diazaspiro[3.4]octane (***16***)

Compound **15** was hydrogenated as described for the preparation of compound **7** to give 0.76 g of a colorless oil. To a solution of this oil (0.35 g, 1.1 mmol, 1.0 equiv.) in CH_2_Cl_2_ (10 mL), Et_3_N (0.14 g, 1.37 mmol, 1.25 equiv.) was added dropwise. The mixture was cooled to 0 °C and treated with MsCl (0.16 g, 1.37 mmol, 1.25 equiv.). After stirring the mixture overnight, it was washed with 10% aq. K_2_CO_3_, brine, dried over anhydrous Na_2_SO_4_, filtered, and concentrated in vacuo. The residue was purified by column chromatography on silica gel using 10% methanol in CH_2_Cl_2_ to give the title compound (0.22 g, 53%) as a colorless oil. ^1^H NMR (300 MHz, CDCl_3_) δ 8.11 (s, 1H), 3.04–3.95 (m, 2H), 3.91–3.75 (m, 4H), 3.64–3.52 (m, 2H), 1.40 (s, 9H).; ^13^C NMR (75 MHz, CDCl_3_) δ 156.03, 152.70, 144.60, 80.13, 61.23, 55.73, 53.35, 51.38, 43.74, 41.23, 35.65, 30.95, 28.20; HRMS (ESI) *m/z* calcd for C_15_H_26_N_5_O_4_S [M + H^+^] 372.1705, found 372.1701.

### 3.11. 2-tert-Butoxycarbonyl-6-benzyl-8-(5-methyl-4H-1,2,4-triazol-3-yl)-2,6-diazaspiro[3.4]octane (***19***)

To a solution of acetamidine hydrochloride (0.16 g, 1.73 mmol, 1.25 equiv.) in absolute methanol (10 mL), MeONa (0.093 g, 1.73 mmol, 1.25 equiv.) was added. The mixture was stirred for 30 min, whereupon crude **14** (0.5 g, 1.39 mmol, 1.0 equiv.) was added and the stirring continued overnight. The solvent was removed in vacuo and the residue was heated at 170 °C under an argon stream for 15 min. The residue was dissolved in ethyl acetate (25 mL), washed with 10% aq. K_2_CO_3_, brine, dried over anhydrous Na_2_SO_4_, filtered, and concentrated in vacuo. The residue was purified by column chromatography on silica gel using 10% methanol in CH_2_Cl_2_ to give the title compound (0.26 g, 48%) as a colorless oil. ^1^H NMR (300 MHz, CDCl_3_) δ 7.39–7.13 (m, 5H), 3.92 (dd, *J* = 37.4, 8.6 Hz, 2H), 3.70 (q, *J* = 12.8 Hz, 2H), 3.59–3.41 (m, 3H), 3.20–2.99 (m, 2H), 2.91 (t, *J* = 8.0 Hz, 2H), 2.38 (s, 3H), 1.37 (m, 9H); ^13^C NMR (75 MHz, CDCl_3_) δ 156.29, 138.23, 128.69, 128.42, 127.27, 79.68, 64.71, 59.87, 53.40, 45.16, 43.19, 28.30, 8.08, −0.05; HRMS (ESI) *m/z* calcd for C_21_H_30_N_5_O_2_ [M + H^+^] 384.2399, found 384.2404.

### 3.12. 2-tert-Butoxycarbonyl-6-benzyl-8-(3-cyclopropyl-1H-1,2,4-triazol-5-yl)-2,6-diazaspiro[3.4]octane (***20***)

Synthesized analogously to **19**. Yield 0.23 g (42%), colorless oil. ^1^H NMR 1H NMR (300 MHz, CDCl3) δ 7.39–7.16 (m, 5H), 3.91 (dd, *J* = 41.5, 8.6 Hz, 2H), 3.69 (q, *J* = 12.8 Hz, 2H), 3.57–3.41 (m, 3H), 2.04–1.86 (m, 1H), 1.38 (s, 9H), 1.00–0.94 (m, 4H).; ^13^C NMR (75 MHz, DMSO-*d*_6_) δ 156.29, 138.23, 128.69, 128.42, 127.27, 79.68, 64.71, 59.87, 53.40, 45.16, 43.19, 28.30, 8.08, −0.05; HRMS (ESI) *m/z* calcd for C_23_H_32_N_5_O_2_ [M + H^+^] 410.2556, found 410.2561.

### 3.13. 2-tert-Butoxycarbonyl-6-benzyl-8-(3-methyl-1,2,4-oxadiazol-5-yl)-2,6-diazaspiro[3.4]octane (***23***)

To a solution of **3** (1 g, 2.29 mmol, 1 equiv.) in 1,4-dioxane, an aqueous solution of LiOH∙H_2_O (1 mL, 0.175 g, 4.2 mmol,1.25 equiv.) was added and the mixture was stirred for 12 h. HOBt (0.56 g, 4.2 mmol, 1.25 equiv.), EDC∙HCl (0.81 g, 4.2 mmol, 1.25 equiv.), and acetamidoxime (0.35 g, 4.7 mmol, 1.4 equiv.) were added and the resulting mixture was stirred for 12 h. The solvent was removed in vacuo and the residue was dissolved in ethyl acetate (50 mL). The solution was washed with 10% aq. K_2_CO_3_, brine, dried over anhydrous Na_2_SO_4_, filtered, and concentrated in vacuo. The residue was dissolved in toluene (25 mL), TBAF (100 mg) was added, and the resulting mixture was heated under reflux with a Dean-Stark trap for 6 h. The reaction mixture was concentrated in vacuo. The residue was purified by column chromatography on silica gel using 10% methanol in CH_2_Cl_2_ to give the title compound (0.48 g, 41%) as a colorless oil. ^1^H NMR (300 MHz, CDCl_3_) δ 7.41–7.17 (m, 5H), 3.94 (dd, *J* = 33.0, 8.6 Hz, 2H), 3.78–3.59 (m, 4H), 3.49 (d, *J* = 9.3 Hz, 1H), 3.33–3.21 (m, 1H), 3.09 (d, *J* = 9.4 Hz, 1H), 2.90–2.72 (m, 2H), 2.40 (s, 3H), 1.39 (s, 9H).; ^13^C NMR (75 MHz, DMSO-*d*_6_) δ 178.85, 167.17, 155.96, 138.18, 128.53, 128.37, 127.24, 79.69, 59.41, 57.47, 43.93, 43.22, 28.26, 11.60, −0.05; HRMS (ESI) *m/z* calcd for C_21_H_29_N_4_O_3_ [M + H^+^] 385.2239, found 385.2244.

### 3.14. 2-tert-Butoxycarbonyl-6-(methylsulfonyl)-2,6-diazaspiro[3.4]octane-8-carboxylic acid (***25***)

To a solution of crude **7** (0.31 g, 1.1 mmol, 1 equiv.) in CH_2_Cl_2_ (10 mL), Et_3_N (0.14 g, 1.37 mmol, 1.25 equiv.) was added dropwise. The mixture was cooled to 0 °C, treated with dropwise addition of MsCl (0.16 g, 1.37 mmol, 1.25 equiv.), and stirred overnight. It was then washed with 10% aq. K_2_CO_3_, brine, dried over anhydrous Na_2_SO_4_, filtered, and concentrated in vacuo. The residue was dissolved in methanol (10 mL) and treated with dropwise addition of 25% aq. KOH (1 mL). The mixture was stirred for 1 h, concentrated in vacuo, and the residue was dissolved in water. The solution was acidified to pH 6 with 5% aq. HCl. The resulting precipitate was filtered off and dried over NaOH pellets to give the title compound. Yield 0.76 g (100%), white solid, m.p. 102–103 °C. ^1^H NMR (300 MHz, DMSO-*d*_6_) δ 12.94 (s, 1H), 3.99–3.68 (m, 4H), 3.46–3.29 (m, 3H), 3.22 (dd, *J* = 7.4, 6.0 Hz, 1H), 2.91 (s, 3H), 1.36 (s, 9H); ^13^C NMR (75 MHz, DMSO-*d*_6_) δ 172.53, 155.92, 79.12, 56.42, 49.37, 48.78, 41.60, 34.30, 28.45; HRMS (ESI) *m/z* calcd for C_13_H_23_N_2_O_6_S [M + H^+^] 335.1276, found 335.1280.

### 3.15. 2-tert-Butoxycarbonyl-8-(3-cyclopropyl-1,2,4-oxadiazol-5-yl)-6-(methylsulfonyl)-2,6-diazaspiro[3.4]octane (***26***)

To a solution of **25** (0.25 g, 0.75 mmol, 1 equiv.) in CH_2_Cl_2_ (10 mL), CDI (0.15 g, 0.94 mmol, 1.25 equiv.) was added and the mixture was stirred for 1 h, whereupon *N*′-hydroxycyclopropanecarboximidamide (0.094 g, 0.94 mmol, 1.25 equiv.) was added and the stirring was continued overnight. The reaction mixture was washed with 1% aq. HCl (2 × 15 mL) and concentrated in vacuo. The residue was dissolved in toluene (25 mL), TBAF (100 mg) was added, and the mixture was heated under reflux with a Dean-Stark trap for 6 h. The reaction mixture was evaporated to dryness. The residue was purified by column chromatography on silica gel using 10% methanol in CH_2_Cl_2_ to give the title compound (0.19 g, 63%) as a colorless oil. ^1^H NMR (300 MHz, CDCl_3_) δ 4.02–3.91 (m, 2H), 3.85 (t, *J* = 7.6 Hz, 1H), 3.81–3.65 (m, 5H), 3.56 (d, *J* = 9.9 Hz, 1H), 2.08 (dt, *J* = 8.3, 4.9 Hz, 1H), 1.41 (s, 9H), 1.13–0.88 (m, 4H); ^13^C NMR (75 MHz, CDCl_3_) δ 156.03, 152.70, 144.60, 80.13, 55.73, 51.38, 43.74, 41.23, 35.65, 30.95, 28.20; HRMS (ESI) *m*/*z* calcd for C_17_H_27_N_4_O_5_S [M + H^+^] 399.1702, found 399.1697.

### 3.16. 6-Benzyl-N-methyl-2-(5-nitro-2-furoyl)-2,6-diazaspiro[3.4]octane-8-carboxamide (***5a***)

General Procedure B. For the preparation of compounds **5a**–**c**, **6d**, **12**–**3**, **17**–**18**, **21**–**22**, **24** and **27**. To a solution of 5-nitro-2-furoic acid (75 mg, 0.47 mmol) in DMF (3 mL), CDI (97 mg, 0.6 mmol) was added at 0 °C and the solution was stirred for 1 h.

To a solution of **4a** (0.22 g, 0.6 mmol) in CH_2_Cl_2_ (5 mL) at 0 °C, trifluoroacetic acid (1 mL) was added and the mixture was stirred for 1 h. The solution was concentrated in vacuo while keeping the bath temperature under 30 °C. The residue was dissolved in DMF (3 mL), triethylamine (0.19 g, 1.9 mmol) was added dropwise, and after 30 min stirring, the mixture was added to the solution of 5-nitro-2-furoic acid imidazolide prepared as described above. The reaction mixture was stirred at r. t. overnight, poured into water (25 mL), and extracted with ethyl acetate (3 × 20 mL). The combined organic phases were washed with brine, dried over anhydrous Na_2_SO_4_, filtered, and concentrated in vacuo. The residue was purified by column chromatography on silica gel using 10% methanol in CH_2_Cl_2_ to give the title compound. Yield 133 mg (56%), white solid, m.p. 132–135 °C. ^1^H NMR (300 MHz, DMSO-*d*_6_) mixture of rotamers δ 8.06 (s, 1H), 7.75 (dd, *J* = 8.8, 3.9 Hz, 1H), 7.40–7.15 (m, 7H), 4.45 (dd, *J* = 27.0, 9.3 Hz, 1H), 4.31 (s, 1H), 4.10–3.74 (m, 3H), 3.67–3.51 (m, 2H), 3.07–2.82 (m, 4H), 2.75–2.52 (m, 7H), 1.37–1.14 (m, 2H), 0.96–0.73 (m, 1H); ^13^C NMR (75 MHz, DMSO-*d*_6_) mixture of rotamers δ 172.28, 156.47, 151.81, 147.95, 147.82, 139.15, 128.88, 128.85, 128.59, 127.27, 117.35, 117.31, 113.49, 113.43, 65.50, 64.72, 61.17, 59.29, 59.21, 56.69, 55.20, 51.47, 51.33, 43.53, 25.91, 25.88; HRMS (ESI) *m*/*z* calcd for C_20_H_23_N_4_O_5_ [M + H^+^] 399.1668, found 399.1673.

### 3.17. 6-Benzyl-N-isobutyl-2-(5-nitro-2-furoyl)-2,6-diazaspiro[3.4]octane-8-carboxamide (***5b***)

This compound was synthesized according to general procedure B from **4b** (0.24 g, 0.6 mmol). Yield 148 mg (56%), white solid, m.p. 166–165 °C. ^1^H NMR (300 MHz, DMSO-*d*_6_) mixture of rotamers δ 8.13 (dd, *J* = 10.8, 5.4 Hz, 1H), 7.73 (dt, *J* = 16.3, 8.2 Hz, 1H), 7.42–7.09 (m, 7H), 4.47 (dd, *J* = 33.9, 9.4 Hz, 1H), 4.37–4.24 (m, 1H), 4.12 (d, *J* = 10.3 Hz, 1H), 4.00–3.75 (m, 2H), 3.64–3.50 (m, 2H), 3.08–2.68 (m, 6H), 2.67–2.53 (m, 2H), 1.62 (dd, *J* = 11.3, 6.7 Hz, 1H), 0.89–0.61 (m, 7H); ^13^C NMR (75 MHz, DMSO-*d*_6_) mixture of rotamers, δ 196.40, 193.60, 174.10, 173.50, 171.66, 168.54, 161.80, 159.89, 156.37, 151.78, 147.97, 147.91, 146.43, 146.33, 145.57, 139.77, 139.14, 134.96, 133.64, 129.86, 129.54, 128.97, 128.85, 128.59, 128.45, 128.31, 127.87, 127.28, 121.61, 121.53, 121.50, 121.45, 120.72, 118.14, 117.31, 113.49, 113.45, 105.49, 65.02, 64.74, 60.90, 59.40, 59.30, 59.23, 56.64, 56.51, 55.38, 55.26, 51.51, 51.44, 46.53, 43.47, 28.38, 28.34, 20.44, 20.35; HRMS (ESI) *m*/*z* calcd for C_23_H_29_N_4_O_5_ [M + H^+^] 441.2137, found 441.2134.

### 3.18. 6-Benzyl-N-(cyclopropylmethyl)-2-(5-nitro-2-furoyl)-2,6-diazaspiro[3.4]octane-8-carboxamide (***5c***)

This compound was synthesized according to general procedure B from **4c** (0.185 g, 0.6 mmol). Yield 126 mg (48%), white solid, m.p. 180–181 °C. ^1^H NMR (300 MHz, DMSO-*d*_6_) mixture of rotamers δ 8.42–8.08 (m, 1H), 7.76 (dd, *J* = 8.9, 3.9 Hz, 1H), 7.45–7.11 (m, 8H), 4.62–4.26 (m, 2H), 4.14 (d, *J* = 10.4 Hz, 1H), 3.94 (d, *J* = 9.2 Hz, 1H), 3.81 (d, *J* = 11.1 Hz, 1H), 3.69–3.47 (m, 3H), 3.14–2.77 (m, 6H), 2.62 (dd, *J* = 16.4, 8.9 Hz, 2H), 1.00–0.70 (m, 1H), 0.33 (dd, *J* = 18.6, 7.1 Hz, 2H), 0.10 (dd, *J* = 9.1, 4.1 Hz, 2H).; ^13^C NMR (75 MHz, DMSO-*d*_6_) mixture of rotamers, δ 171.59, 156.38, 151.79, 147.97, 147.91, 139.16, 128.88, 128.85, 128.74, 128.60, 127.27, 117.32, 113.46, 65.22, 64.65, 61.03, 59.30, 59.22, 56.51, 56.35, 55.26, 51.34, 43.54, 43.34, 43.30, 11.33, 11.30, 3.66, 3.55, 3.46; HRMS (ESI) *m*/*z* calcd for C_23_H_27_N_4_O_5_ [M + H^+^] 439.1981, found 439.1986.

### 3.19. 1-[6-Benzyl-2-(5-nitro-2-furoyl)-2,6-diazaspiro[3.4]oct-8-yl]-N,N-dimethylmethanamine (***6d***)

This compound was synthesized according to general procedure B from **5d** (0.215 g, 0.6 mmol). Yield 91 mg (38%), white solid, m.p. 154–155 °C. ^1^H NMR (300 MHz, DMSO-*d*_6_) mixture of rotamers δ 7.77 (d, *J* = 1.9 Hz, 1H), 7.76 (d, *J* = 1.9 Hz, 1H), 7.43–7.15 (m, 14H), 4.68 (d, *J* = 9.5 Hz, 1H), 4.39 (s, 2H), 4.31 (d, *J* = 10.4 Hz, 1H), 4.17 (d, *J* = 9.6 Hz, 1H), 3.94 (q, *J* = 10.1 Hz, 2H), 3.70 (d, *J* = 10.4 Hz, 1H), 3.63–3.48 (m, 5H), 2.98–2.55 (m, 8H), 2.44–2.25 (m, 5H), 2.10 (t, *J* = 10.9 Hz, 17H); ^13^C NMR (75 MHz, DMSO-*d*_6_) mixture of rotamers, δ 156.64, 156.48, 151.80, 148.01, 147.96, 139.28, 135.52, 128.84, 128.57, 127.21, 117.20, 113.45, 65.42, 65.36, 64.58, 61.25, 61.13, 60.33, 59.67, 58.65, 58.48, 58.27, 54.17, 45.74, 45.69, 43.23, 43.14, 41.77, 41.47.; HRMS (ESI) *m*/*z* calcd for C_21_H_27_N_4_O_5_ [M + H^+^] 399.2032, found 399.2028.

### 3.20. 8-(1-Benzyl-5-methyl-1H-imidazol-2-yl)-6-methyl-2-(5-nitro-2-furoyl)-2,6-diazaspiro[3.4]octane (***12***)

This compound was synthesized according to general procedure B from **10** (0.237 g, 0.6 mmol). Yield 74 mg (28%), white solid, m.p. 165–167 °C. ^1^H NMR (300 MHz, DMSO-*d*_6_) mixture of rotamers δ 7.74 (dd, *J* = 13.4, 3.8 Hz, 1H), 7.28–7.06 (m, 3H), 7.02–6.81 (m, 3H), 6.73 (d, *J* = 7.7 Hz, 1H), 5.37–5.06 (m, 2H), 4.24 (d, *J* = 9.5 Hz, 1H), 4.15 (d, *J* = 10.2 Hz, 1H), 3.94–3.53 (m, 4H), 3.38 (d, *J* = 19.3 Hz, 4H), 3.17–2.94 (m, 2H), 2.76–2.54 (m, 2H), 2.46–2.36 (m, 1H), 2.23 (s, 3H), 2.17–2.00 (m, 4H); ^13^C NMR (75 MHz, DMSO-*d*_6_) mixture of rotamers, δ 155.99, 151.74, 151.57, 148.01, 147.85, 147.65, 138.48, 138.23, 128.98, 128.91, 127.69, 127.51, 127.38, 127.19, 126.15, 125.97, 125.77, 125.72, 117.17, 117.12, 113.46, 68.02, 67.52, 65.66, 63.11, 62.97, 62.02, 58.47, 55.04, 45.96, 45.87, 45.24, 45.10, 43.51, 43.40, 41.95, 41.87.; HRMS (ESI) *m/z* calcd for C_23_H_26_N_5_O_4_ [M + H^+^] 436.1984, found 436.1988.

### 3.21. 6-Methyl-8-(5-methyl-1,3-oxazol-2-yl)-2-(5-nitro-2-furoyl)-2,6-diazaspiro[3.4]octane (***13***)

This compound was synthesized according to general procedure B from **11** (0.184 g, 0.6 mmol). Yield 99 mg (48%), white solid, m.p. 175–176 °C. ^1^H NMR (300 MHz, DMSO-*d*_6_) mixture of rotamers δ 7.77 (d, *J* = 3.9 Hz, 1H), 7.72 (d, *J* = 3.9 Hz, 1H), 7.32 (d, *J* = 3.9 Hz, 1H), 7.27 (d, *J* = 3.9 Hz, 1H), 7.02 (s, 1H), 6.80–6.73 (m, 2H), 4.57 (q, *J* = 9.6 Hz, 2H), 4.31 (d, *J* = 10.1 Hz, 1H), 4.17 (d, *J* = 10.4 Hz, 1H), 4.05 (d, *J* = 10.4 Hz, 1H), 3.97 (d, *J* = 10.0 Hz, 1H), 3.84–3.64 (m, 3H), 3.55–3.44 (m, 4H), 3.12–2.94 (m, 4H), 2.76 (ddd, *J* = 17.3, 7.5, 4.9 Hz, 4H), 2.30 (s, 5H), 2.25 (s, 3H), 2.22 (s, 2H); ^13^C NMR (75 MHz, DMSO-*d*_6_) mixture of rotamers, δ 162.41, 162.31, 156.55, 156.43, 151.85, 149.25, 149.19, 147.67, 147.61, 122.93, 117.41, 113.42, 113.36, 66.17, 64.16, 59.94, 59.25, 58.90, 58.79, 55.23, 45.46, 45.41, 44.98, 41.89, 10.82.; HRMS (ESI) *m/z* calcd for C_16_H_19_N_4_O_5_ [M + H^+^] 347.1355, found 347.1360.

### 3.22. 6-(Methylsulfonyl)-8-(4-methyl-4H-1,2,4-triazol-3-yl)-2-(5-nitro-2-furoyl)-2,6-diazaspiro[3.4]octane (***17***)

This compound was synthesized according to general procedure B from **16** (0.22 g, 0.6 mmol). Yield 138 mg (56%), white solid, m.p. 180–182 °C. ^1^H NMR (300 MHz, DMSO-*d*_6_) mixture of rotamers δ 8.44 (s, 1H), 7.78 (d, *J* = 3.9 Hz, 1H), 7.73 (d, *J* = 3.9 Hz, 1H), 7.30 (t, *J* = 4.1 Hz, 1H), 4.57 (q, *J* = 9.5 Hz, 2H), 4.21–3.84 (m, 5H), 3.84–3.58 (m, 6H), 3.56–3.37 (m, 1H), 2.85 (d, *J* = 9.7 Hz, 3H); ^13^C NMR (75 MHz, DMSO-*d*_6_) mixture of rotamers, δ 156.67, 152.31, 151.83, 147.80, 147.70, 145.65, 117.39, 113.50, 113.45, 62.50, 59.36, 57.62, 56.55, 55.27, 53.97, 51.20, 51.05, 44.12, 43.99, 34.59, 34.52, 30.92.; HRMS (ESI) *m/z* calcd for C_15_H_19_N_6_O_6_S [M + H^+^] 411.1086, found 411.1091.

### 3.23. 6-Benzyl-8-(4-methyl-4H-1,2,4-triazol-3-yl)-2-(5-nitro-2-furoyl)-2,6-diazaspiro[3.4]octane (***18***)

This compound was synthesized according to general procedure B from **15** (0.23 g, 0.6 mmol). Yield 157 mg (62%), white solid, m.p. 152–153 °C. ^1^H NMR (300 MHz, DMSO-*d*_6_) mixture of rotamers δ 8.65–8.20 (m, 1H), 7.84–7.59 (m, 2H), 7.57–7.16 (m, 5H), 7.02 (s, 1H), 4.77–3.96 (m, 4H), 3.94–3.06 (m, 13H), 2.85 (dd, *J* = 21.6, 9.0 Hz, 1H), 2.68 (dd, *J* = 16.7, 8.9 Hz, 1H); ^13^C NMR (75 MHz, DMSO-*d*_6_) mixture of rotamers, δ 156.60, 154.43, 154.32, 151.79, 147.75, 145.30, 139.09, 135.51, 129.86, 129.54, 128.91, 128.86, 128.62, 127.32, 122.04, 117.32, 113.43, 65.48, 64.63, 64.49, 61.43, 59.33, 58.92, 58.72, 54.82, 44.51, 30.77; HRMS (ESI) *m/z* calcd for C_21_H_23_N_6_O_4_ [M + H^+^] 423.1780, found 423.1777.

### 3.24. 6-Benzyl-8-(5-methyl-4H-1,2,4-triazol-3-yl)-2-(5-nitro-2-furoyl)-2,6-diazaspiro[3.4]octane (***21***)

This compound was synthesized according to general procedure B from **19** (0.23 g, 0.6 mmol). Yield 108 mg (43%), white solid, m.p. 168–170 °C. ^1^H NMR (300 MHz, DMSO-*d*_6_) mixture of rotamers δ 8.57–8.26 (m, 1H), 7.87–7.60 (m, 2H), 7.52–7.13 (m, 6H), 7.02 (s, 1H), 4.82–3.93 (m, 4H), 3.89–3.52 (m, 7H), 3.27–3.04 (m, 2H), 2.99–2.55 (m, 3H); ^13^C NMR (75 MHz, DMSO-*d*_6_) mixture of rotamers δ 158.23, 156.60, 154.43, 154.32, 151.79, 147.91, 147.75, 145.30, 139.09, 135.51, 129.86, 129.54, 128.91, 128.86, 128.62, 127.82, 127.32, 122.04, 117.32, 113.43, 66.85, 65.48, 64.63, 64.49, 61.43, 59.33, 58.92, 58.72, 54.82, 45.93, 44.51, 43.31, 30.77.; HRMS (ESI) *m/z* calcd for C_21_H_23_N_6_O_4_ [M + H^+^] 423.1780, found 423.1784.

### 3.25. 6-Benzyl-8-(5-cyclopropyl-4H-1,2,4-triazol-3-yl)-2-(5-nitro-2-furoyl)-2,6-diazaspiro[3.4]octane (***22***)

This compound was synthesized according to general procedure B from **20** (0.24 g, 0.6 mmol). Yield 102 mg (38%), white solid, m.p. 155–157 °C. ^1^H NMR (300 MHz, DMSO-*d*_6_) mixture of rotamers δ 13.39 (s, 1H), 7.82–7.58 (m, 1H), 7.41–7.10 (m, 5H), 4.59–4.39 (m, 1H), 4.26–3.85 (m, 2H), 3.73–3.57 (m, 2H), 3.14–2.60 (m, 3H), 2.06–1.69 (m, 1H), 1.06–0.49 (m, 4H); 13C NMR (75 MHz, DMSO-*d*_6_) mixture of rotamers δ 193.60, 167.18, 161.07, 156.43, 156.30, 151.73, 147.85, 147.80, 139.26, 135.51, 134.96, 129.86, 129.54, 128.87, 128.60, 127.26, 121.70, 121.63, 121.57, 121.54, 121.50, 121.48, 118.46, 117.24, 113.44, 113.38, 64.18, 64.10, 63.66, 59.53, 59.38, 57.37, 57.29, 55.38, 55.26, 44.29, 44.20, 9.48, 8.21, 7.60; HRMS (ESI) *m/z* calcd for C_23_H_25_N_6_O_4_ [M + H^+^] 449.1937, found 449.1942.

### 3.26. 6-Benzyl-8-(3-methyl-1,2,4-oxadiazol-5-yl)-2-(5-nitro-2-furoyl)-2,6-diazaspiro[3.4]octane (***24***)

This compound was synthesized according to general procedure B from **23** (0.245 g, 0.6 mmol). Yield 121 mg (48%), white solid, m.p. 180–182 °C. ^1^H NMR (300 MHz, DMSO-*d*_6_) mixture of rotamers δ 7.84–7.63 (m, 1H), 7.41–7.09 (m, 5H), 4.75–4.42 (m, 1H), 4.27 (dd, *J* = 36.8, 10.2 Hz, 1H), 4.10–3.93 (m, 2H), 3.82 (t, *J* = 11.6 Hz, 0H), 3.74–3.50 (m, 2H), 3.10 (dt, *J* = 26.4, 20.0, 9.4 Hz, 2H), 2.92–2.75 (m, 2H), 2.31 (d, *J* = 8.0 Hz, 3H); ^13^C NMR (75 MHz, DMSO-*d*_6_) mixture of rotamers δ 179.23, 179.17, 167.29, 167.22, 162.72, 156.61, 151.85, 147.72, 147.58, 138.82, 129.86, 129.54, 129.00, 128.87, 128.66, 127.39, 121.59, 117.42, 113.44, 63.90, 63.78, 59.79, 59.04, 56.78, 56.66, 55.25, 55.16, 44.20, 43.12, 36.17, 31.17, 11.52.; HRMS (ESI) *m/z* calcd for C_21_H_22_N_5_O_5_ [M + H^+^] 424.1620, found 424.1616.

### 3.27. 8-(3-Cyclopropyl-1,2,4-oxadiazol-5-yl)-6-(methylsulfonyl)-2-(5-nitro-2-furoyl)-2,6-diazaspiro[3.4]octane (***27***)

This compound was synthesized according to general procedure B from **26** (0.24 g, 0.6 mmol). Yield 165 mg (63%), white solid, m.p. 171–173 °C. ^1^H NMR (300 MHz, DMSO-*d*_6_) mixture of rotamers δ 7.77 (dd, *J* = 9.8, 3.9 Hz, 1H), 7.31 (dd, *J* = 8.0, 3.9 Hz, 1H), 4.65 (q, *J* = 9.9 Hz, 1H), 4.45 (dd, *J* = 25.5, 10.0 Hz, 1H), 4.26–4.07 (m, 2H), 3.97 (dt, *J* = 35.2, 9.1 Hz, 1H), 3.82–3.52 (m, 4H), 2.97 (d, *J* = 1.7 Hz, 3H), 2.18–2.02 (m, 1H), 1.07–0.68 (m, 4H); ^13^C NMR (75 MHz, DMSO-*d*_6_) mixture of rotamers δ 177.00, 176.87, 172.19, 156.67, 151.86, 147.63, 147.52, 122.80, 122.67, 122.56, 117.49, 113.48, 60.70, 57.56, 55.56, 55.26, 54.21, 49.35, 49.15, 43.77, 43.71, 42.72, 42.54, 34.65, 34.59, 7.89, 6.62; HRMS (ESI) *m/z* calcd for C_17_H_20_N_5_O_7_S [M + H^+^] 438.1083, found 438.1088.

### 3.28. Method to Evaluate Antitubercular Activity of Compounds ***5a**–**c***, ***6d***, ***12**–**13***, ***17**–**18***, ***21**–**22***, ***24***, and ***27***

*Mycobacterium tuberculosis* H_37_Rv strain (originated from the Institute of Hygiene and Epidemiology in Prague, 1976) was obtained on 7 August 2013 from the Federal Scientific Center for Expertise of Medical Products (RF Ministry of Health Care). The lyophilized strain was seeded in Löwenstein–Jensen growth medium. After 3 weeks, the culture was suspended in physiological solution containing glycerol (15%), transferred into cryotubes, and kept at −80 °C. Three weeks prior to the experiment, the culture was brought to ambient temperature and re-seeded into Löwenstein–Jensen growth medium. Thus, the 2nd generation of the original *M. tuberculosis* culture was used in the present study.

The minimal inhibitory concentration (MIC) of each compound was determined using the REMA (resazurin microtiter plate assay) [13]. A 3-week *M. tuberculosis* culture was transferred into a dry, sterile tube containing 8–9 3 mm glass beads. The tube was placed on a Vortex shaker for 30–40 s and then 5 mL Middlebrook 7H9 Broth (Becton Dickinson, catalogue No. 271310) was introduced. The turbidity of the bacterial suspension was adjusted to 1.0 McFarland units (corresponding to approximately 3 × 10^8^ bacteria/mL) and diluted 20-fold with Middlebrook 7H9 Broth containing OADC enrichment (Becton Dickinson, catalogue No. 245116). The same culture medium was used to prepare the 1:100 *M. tuberculosis* (1% population) control. The stock solution of the compound in DMSO (10 mg/mL) was diluted with Middlebrook 7H9 Broth (containing OADC enrichment) to a concentration of 100 μg/mL. A 200 μL aliquot of the solution thus obtained was introduced into the 1st row of a 96-well microtiter plate. This row was used to perform 2-fold serial dilutions using an 8-channel pipette to obtain final concentrations of 0.4, 0.8, 1.6, 3.1, 6.2, 12.5, 25, and 50 μg/mL of the compound in rows 2–9 (accounting for 100 μL of bacterial suspension introduced for testing). Similarly, an experiment with 0.004, 0.008, 0.016, 0.031, 0.062, 0.125, 0.25, 0.50 and 1 μg/mL concentrations of the compounds in rows 1–9 was carried out. Row 10 contained the *MTb* suspension control while row 11 contained the same culture diluted 10-fold (1% control). Row 12 was used as a blank control for the optical density reading (200 μL of growth medium). The bacterial suspension (100 μL) was introduced into each well, except rows 11 (1% population control) and 12 (blank culture medium), to give a total volume of 200 μL in each well. The plates were incubated at 35 °C for 7 days. At that point, 0.01% aqueous solution (30 μL) of resazurin (Sigma, product No. R7017) was introduced in each well and the incubation continued for 18 h at 35 °C. The fluorescence reading was performed using a FLUOstar Optima plate reader operating at λ_ex_ = 520 nm and λ_em_ = 590 nm. The bacterial viability was determined by comparing the mean values (±SD at *p* = 0.05) of fluorescence in the control wells (row 12, blank and row 11, 1% control) and the wells containing the compound tested. The MIC was determined as the compound concentration at which the fluorescence reached a plateau or was statistically (t criterion) similar to that of the 1% control (see Supplementary Materials).

## 4. Conclusions

In conclusion, by elaborating a small set of twelve compounds from readily available 2,6-diazaspiro[3.4]octane building block **3** and exploring diverse variants of the molecular periphery, including various azole substituents, we identified a remarkably potent antitubercular lead displaying a minimal inhibitory concentration of 0.016 μg/mL. This compound will be tested in vitro at a lower concentration range and in vivo to determine its efficacy and safety. The results of these studies will be reported in due course.

## Figures and Tables

**Table 1:** Yields and growth inhibitory activity of 5-nitrofuroyl derivatives against drug-sensitive *M. tuberculosis* H37Rv strain.

Compound	Yield, %	MIC, μg/mL *
**5a**	56	25
**5b**	56	50
**5c**	48	100
**6d**	38	>100
**12**	28	25
**13**	48	>100
**17**	56	0.016
**18**	62	100
**21**	43	50
**22**	38	50
**24**	48	6.2
**27**	63	>100

* Determined chromogenically in 6 repeated experiments.

## Data Availability

Data available from the corresponding authors upon reasonable request.

## Supplementary Materials

The following supporting information can be downloaded at https://www.mdpi.com/article/10.3390/molecules28062529/s1: copies of the NMR spectra, images of exemplary assay plates.
