# Supplementary material for: Periphery Exploration around 2,6-Diazaspiro[3.4]octane Core Identifies a Potent Nitrofuran Antitubercular Lead
Source: Molecules. 2023 Mar 10;28(6):2529. doi: 10.3390/molecules28062529 (PMC10056547; doi:10.3390/molecules28062529)

Supplementary Materials for

Periphery exploration around 2,6-diazaspiro[3.4]octane core identifies potent antitubercular lead

Alexei Lukin, Kristina Komarova, Lyubov Vinogradova, Marine Dogonadze,

Tatiana Vinogradova, Piotr Yablonsky, Alexander Kazantsev and Mikhail Krasavin*

Contents

Copies of ^{1}H and ^{13}C NMR spectra	S2
Images of exemplary testing plates	S27

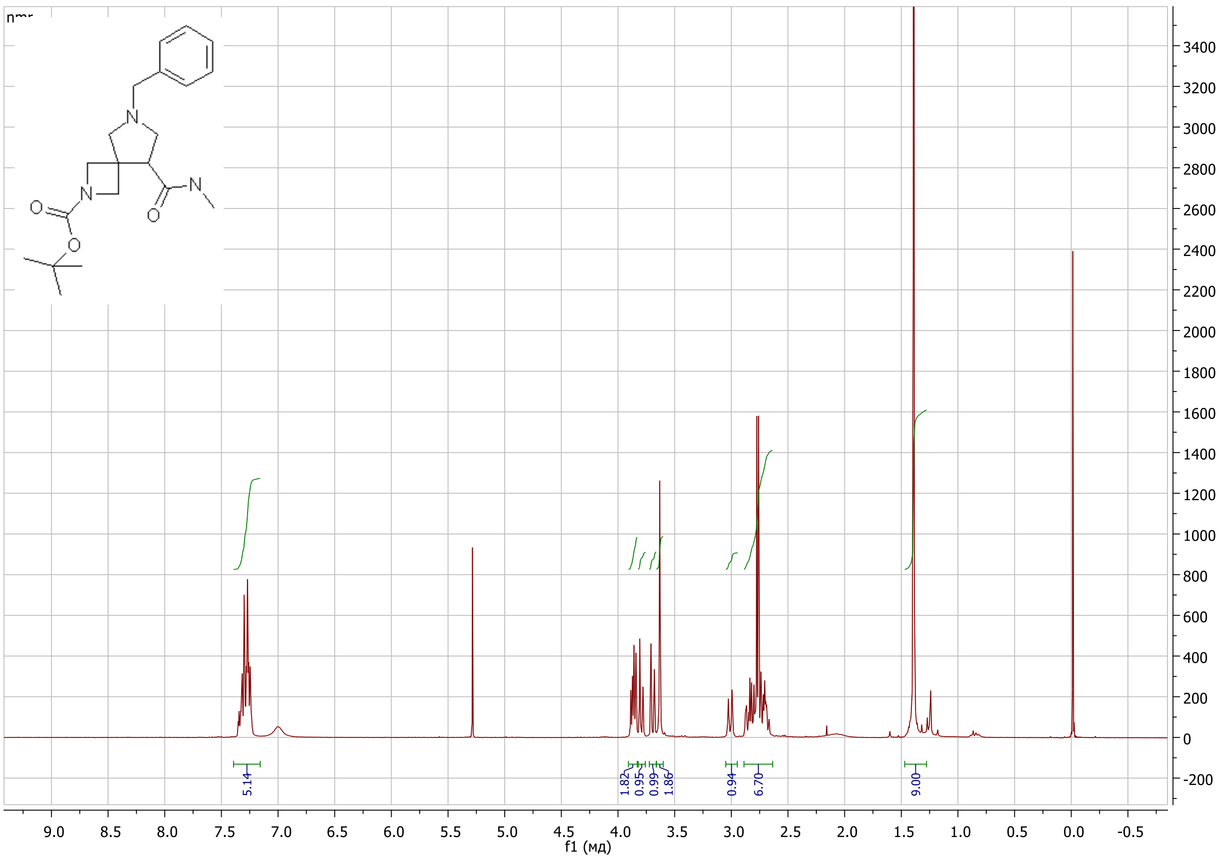

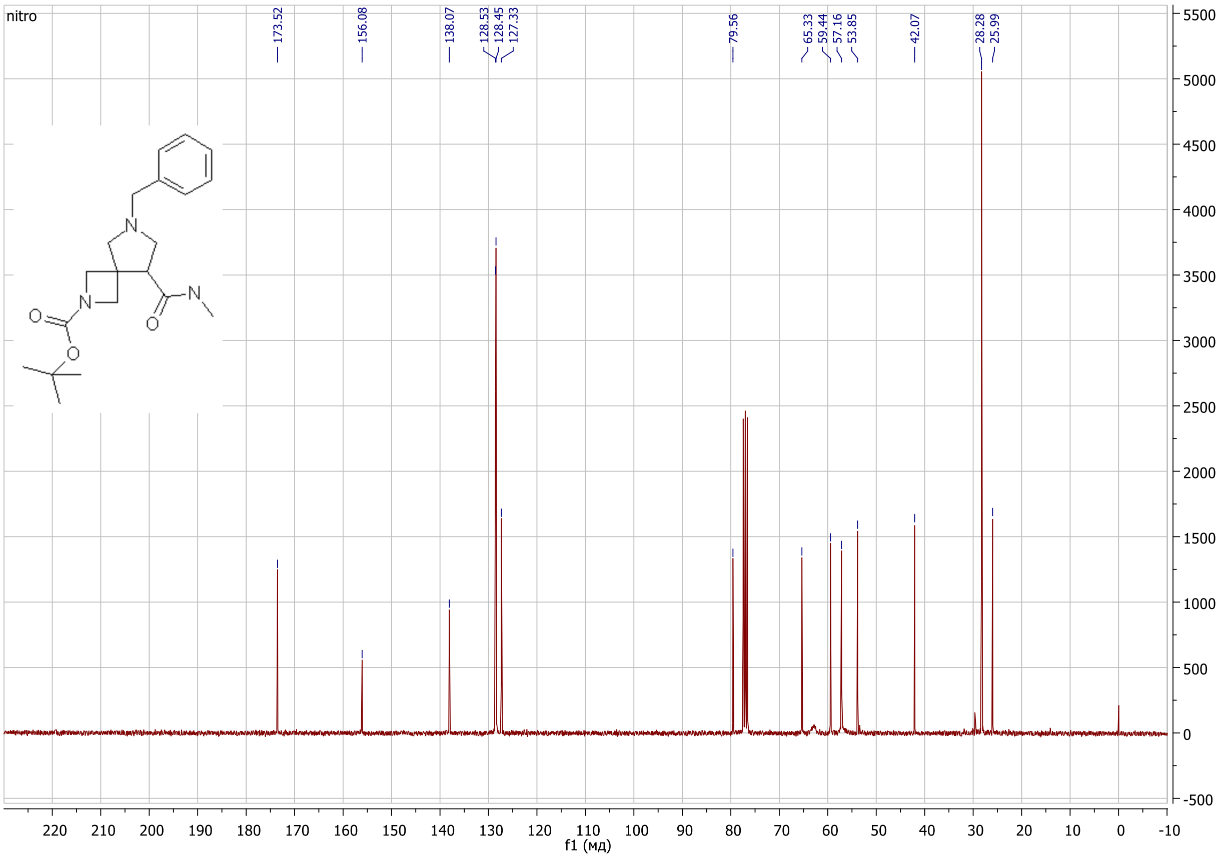

^{1}H and ^{13}C NMR spectra for compound **4a**

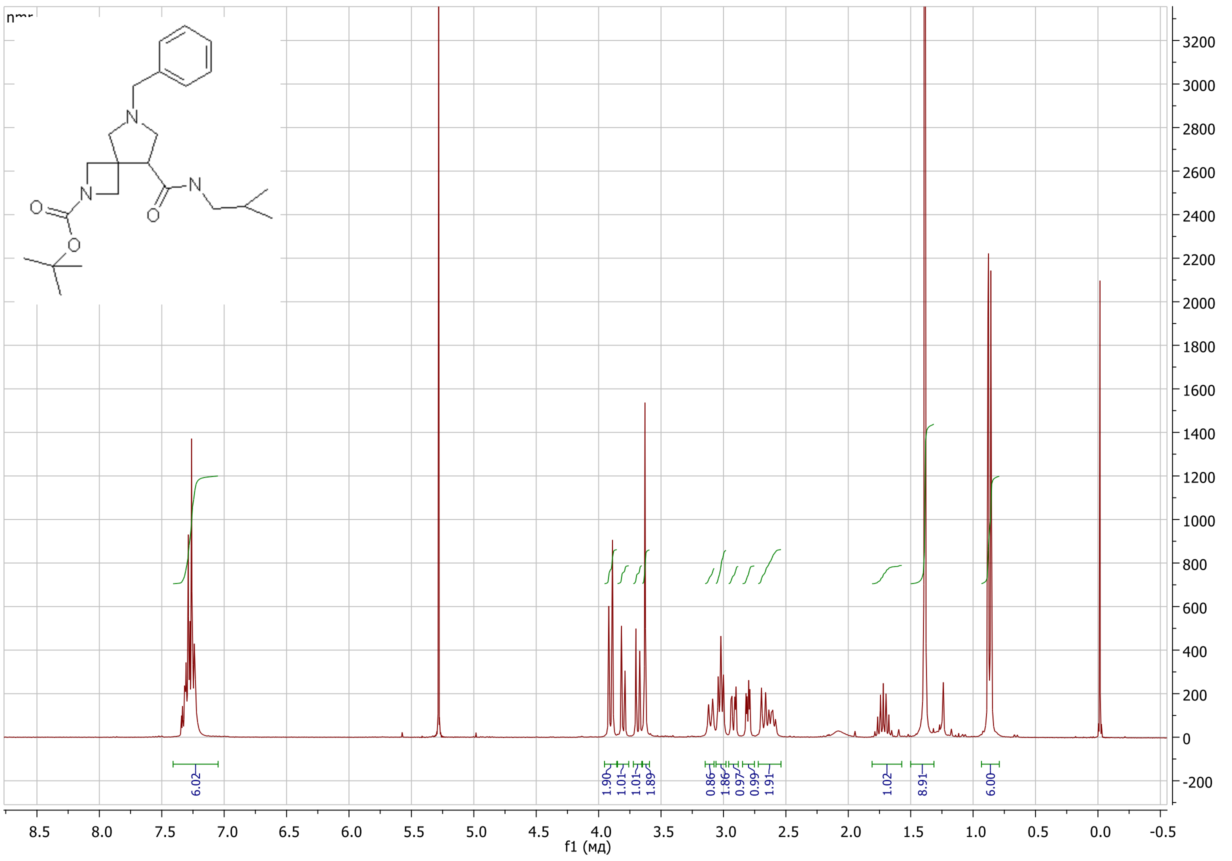

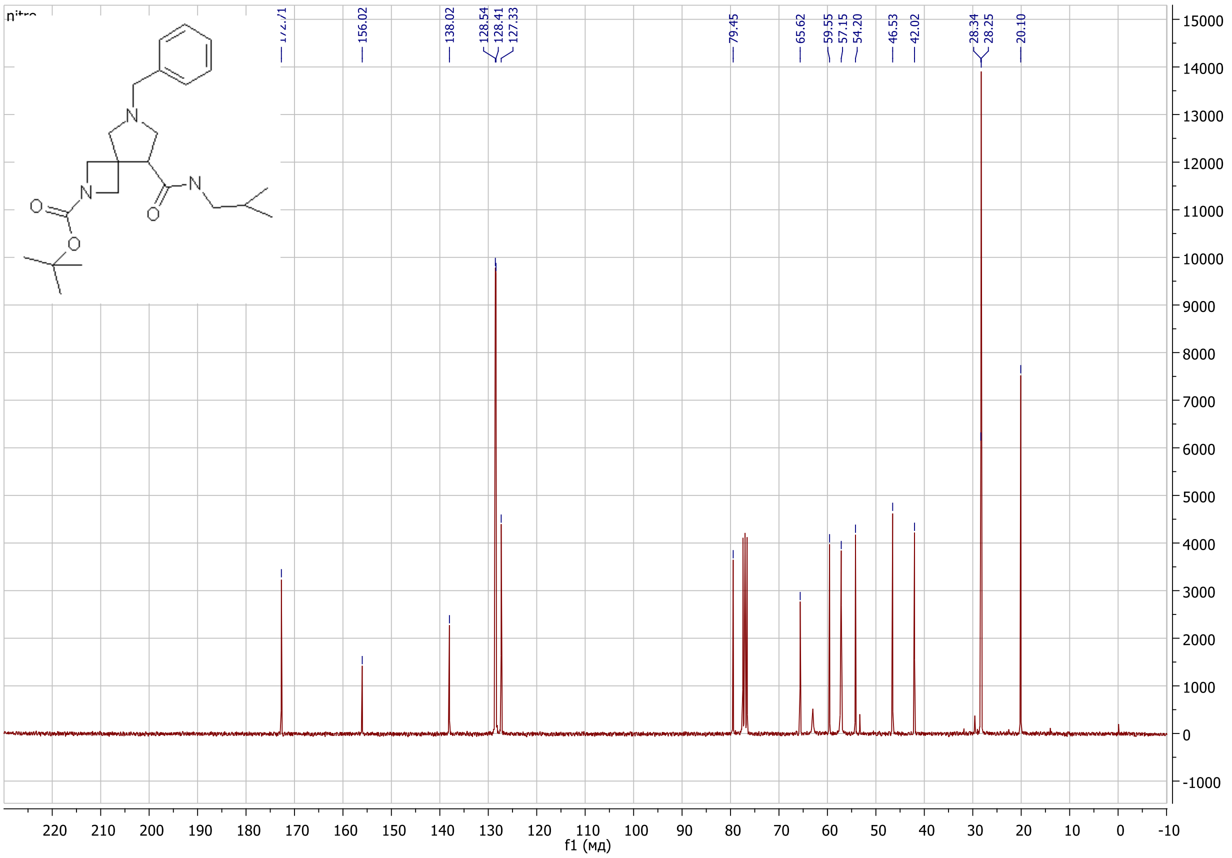

^{1}H and ^{13}C NMR spectra for compound **4b**

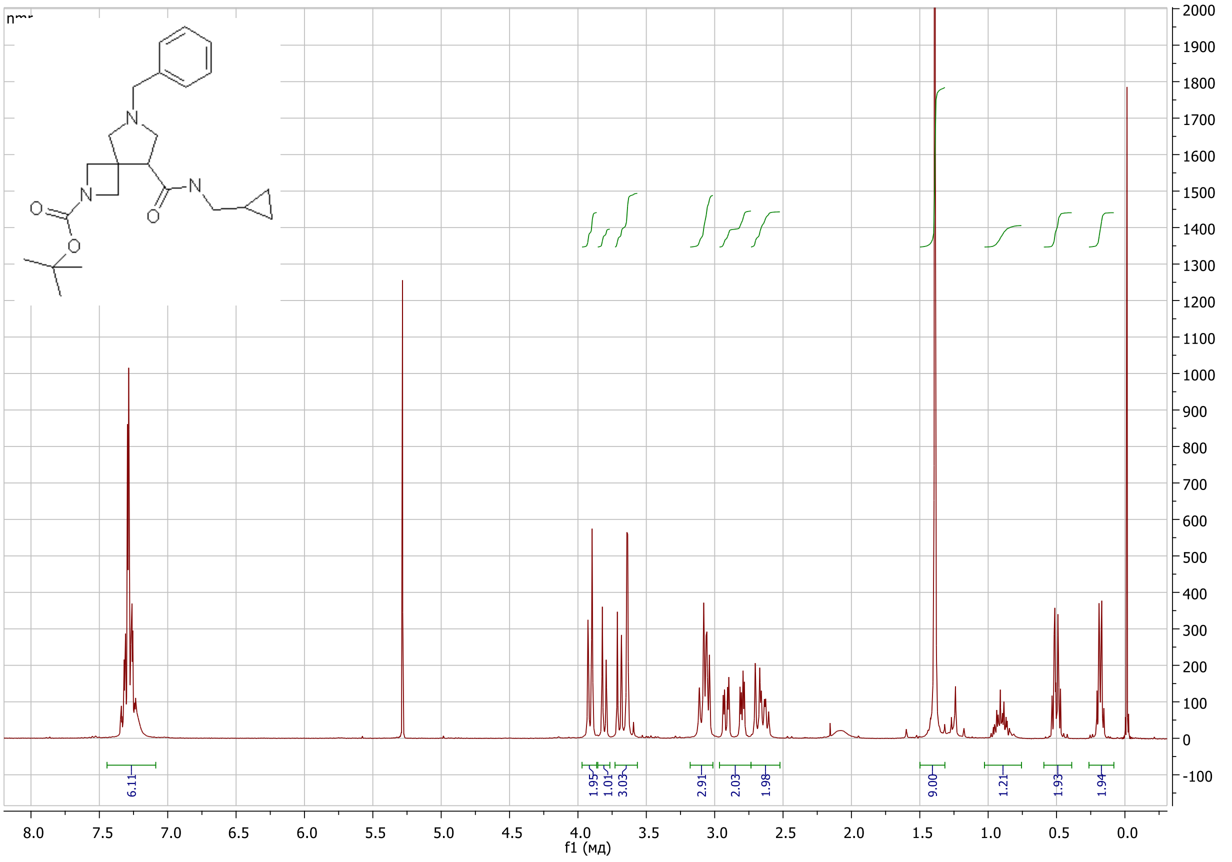

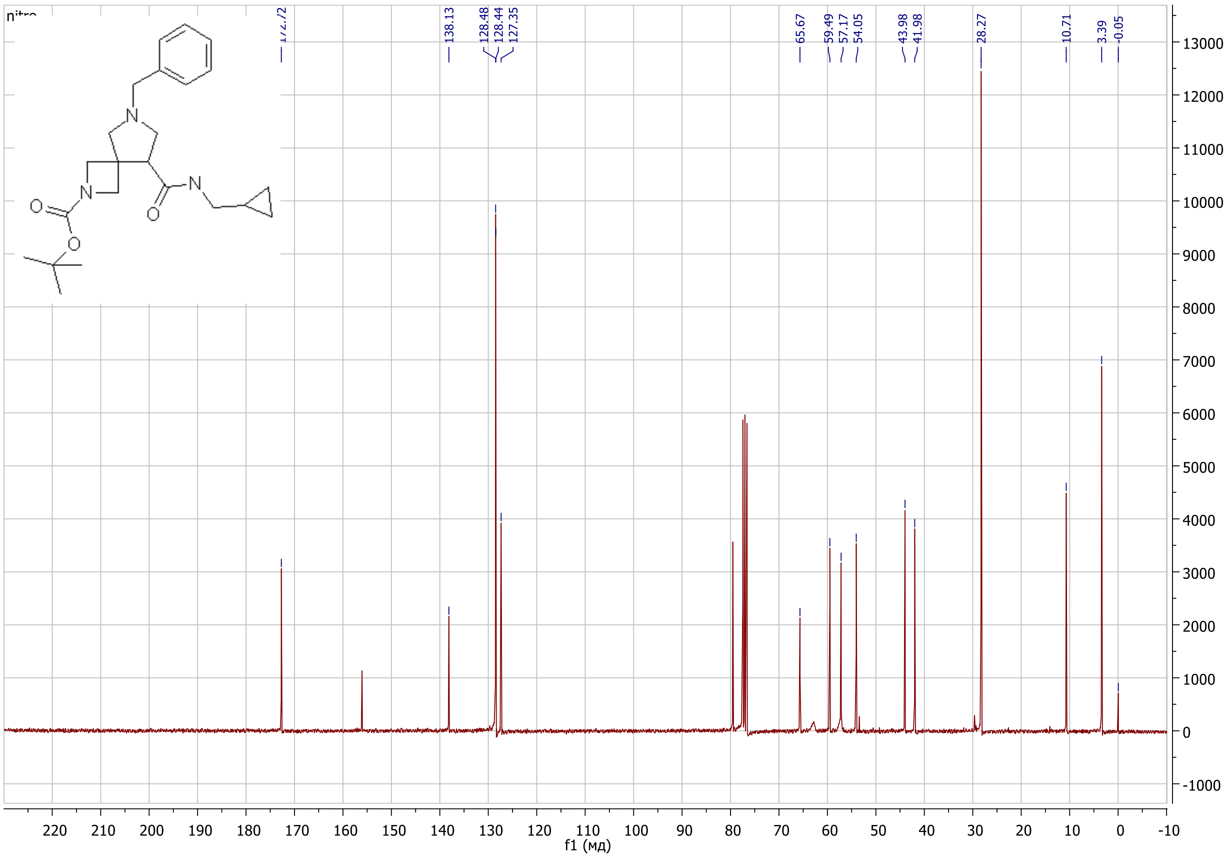

^{1}H and ^{13}C NMR spectra for compound **4c**

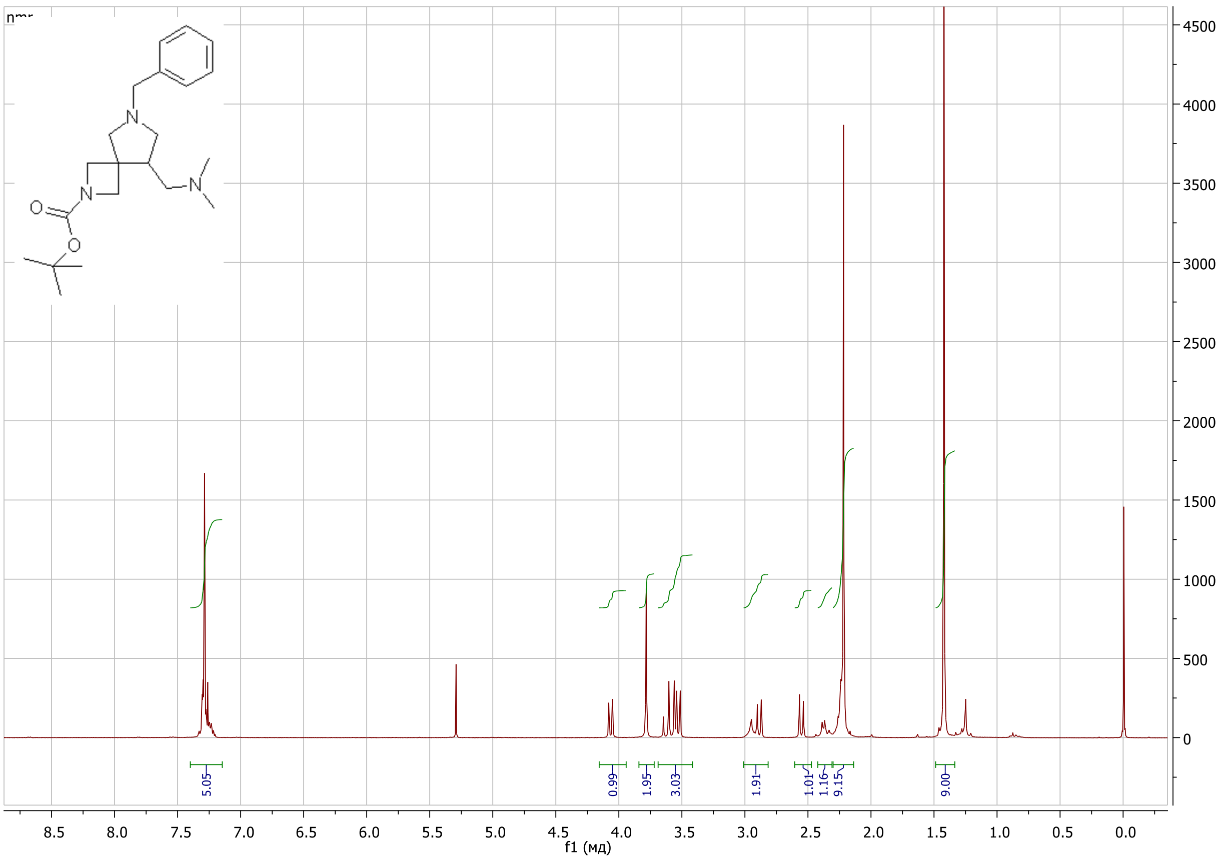

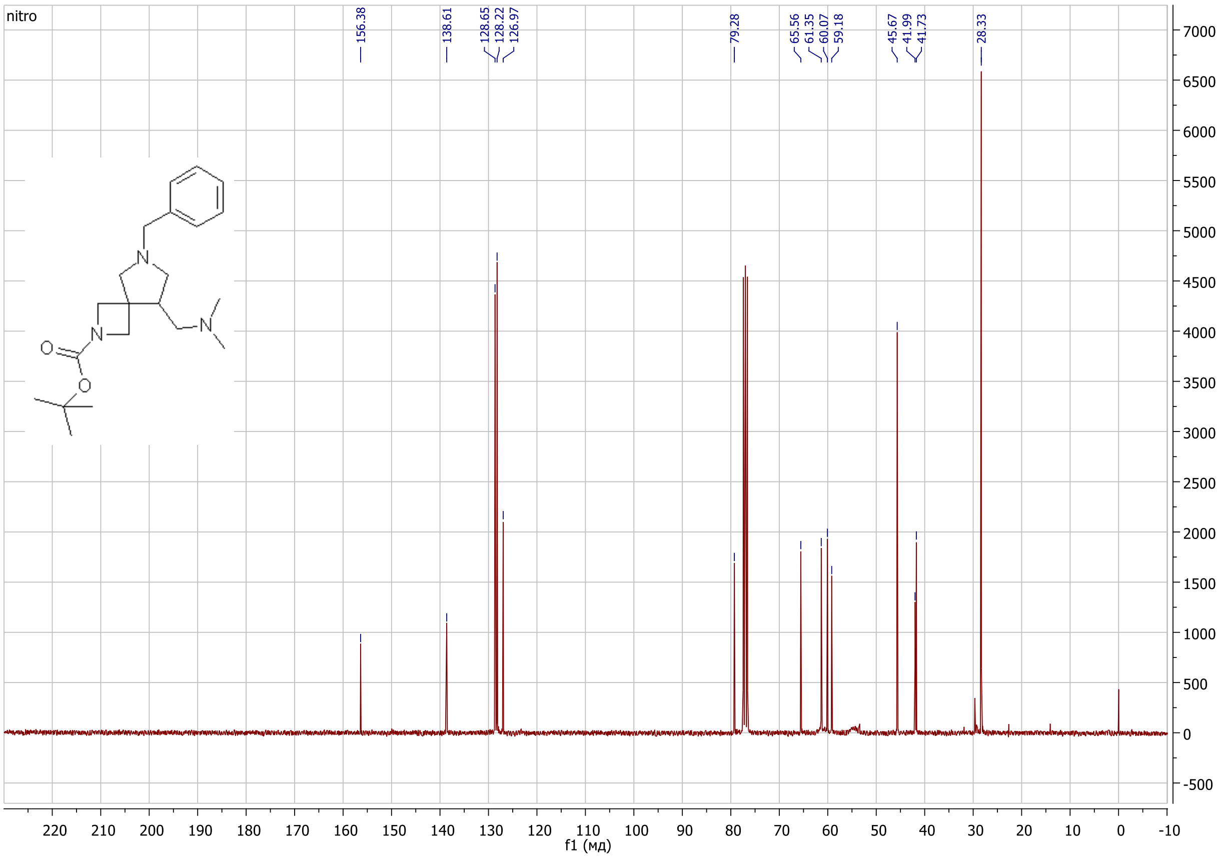

^{1}H and ^{13}C NMR spectra for compound **5d**

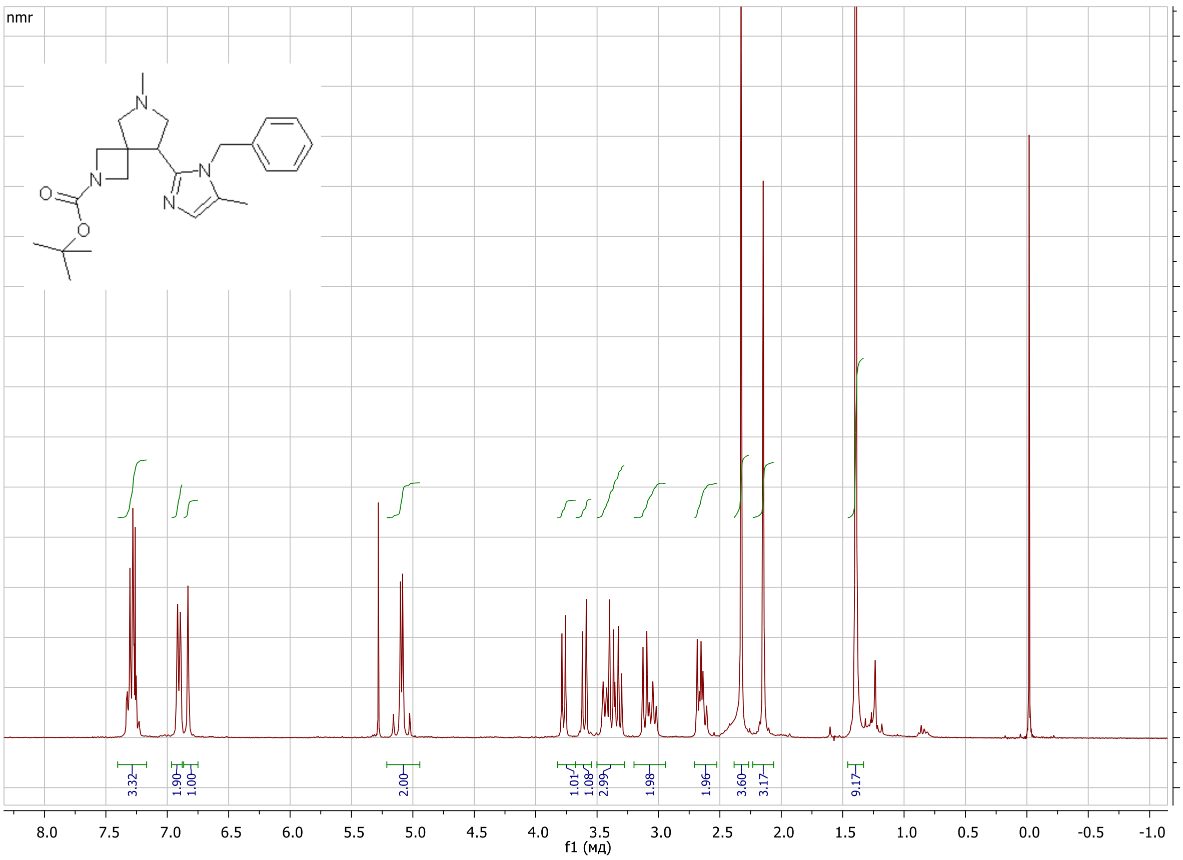

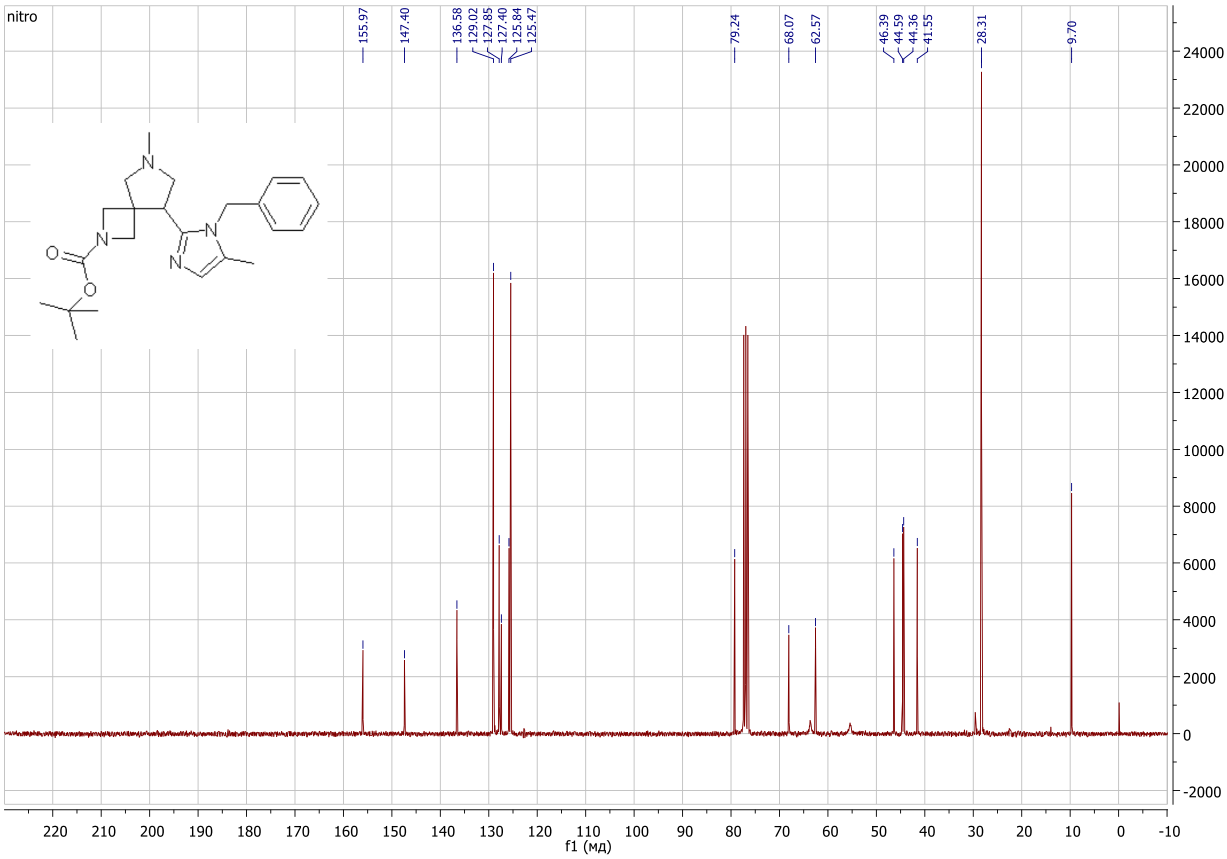

^{1}H and ^{13}C NMR spectra for compound **10**

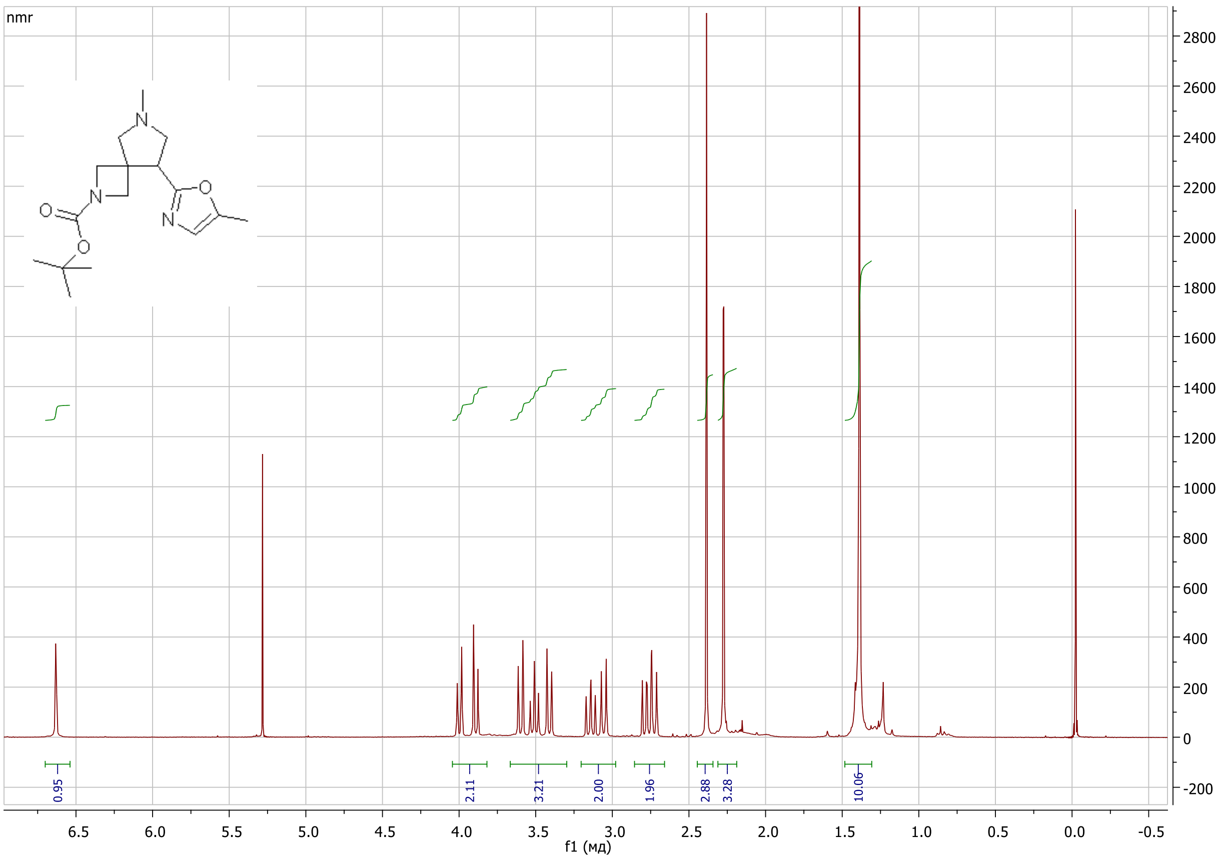

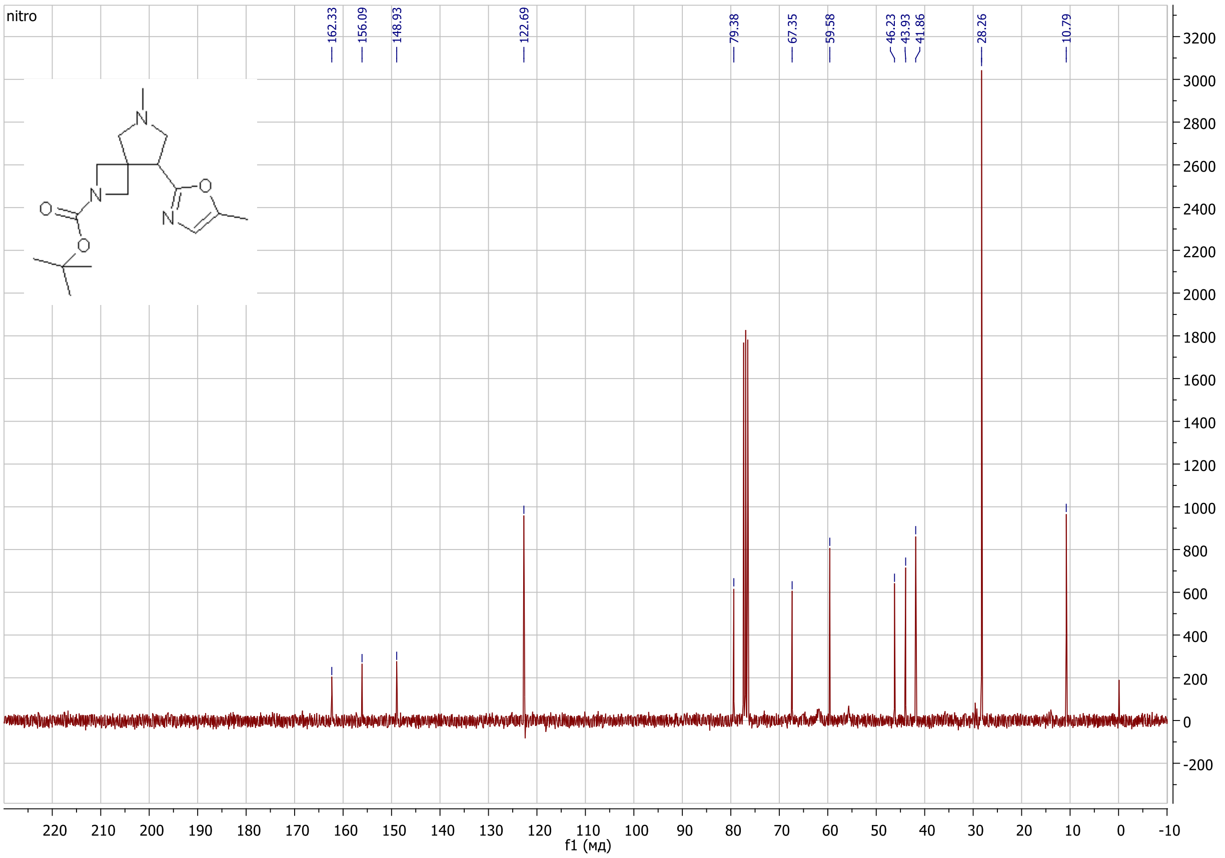

^{1}H and ^{13}C NMR spectra for compound **11**

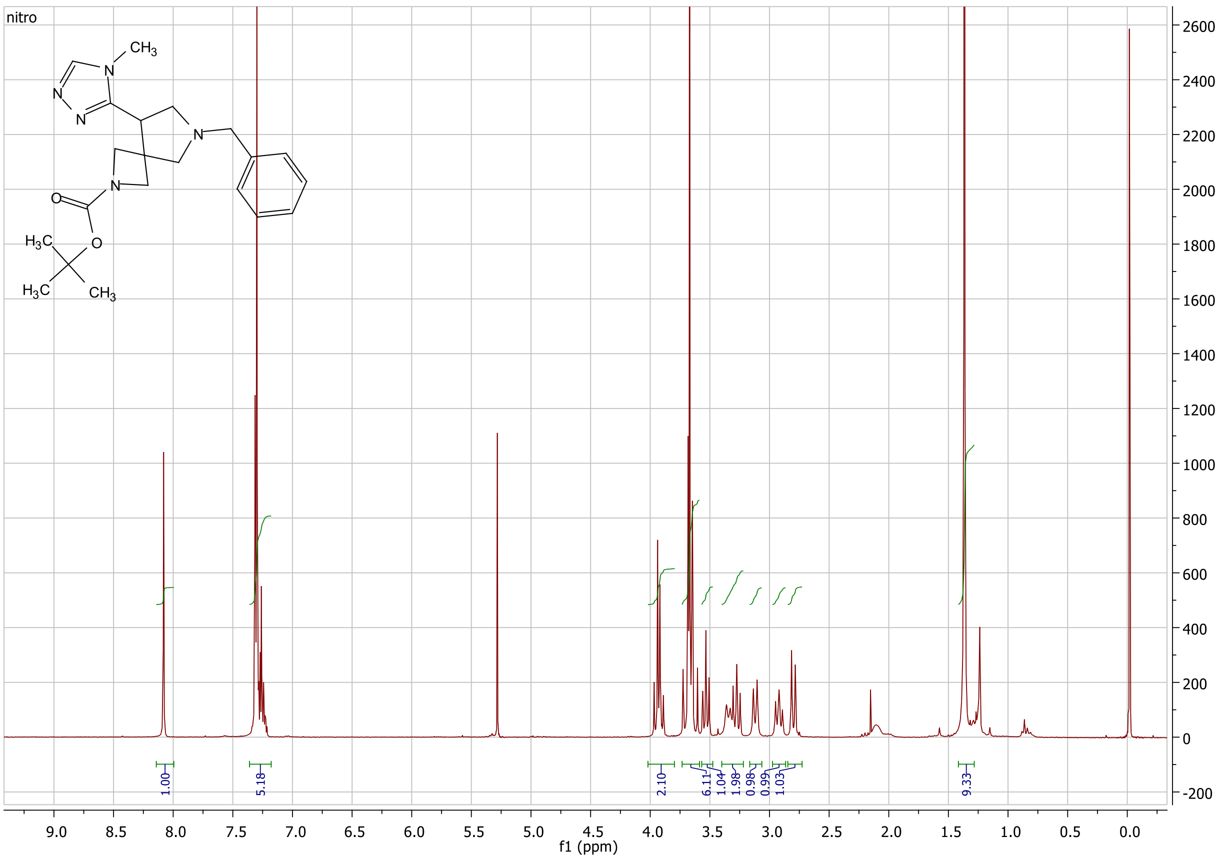

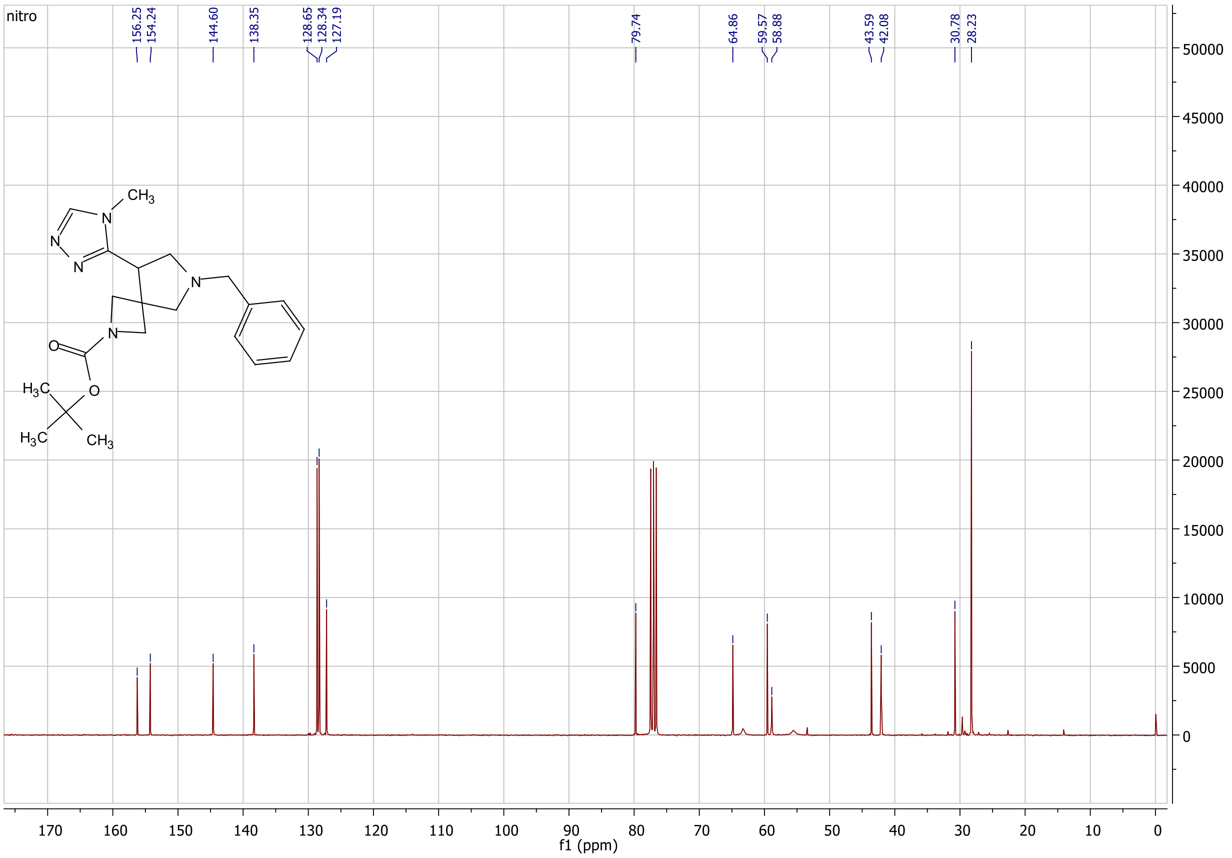

^{1}H and ^{13}C NMR spectra for compound **15**

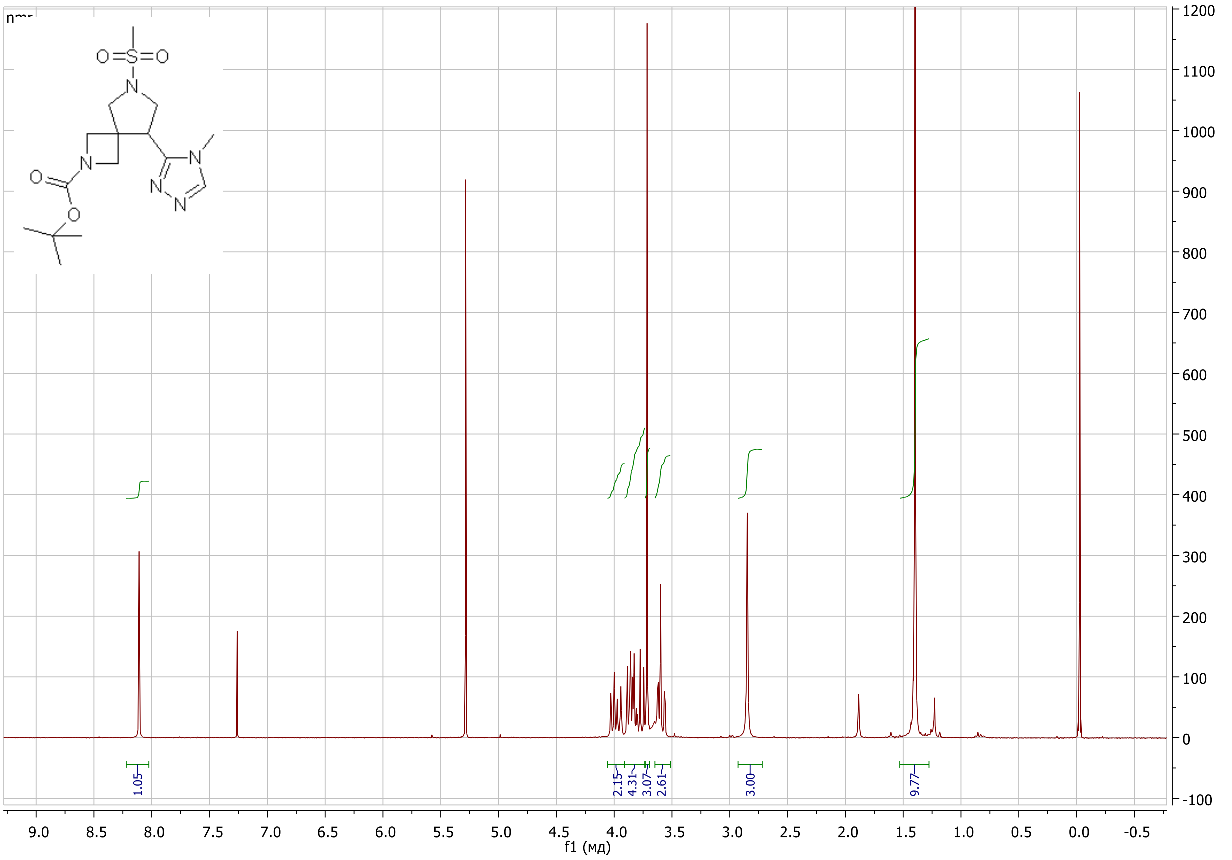

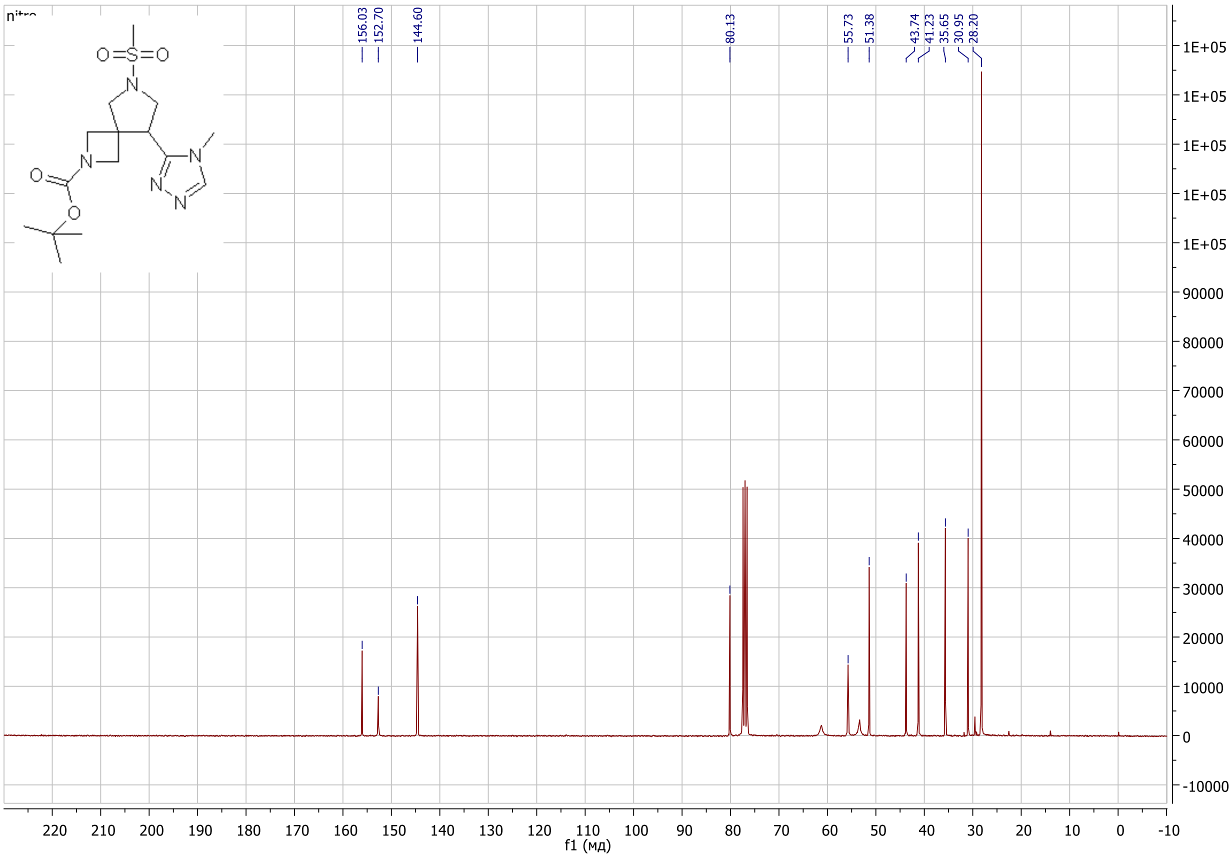

^{1}H and ^{13}C NMR spectra for compound **16**

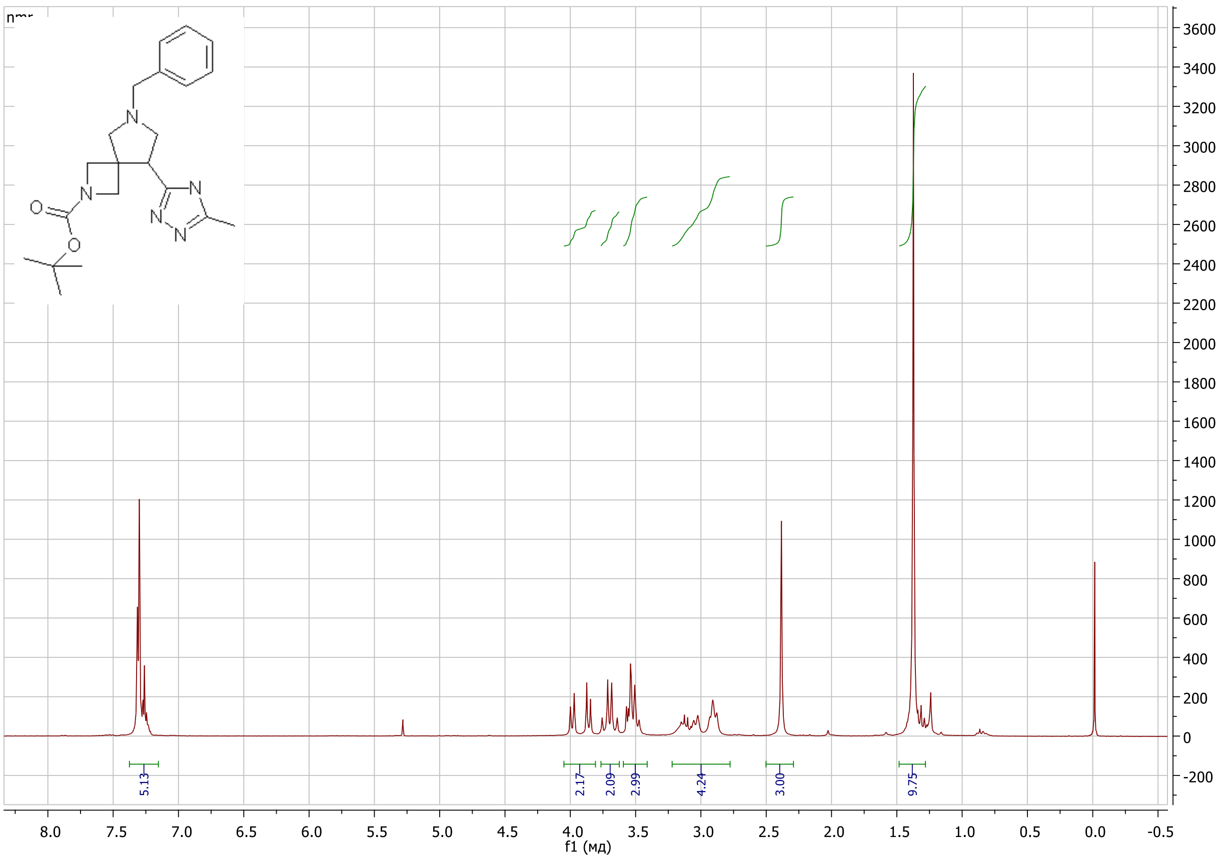

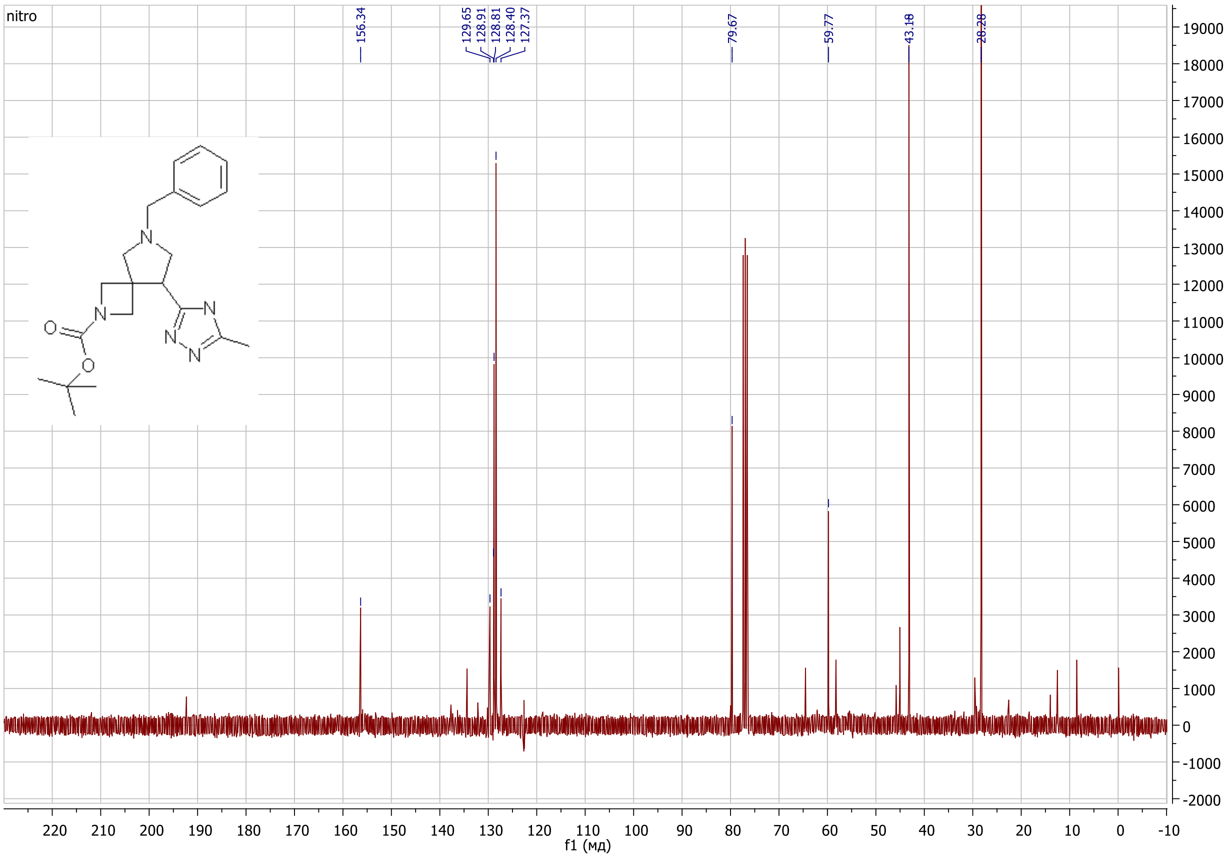

^{1}H and ^{13}C NMR spectra for compound **19**

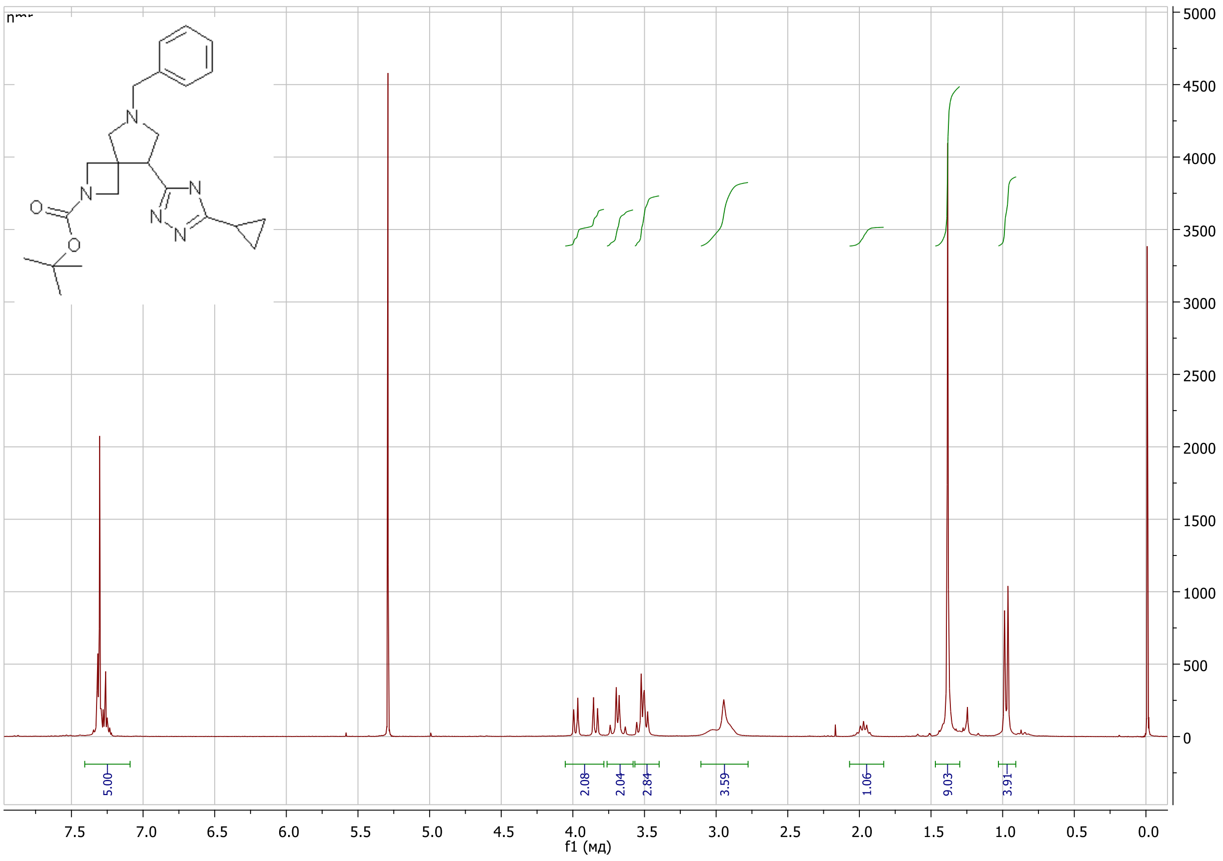

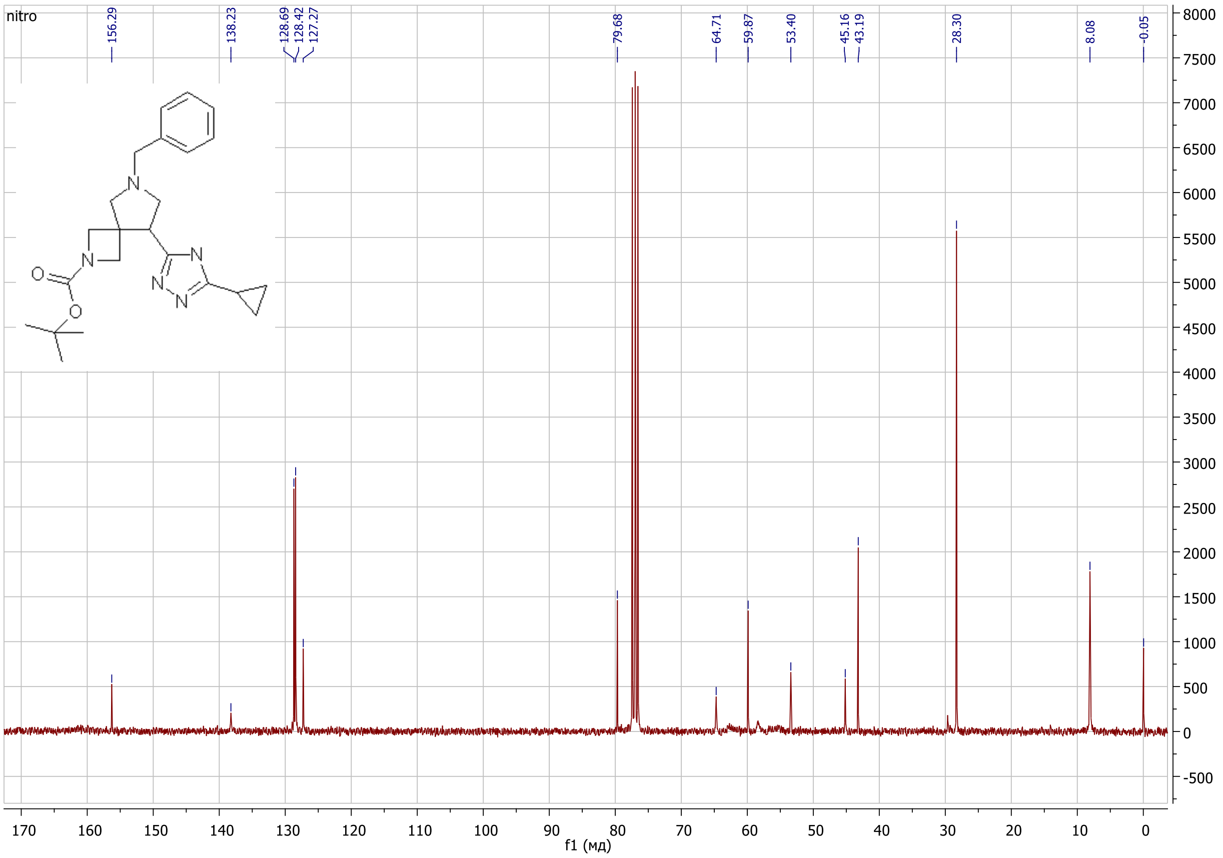

^{1}H and ^{13}C NMR spectra for compound **20**

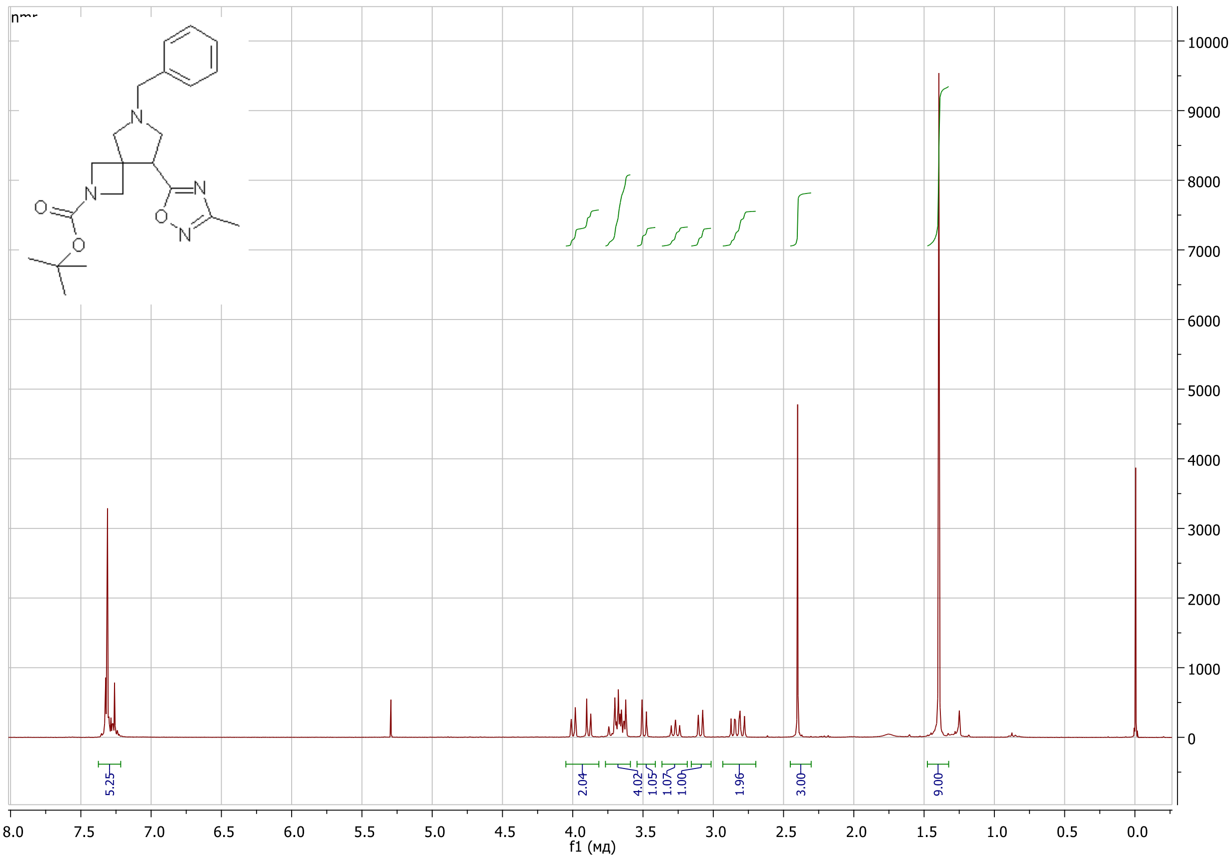

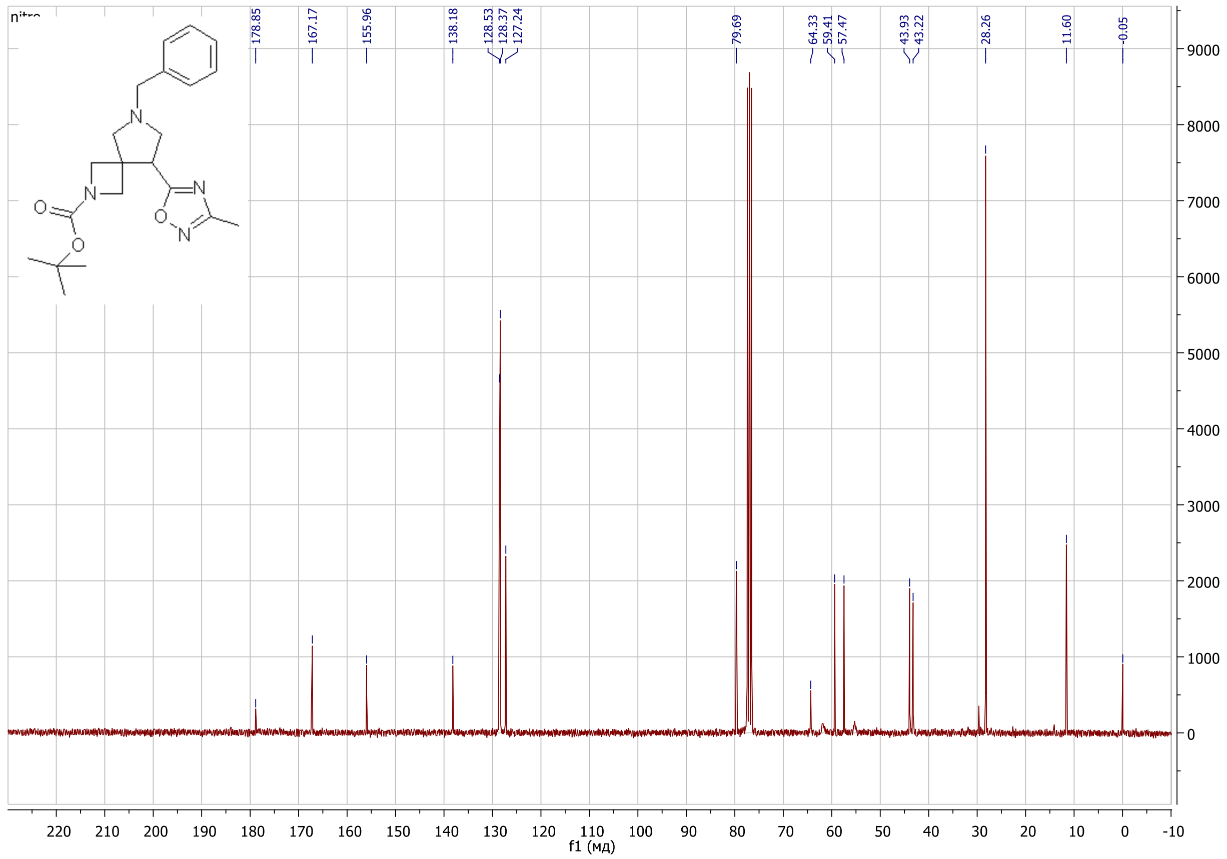

^{1}H and ^{13}C NMR spectra for compound **23**

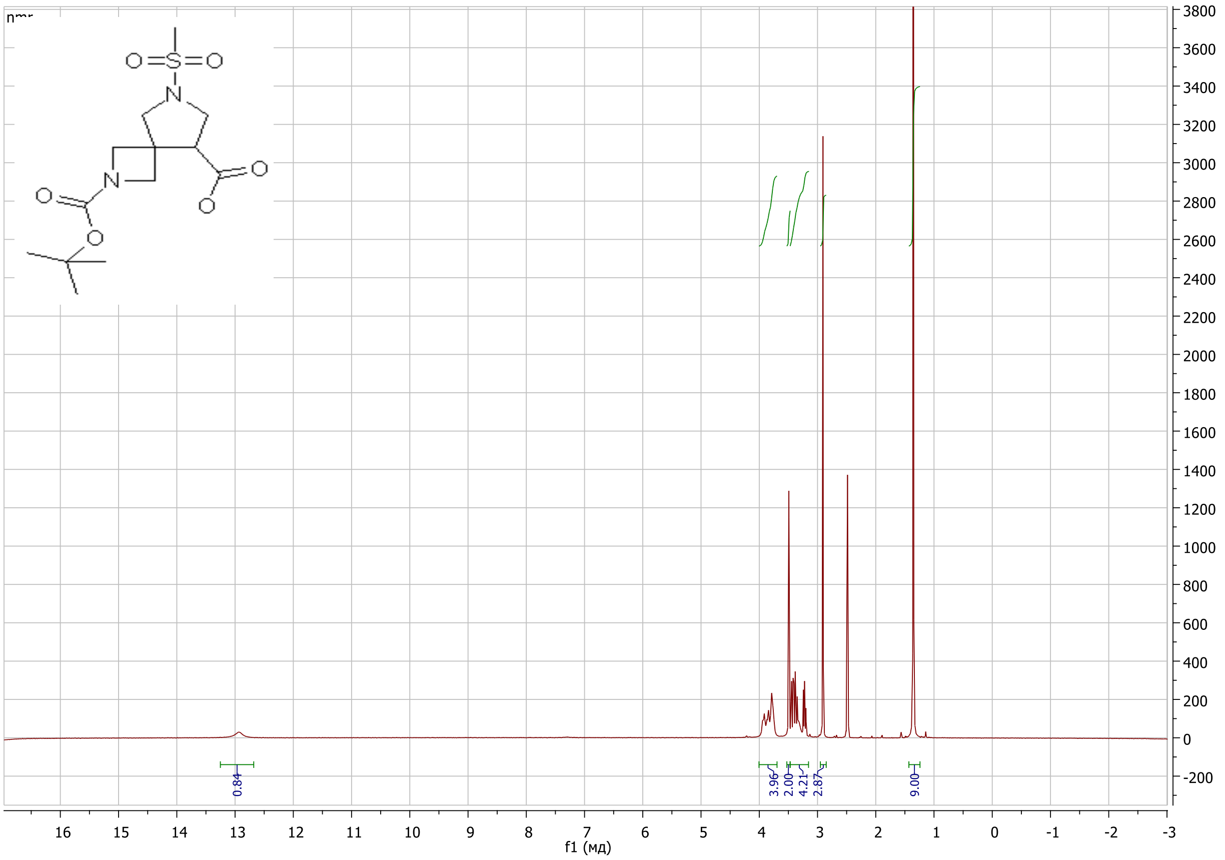

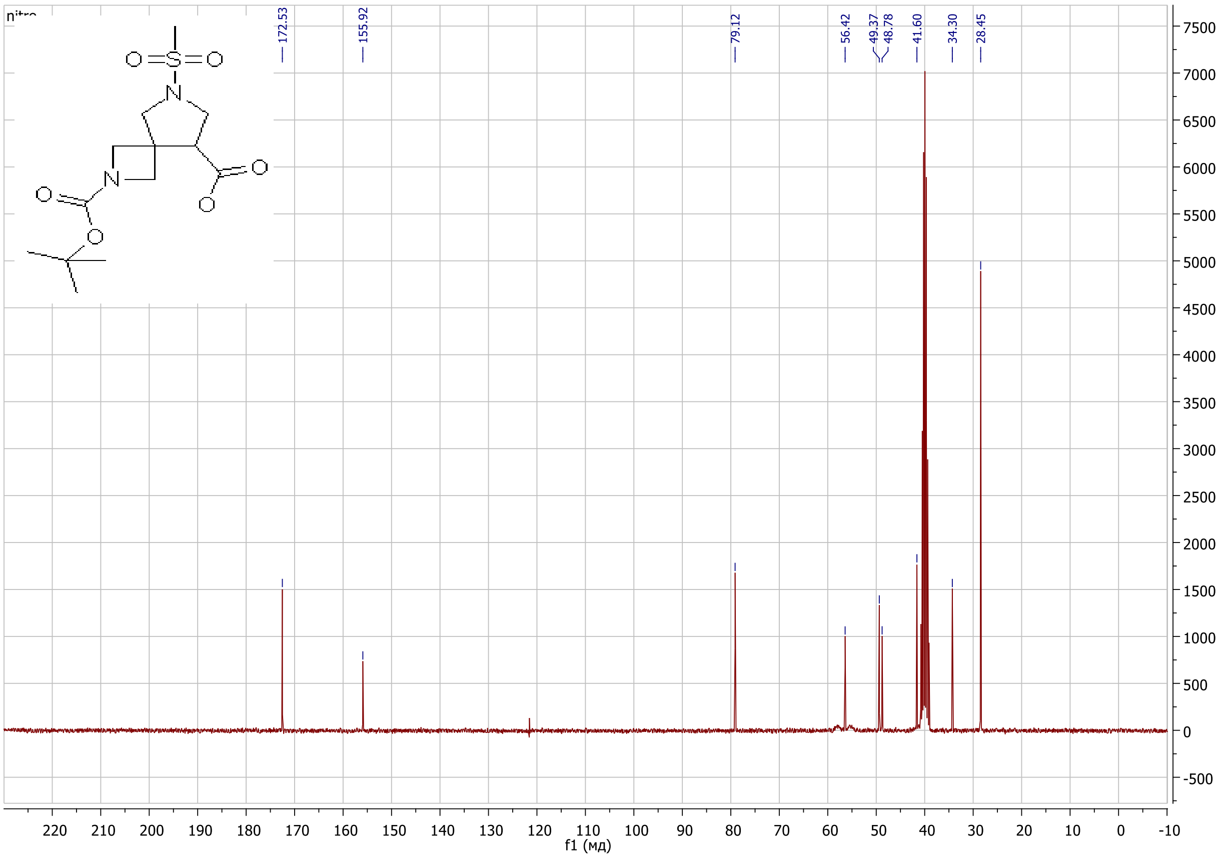

^{1}H and ^{13}C NMR spectra for compound **25**

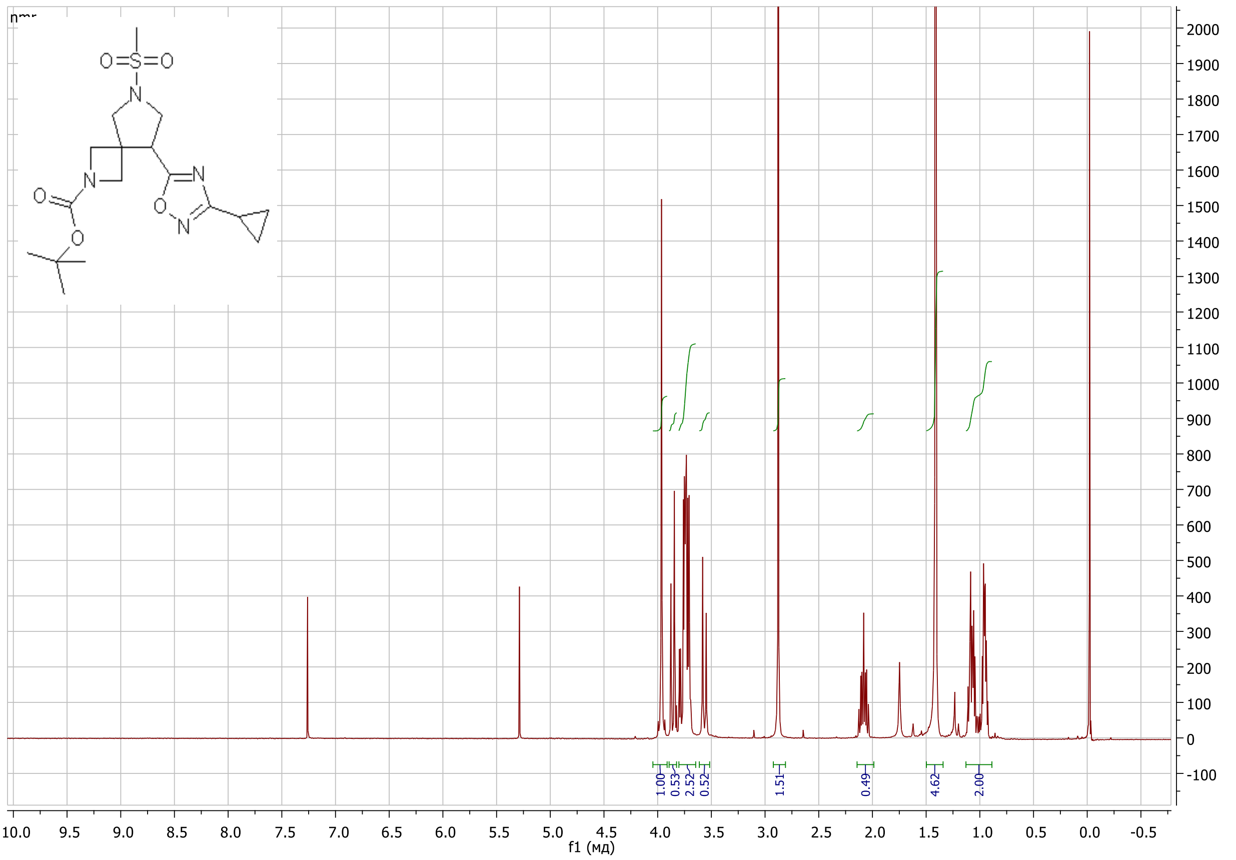

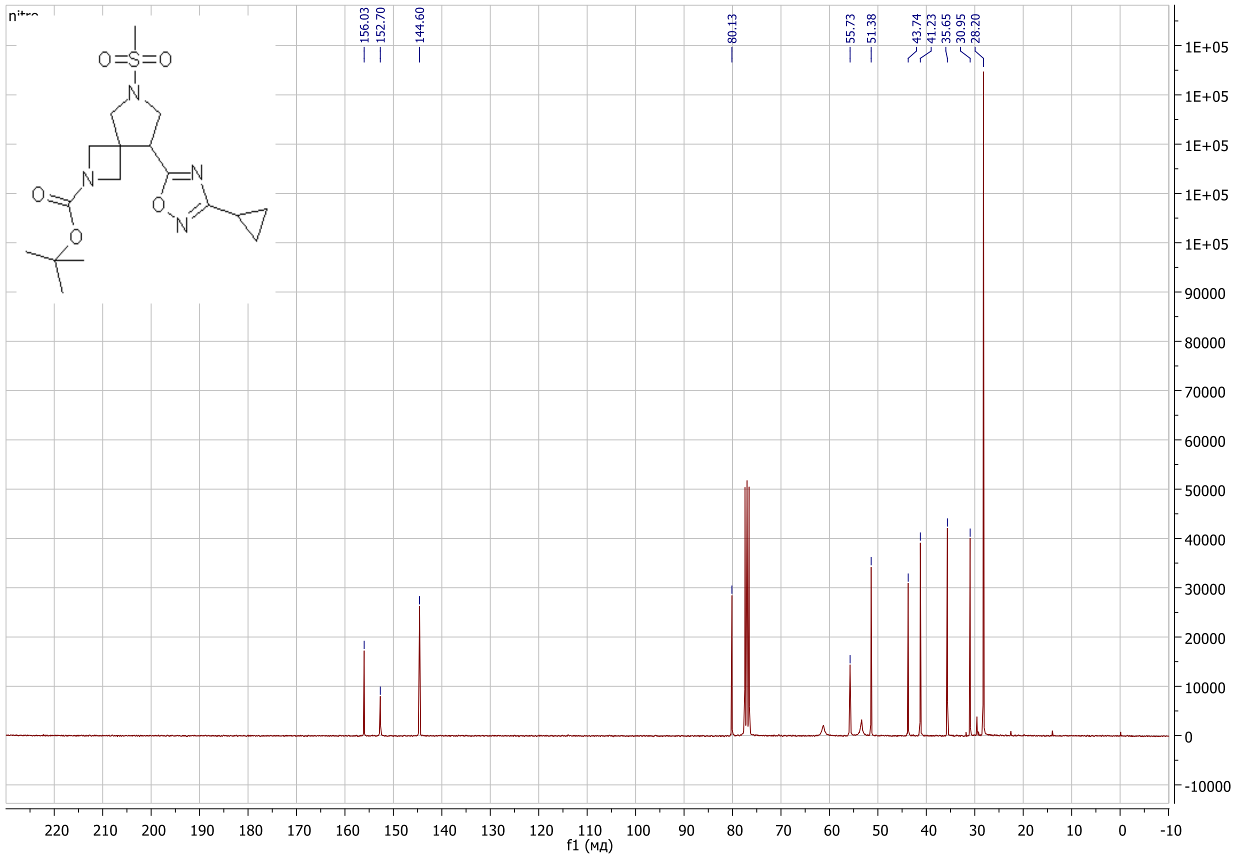

^{1}H and ^{13}C NMR spectra for compound **26**

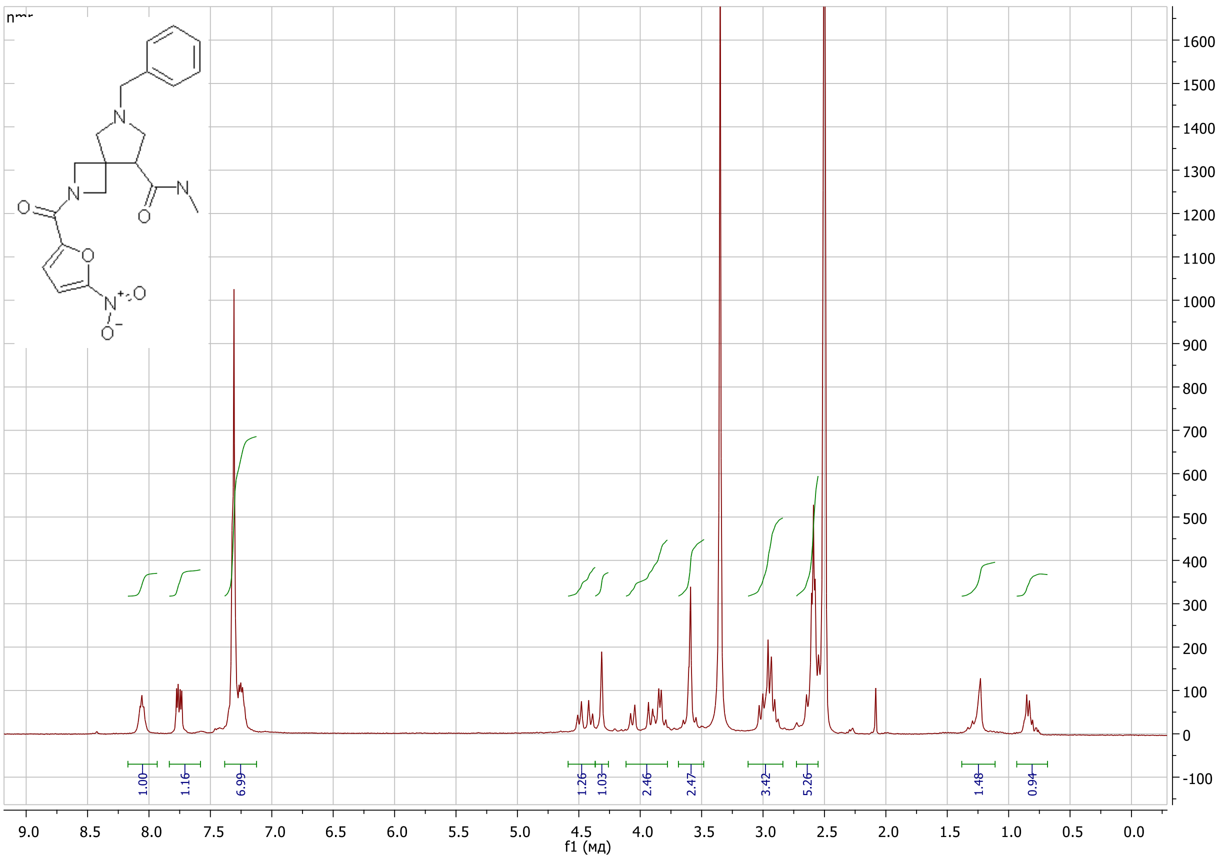

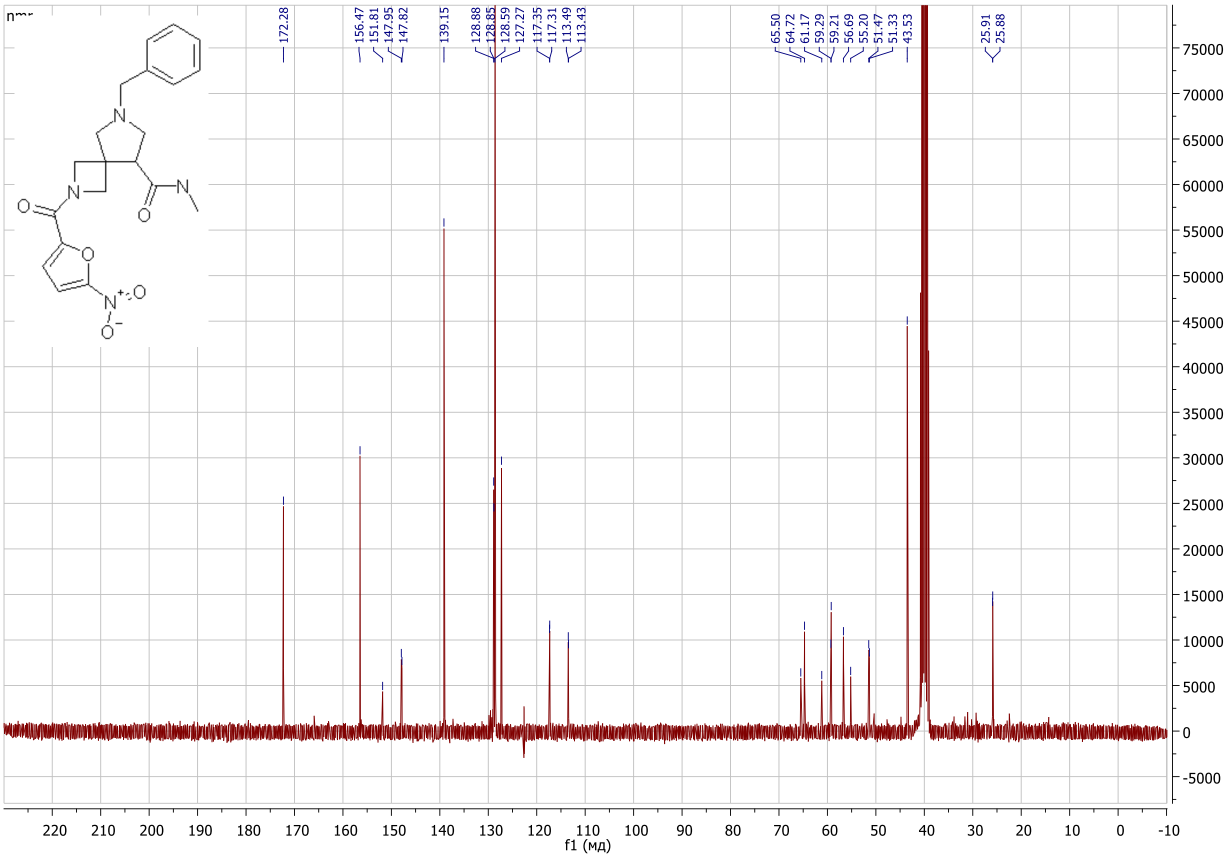

^{1}H and ^{13}C NMR spectra for compound **5a**

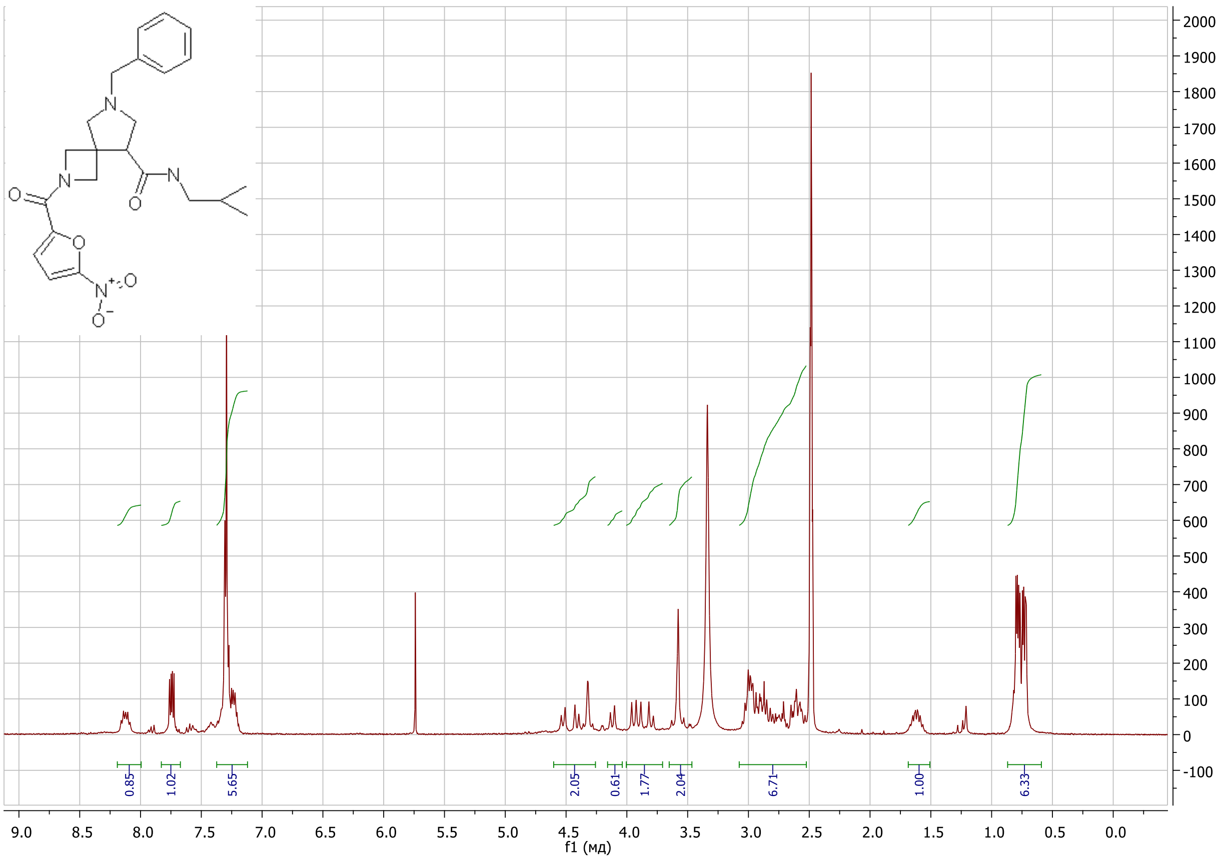

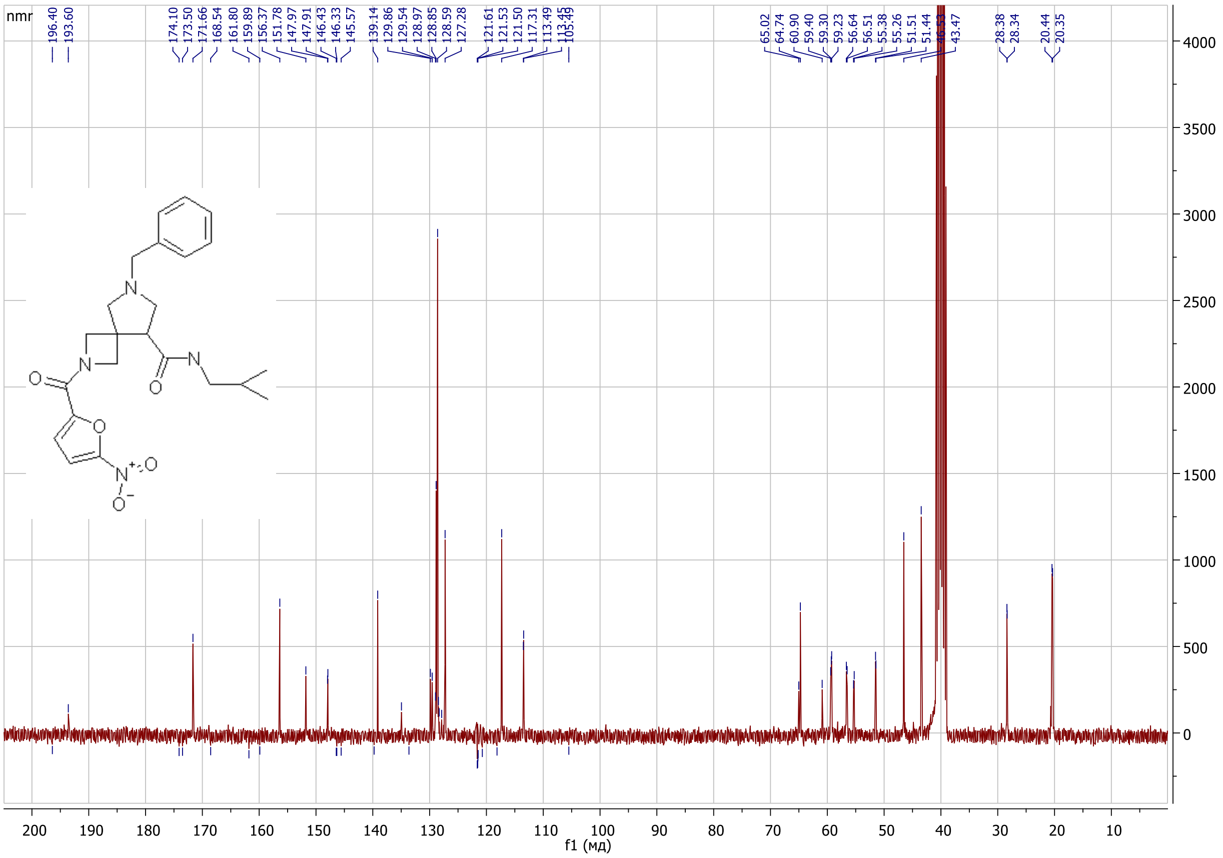

^{1}H and ^{13}C NMR spectra for compound **5b**

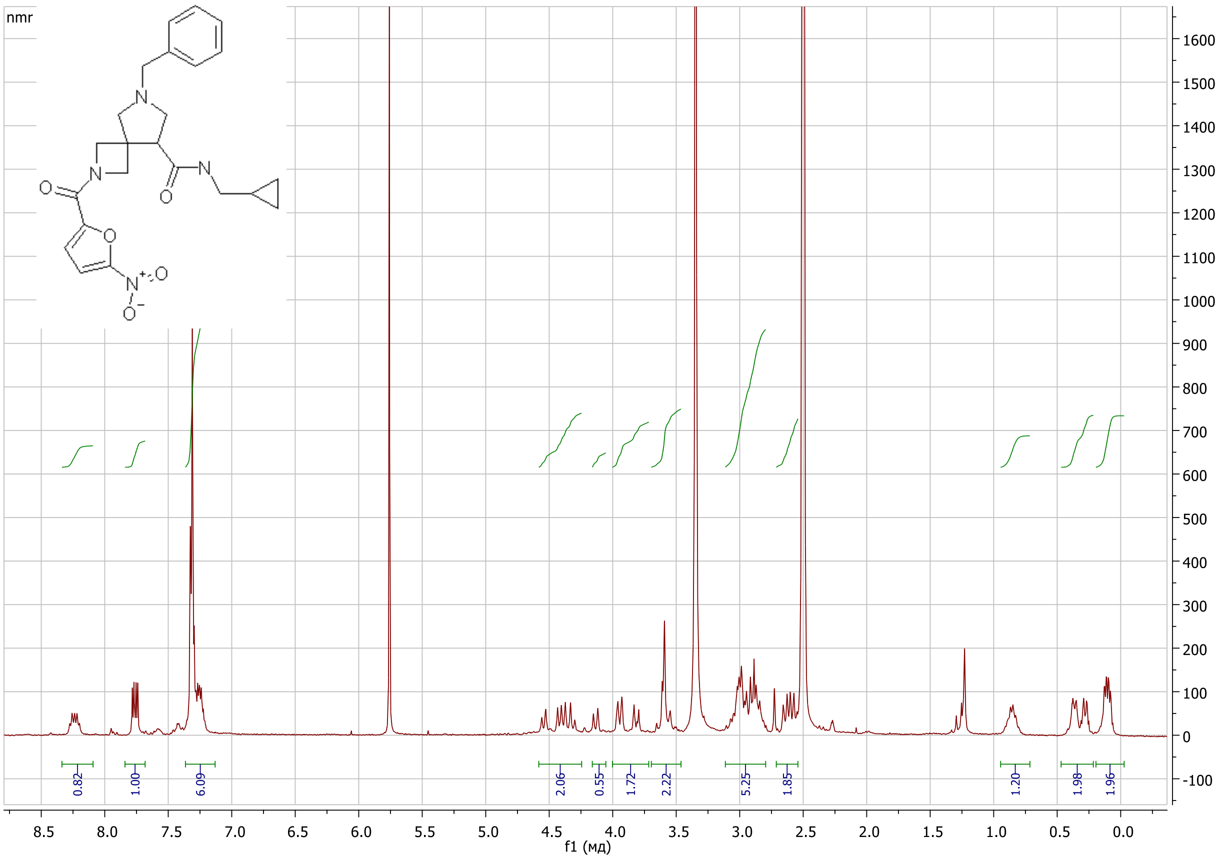

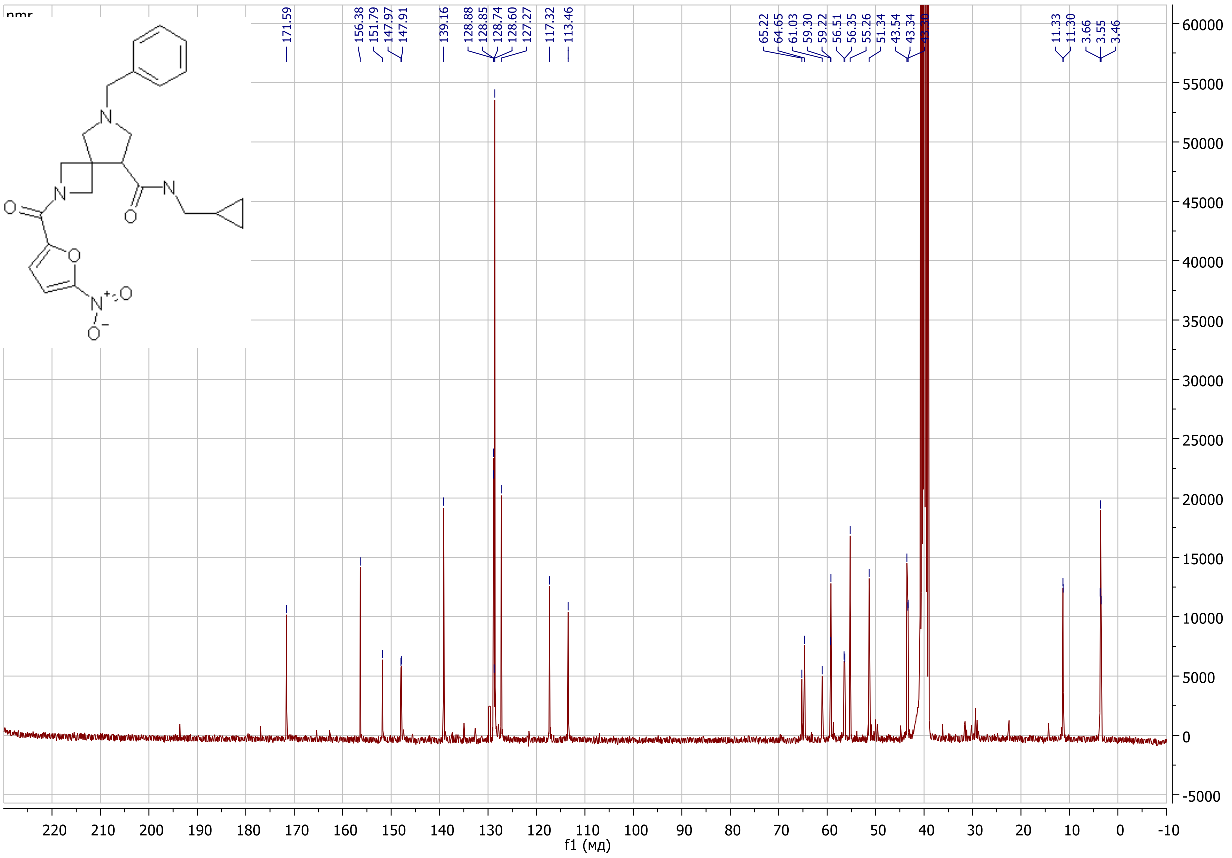

^{1}H and ^{13}C NMR spectra for compound **5c**

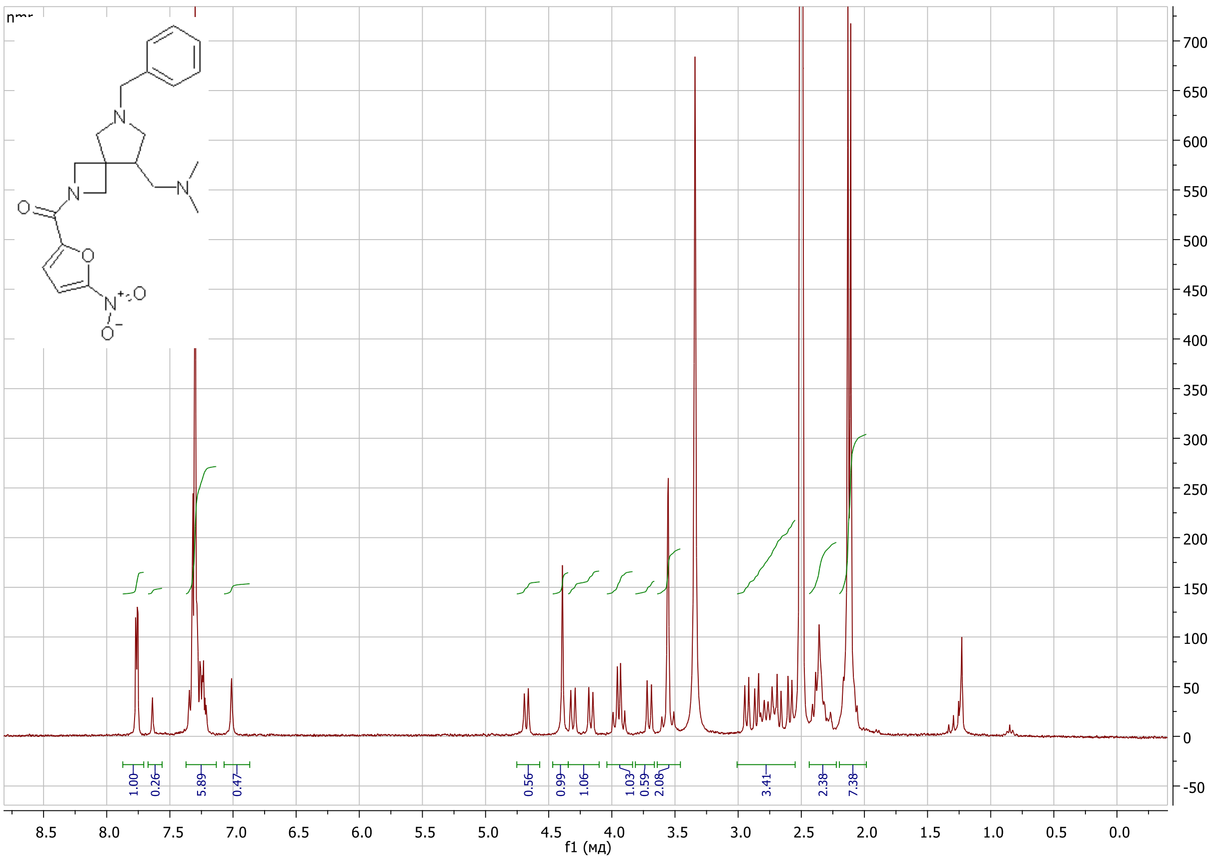

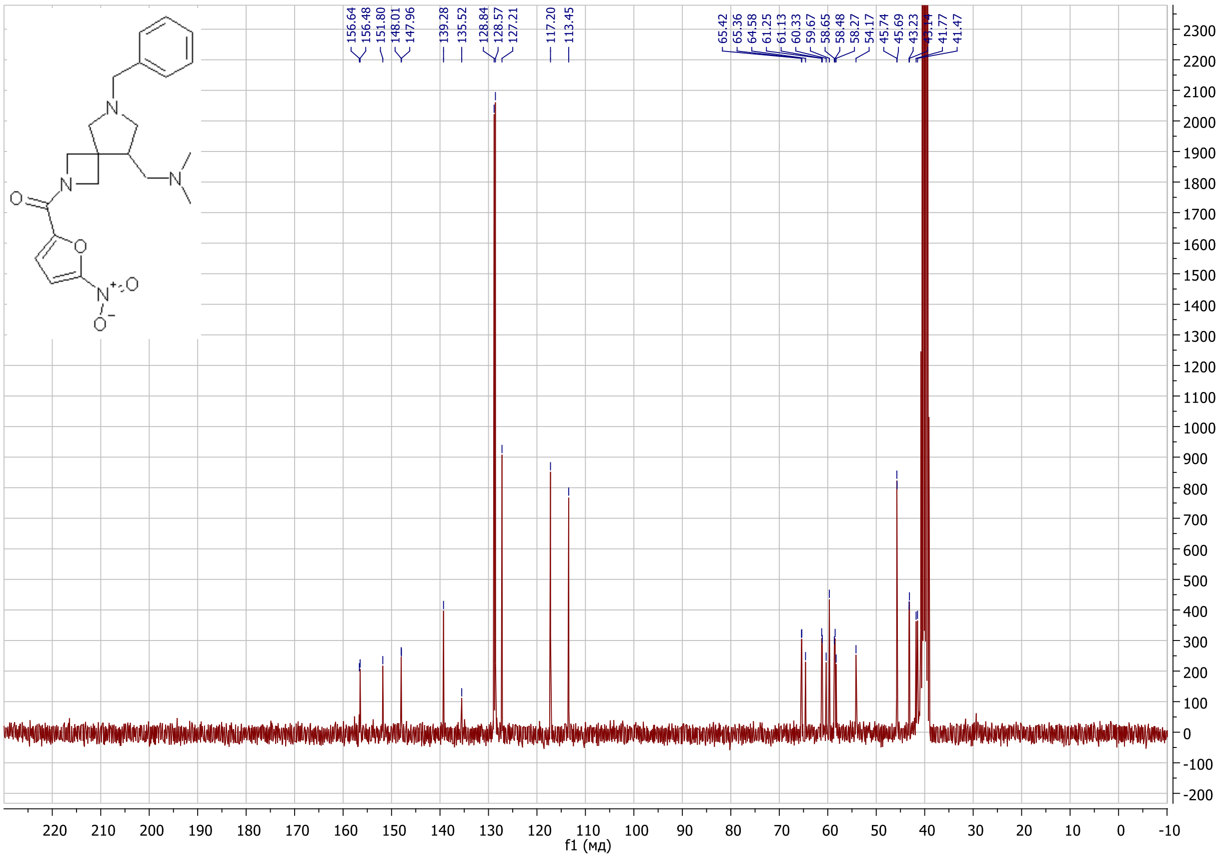

^{1}H and ^{13}C NMR spectra for compound **6d**

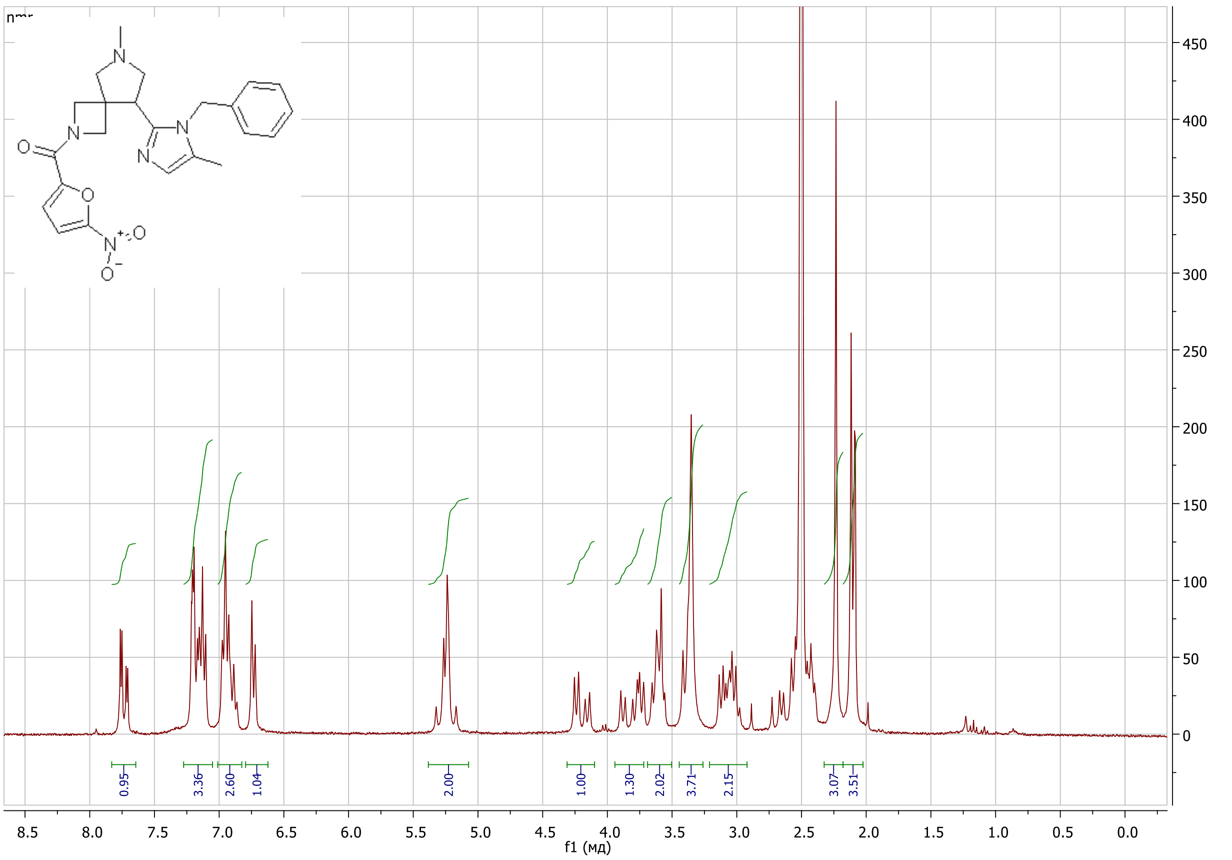

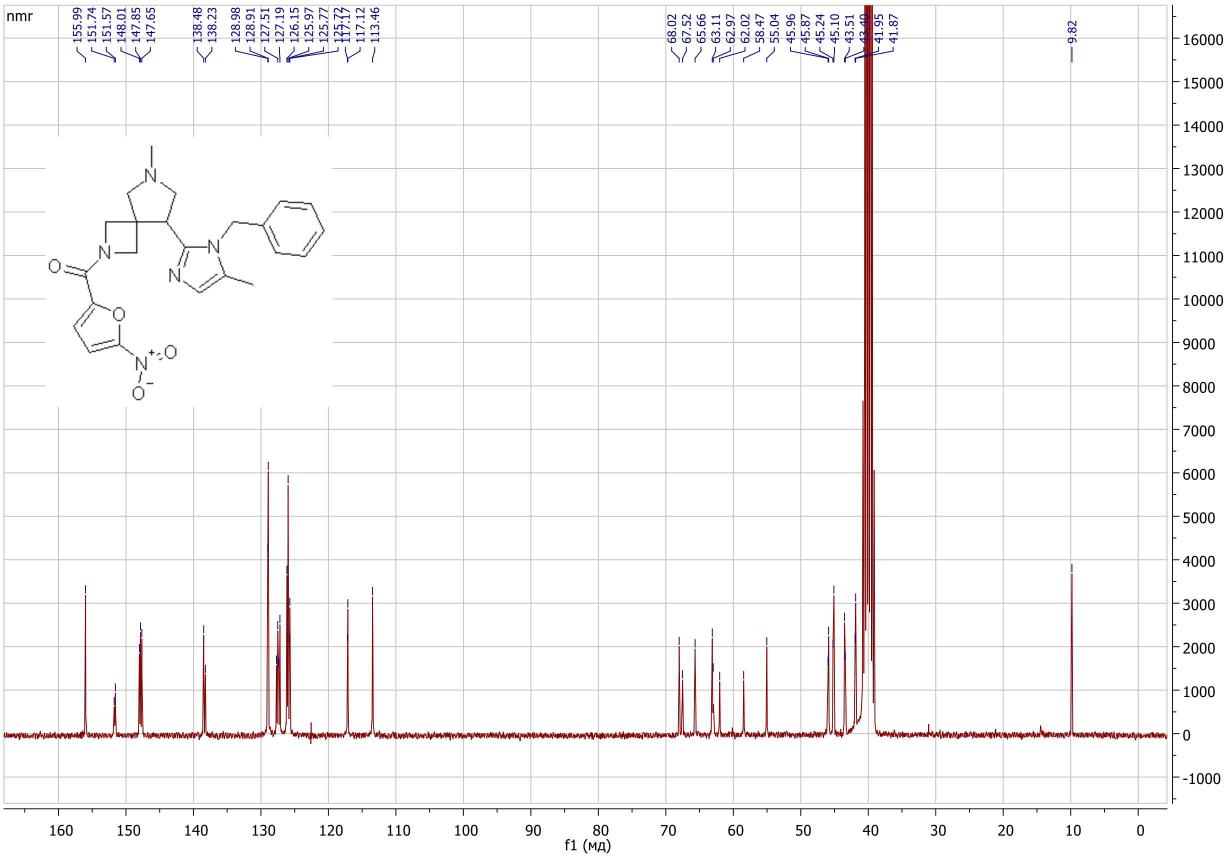

^{1}H and ^{13}C NMR spectra for compound **12**

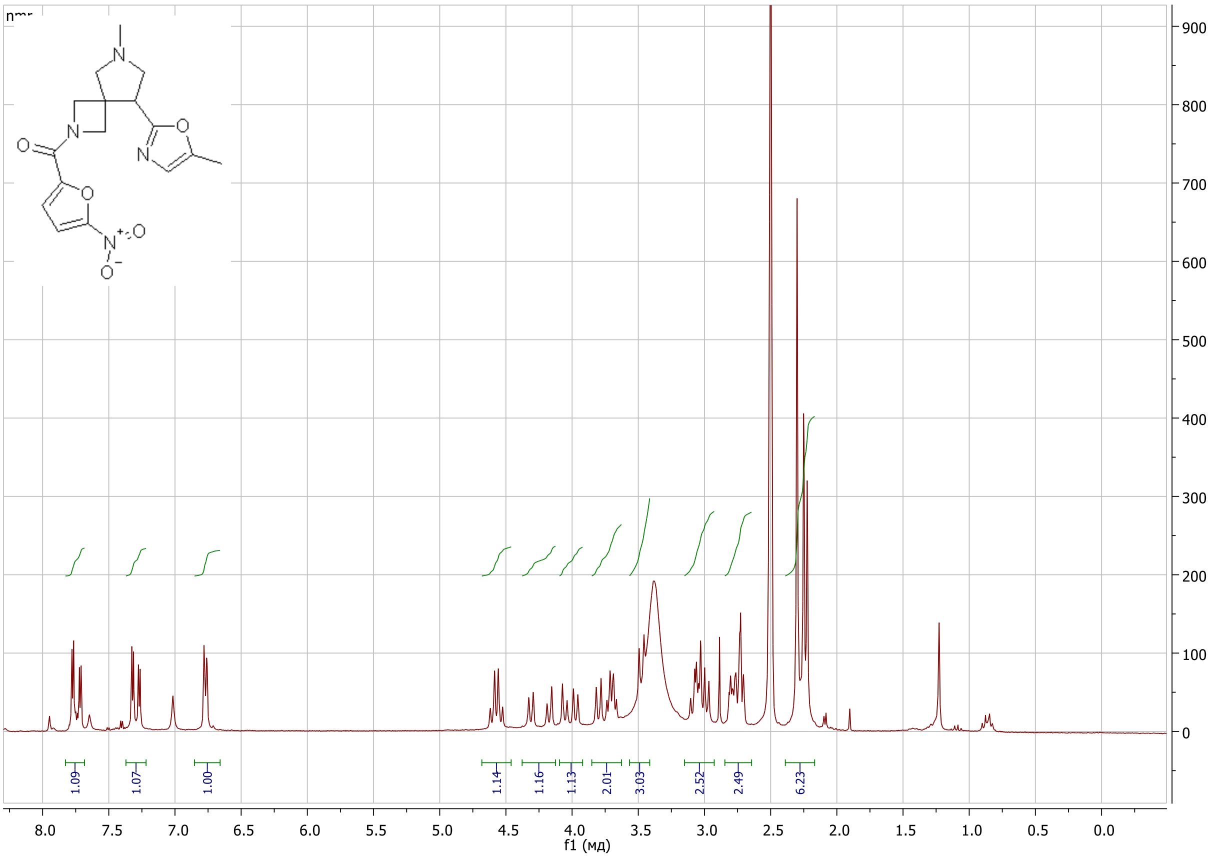

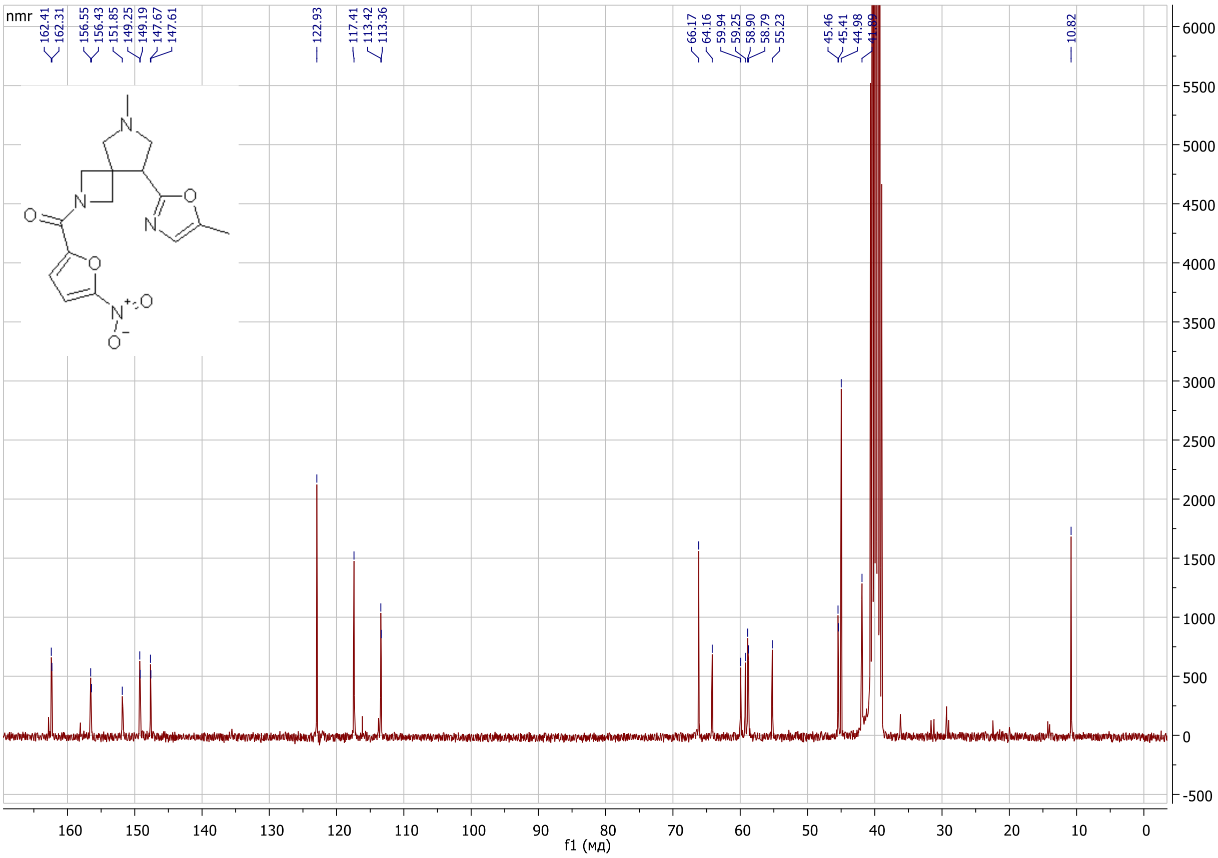

^{1}H and ^{13}C NMR spectra for compound **13**

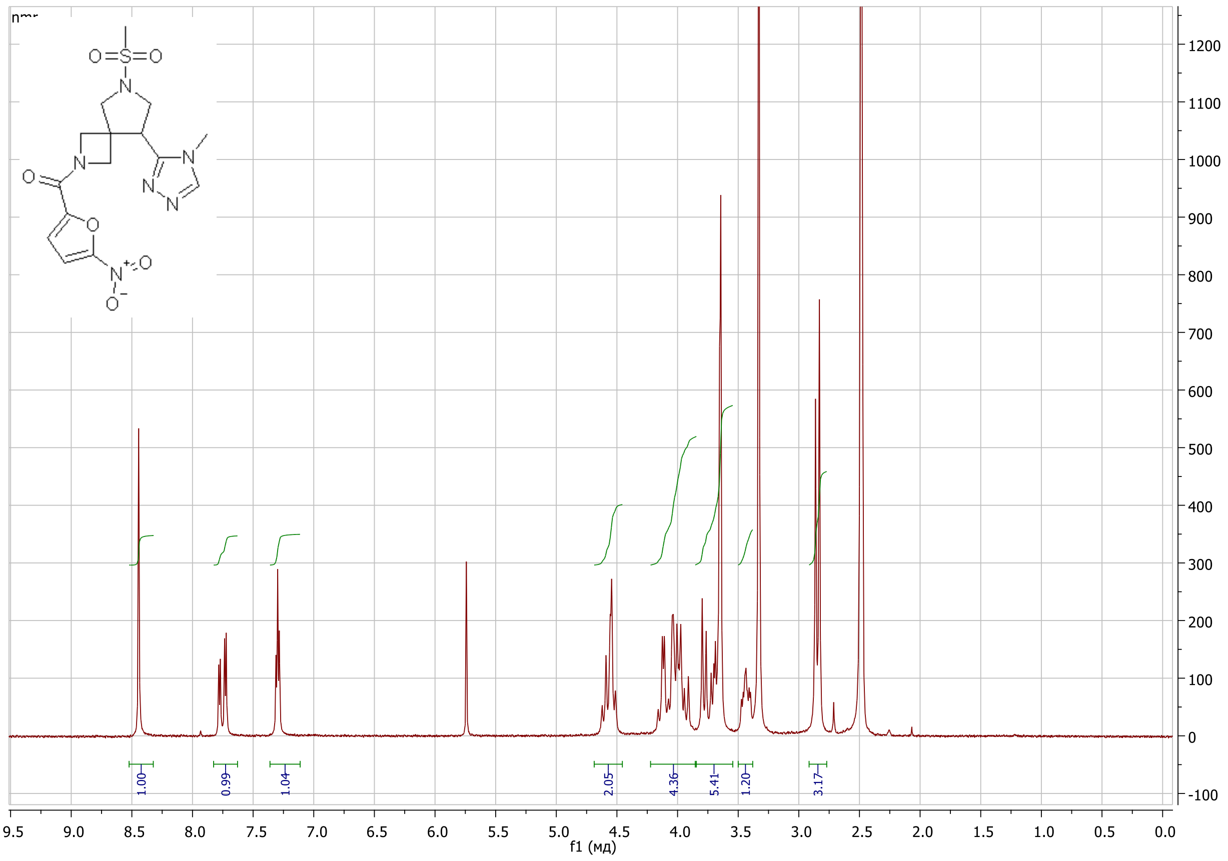

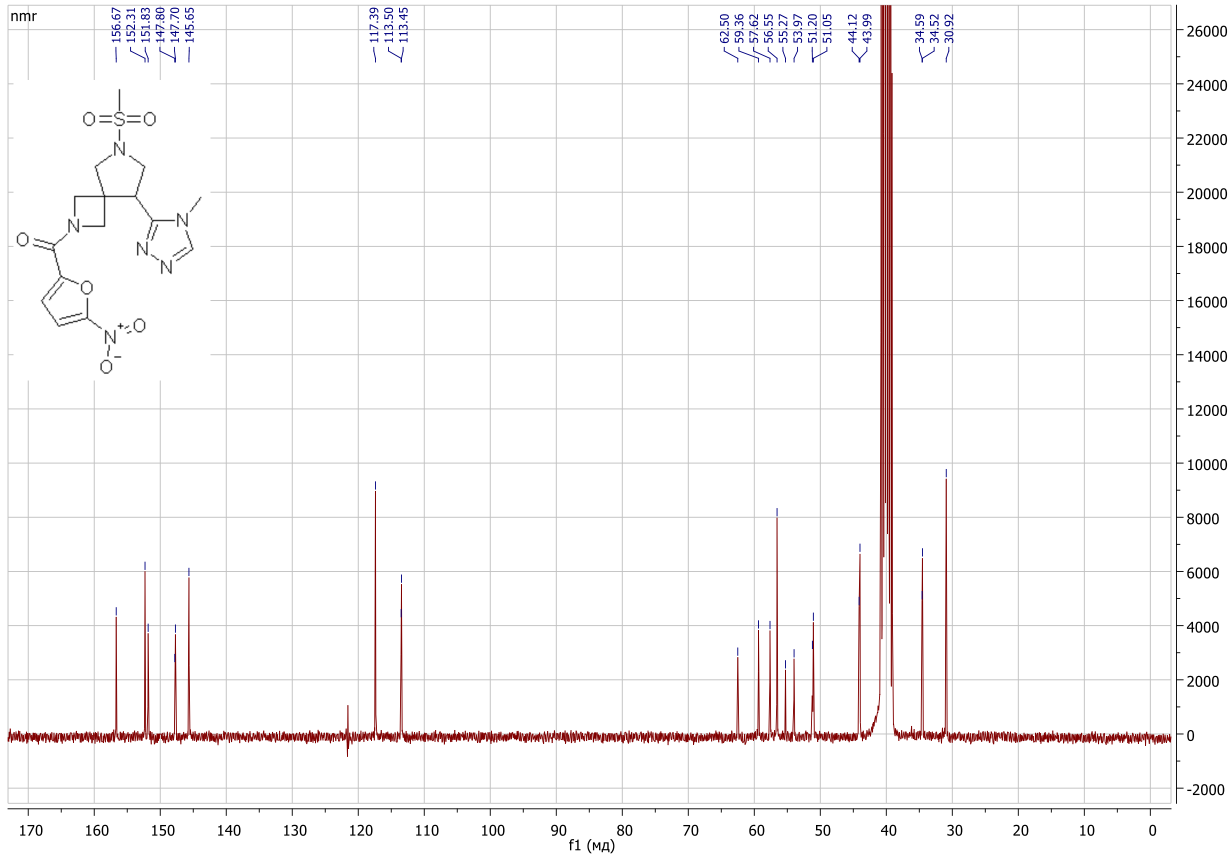

^{1}H and ^{13}C NMR spectra for compound **17**

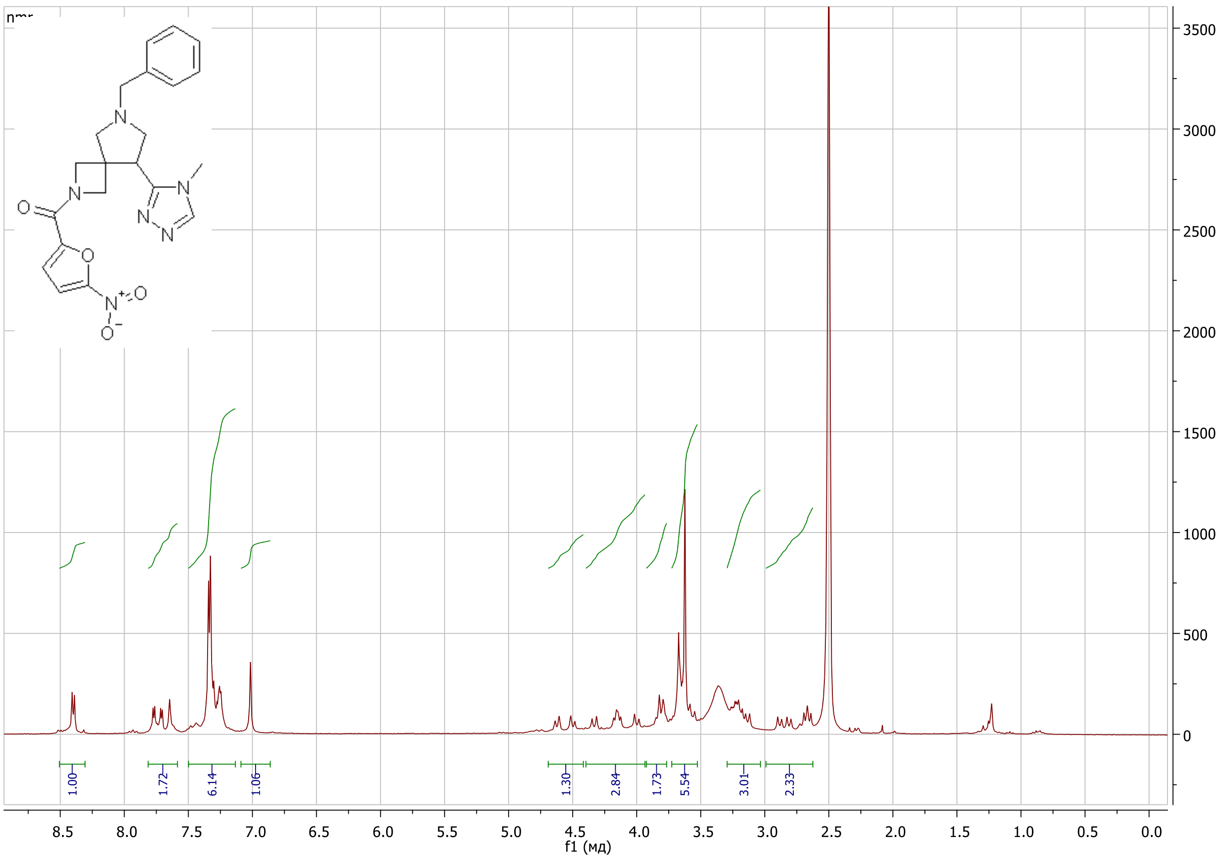

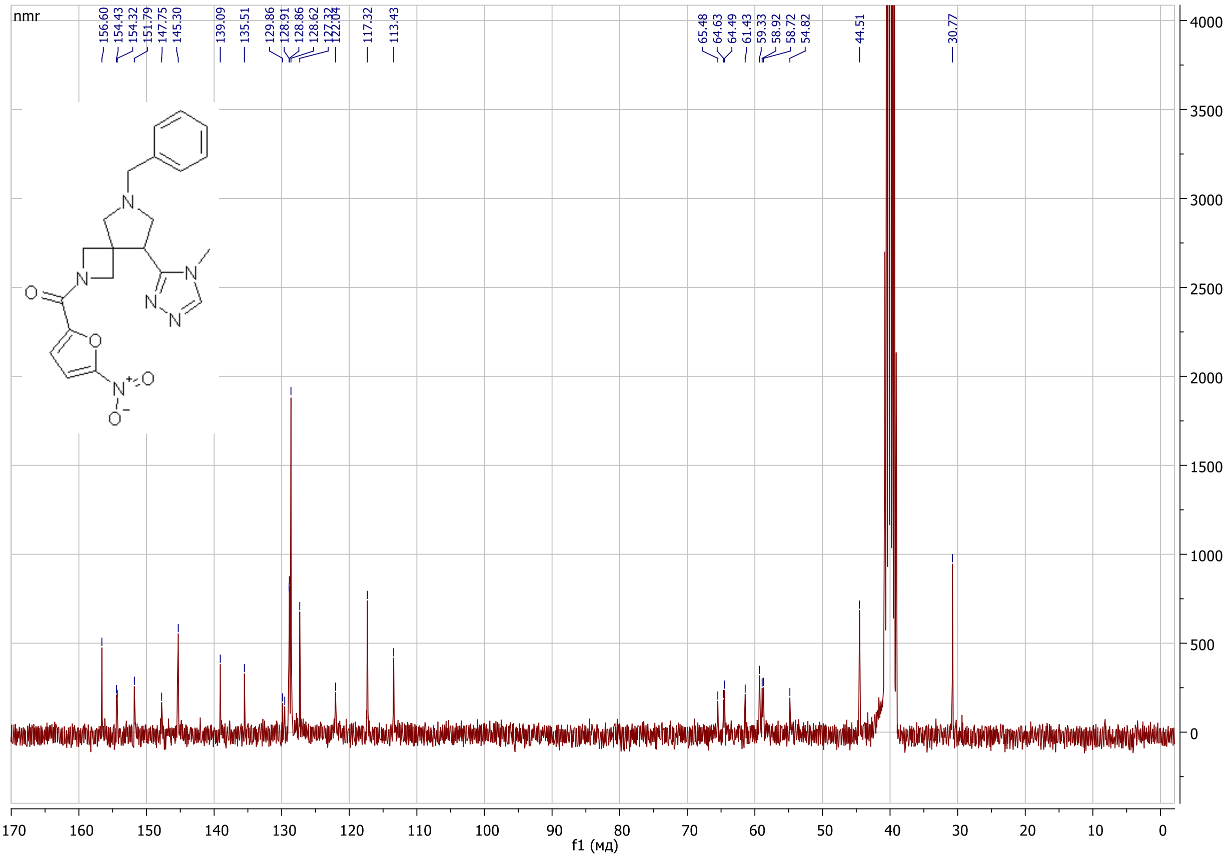

^{1}H and ^{13}C NMR spectra for compound **18**

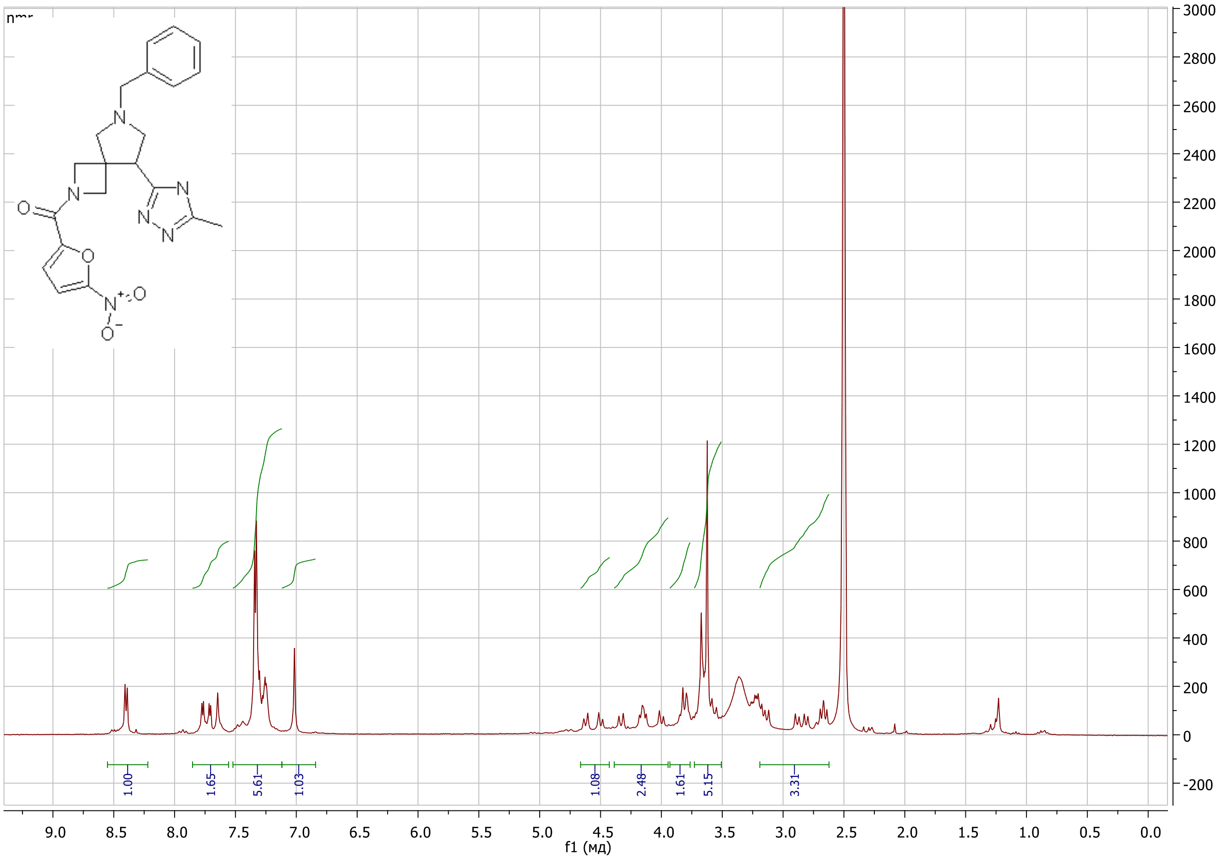

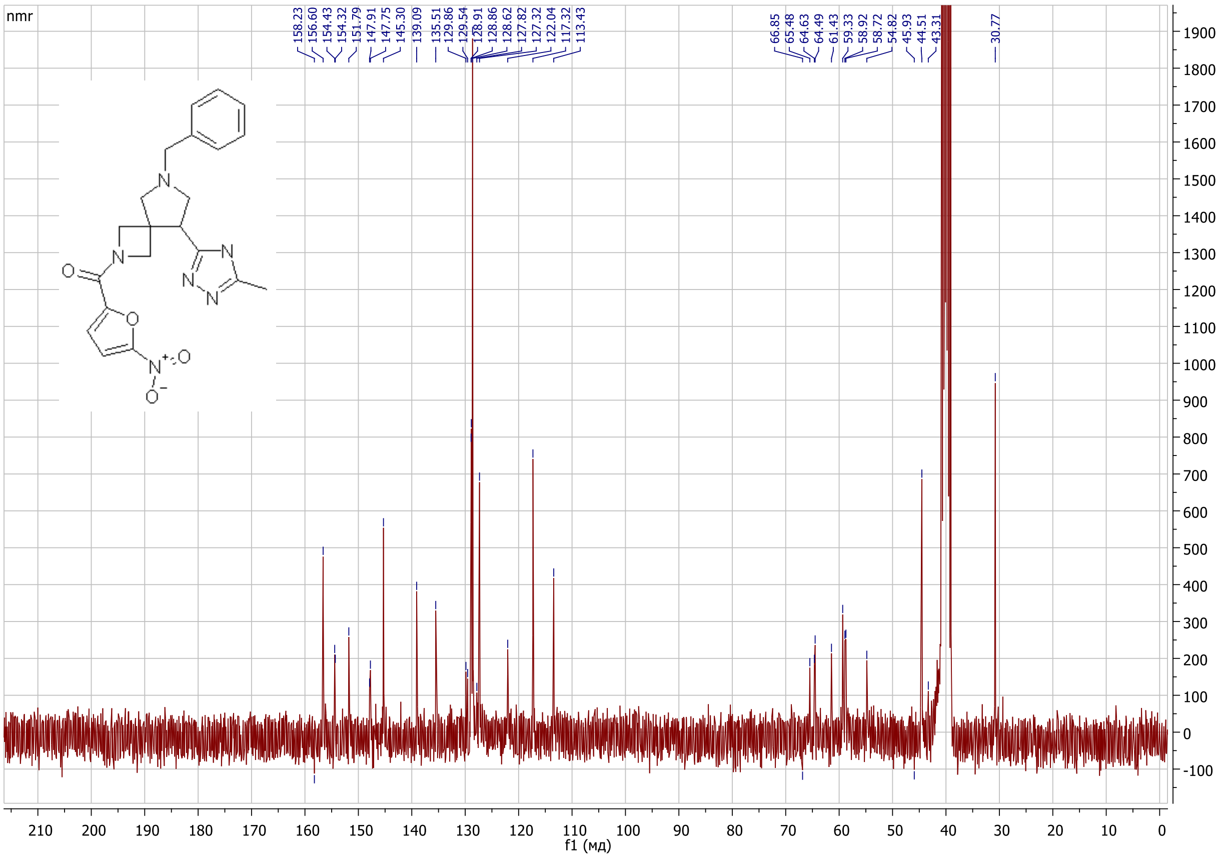

^{1}H and ^{13}C NMR spectra for compound **21**

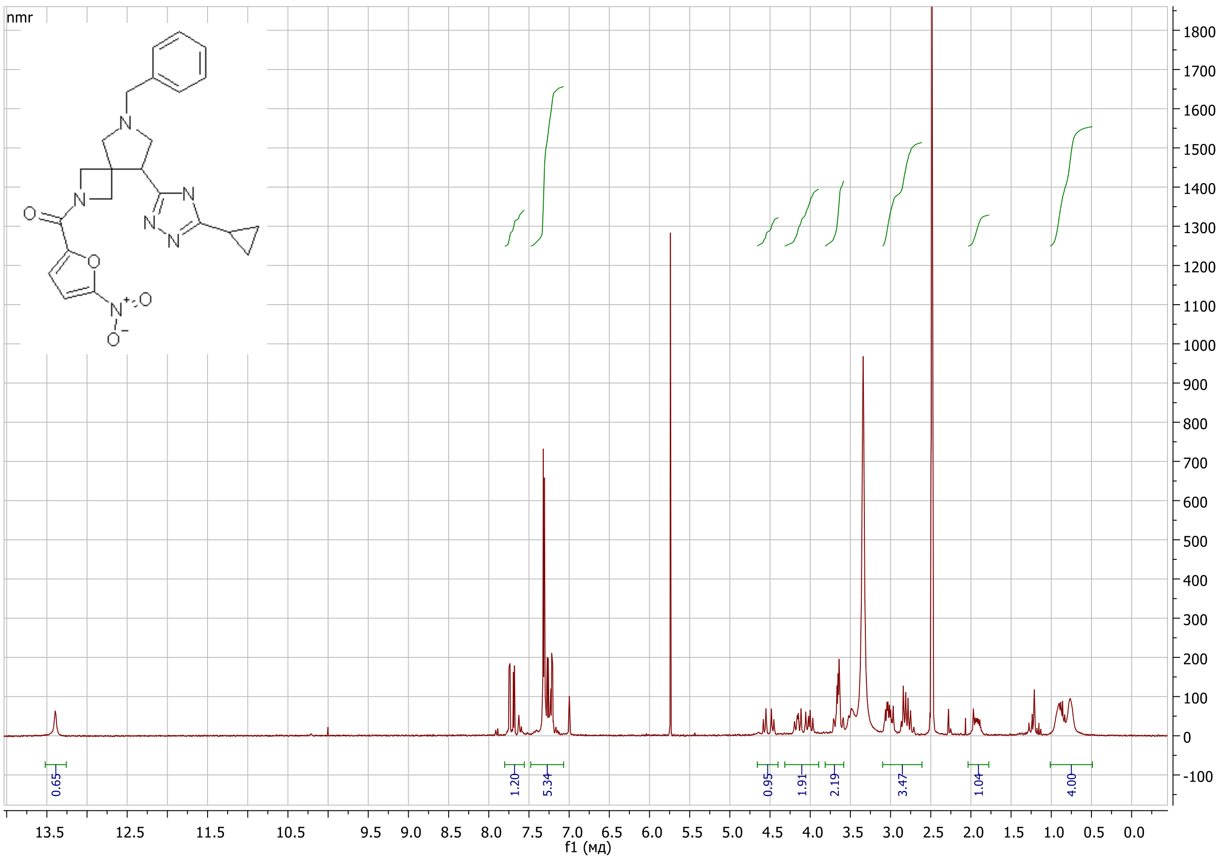

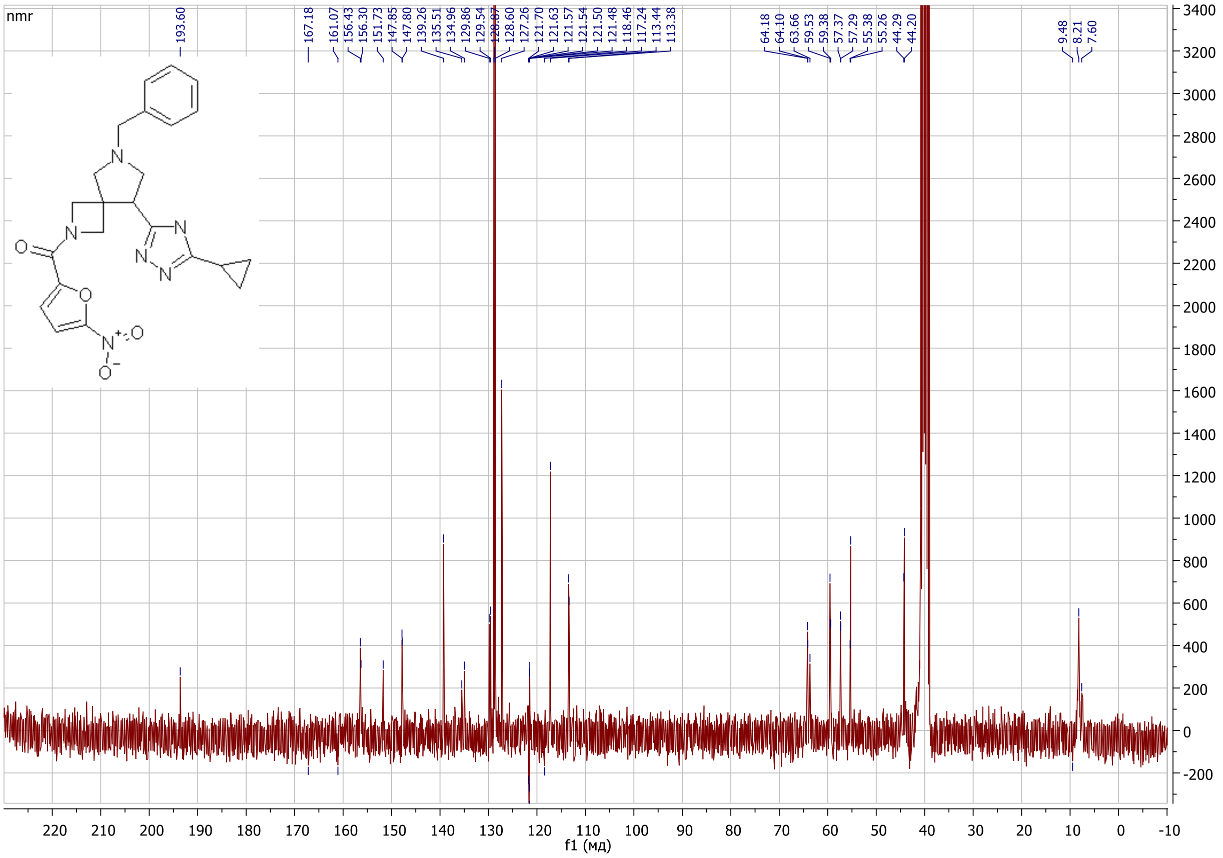

^{1}H and ^{13}C NMR spectra for compound **22**

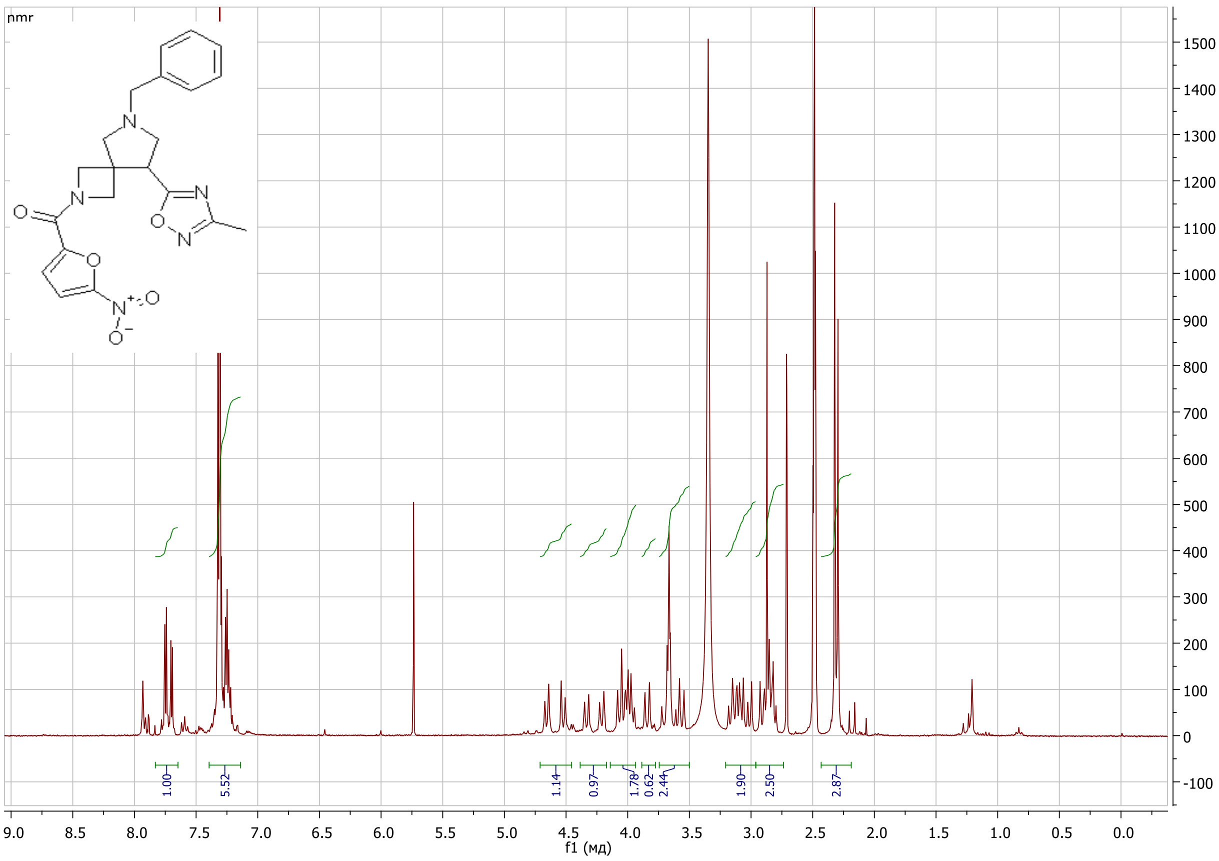

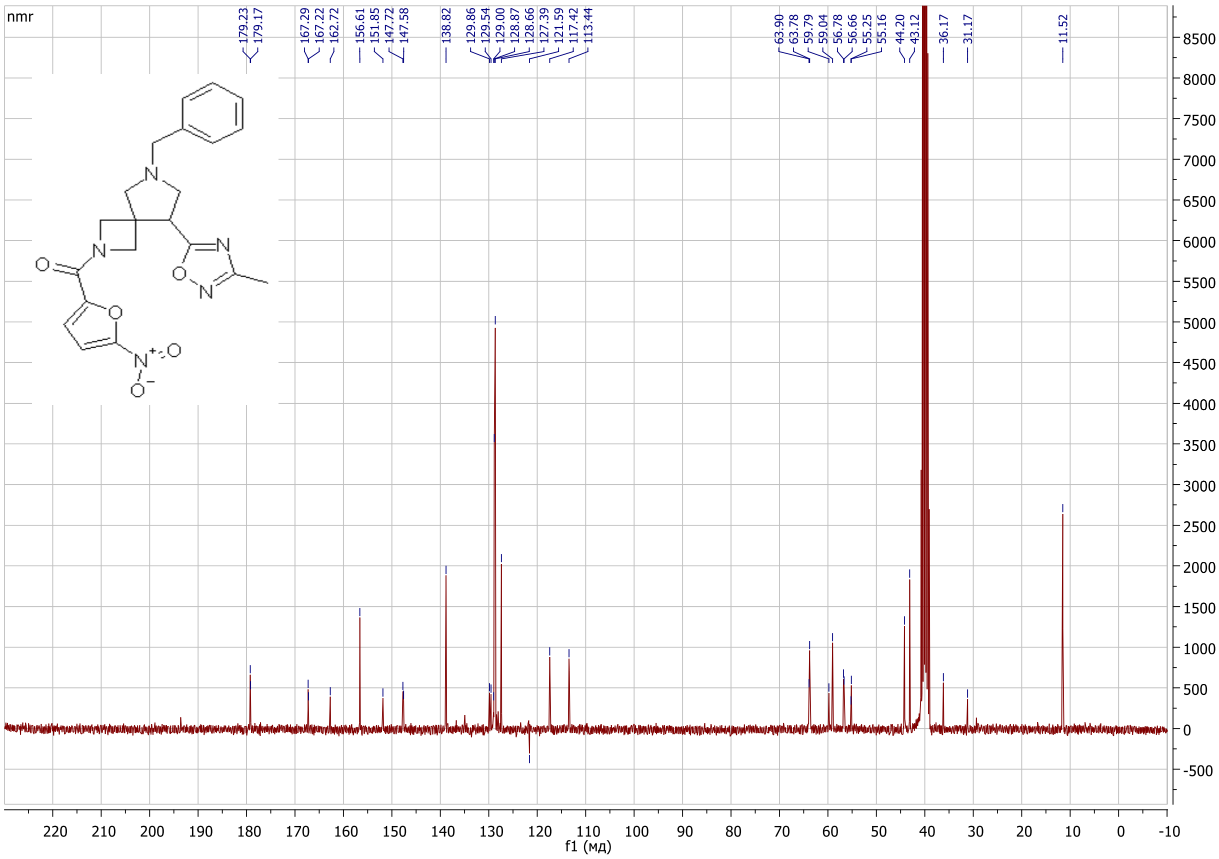

^{1}H and ^{13}C NMR spectra for compound **24**

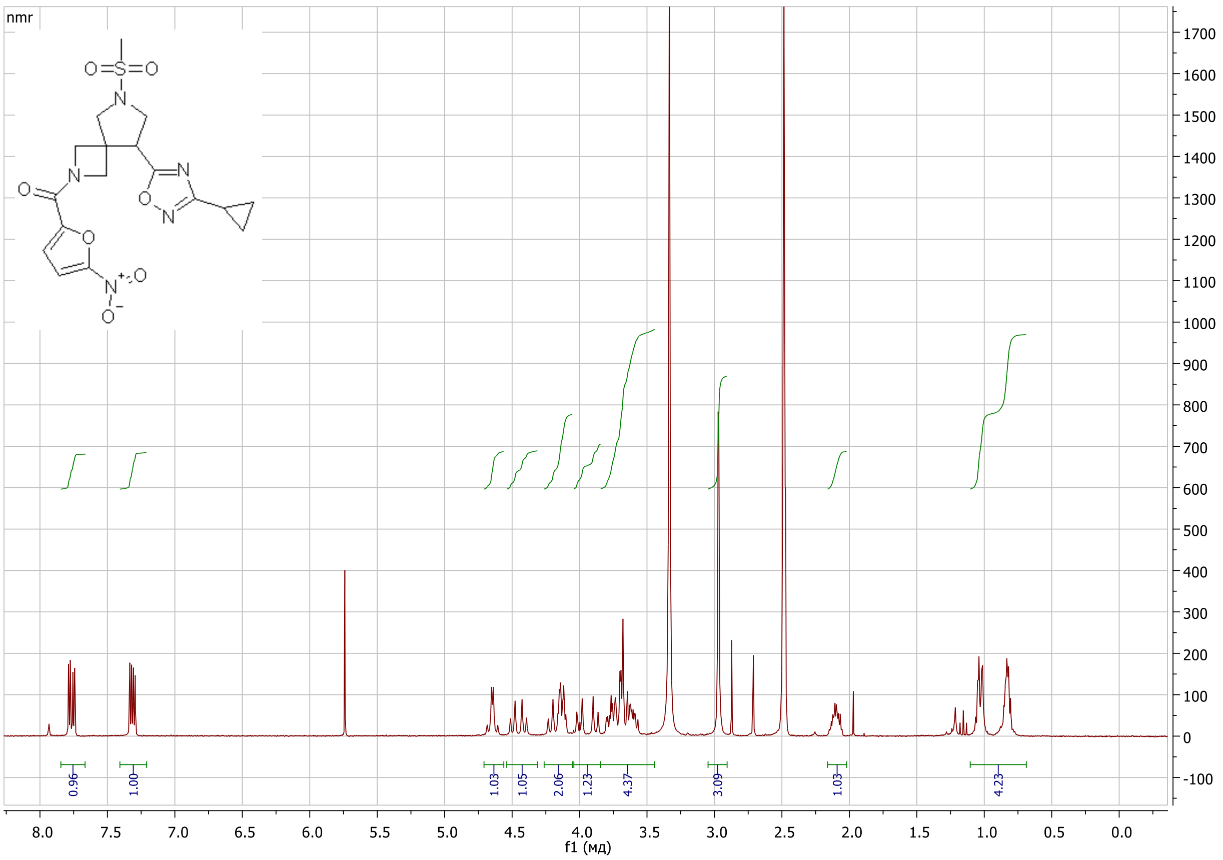

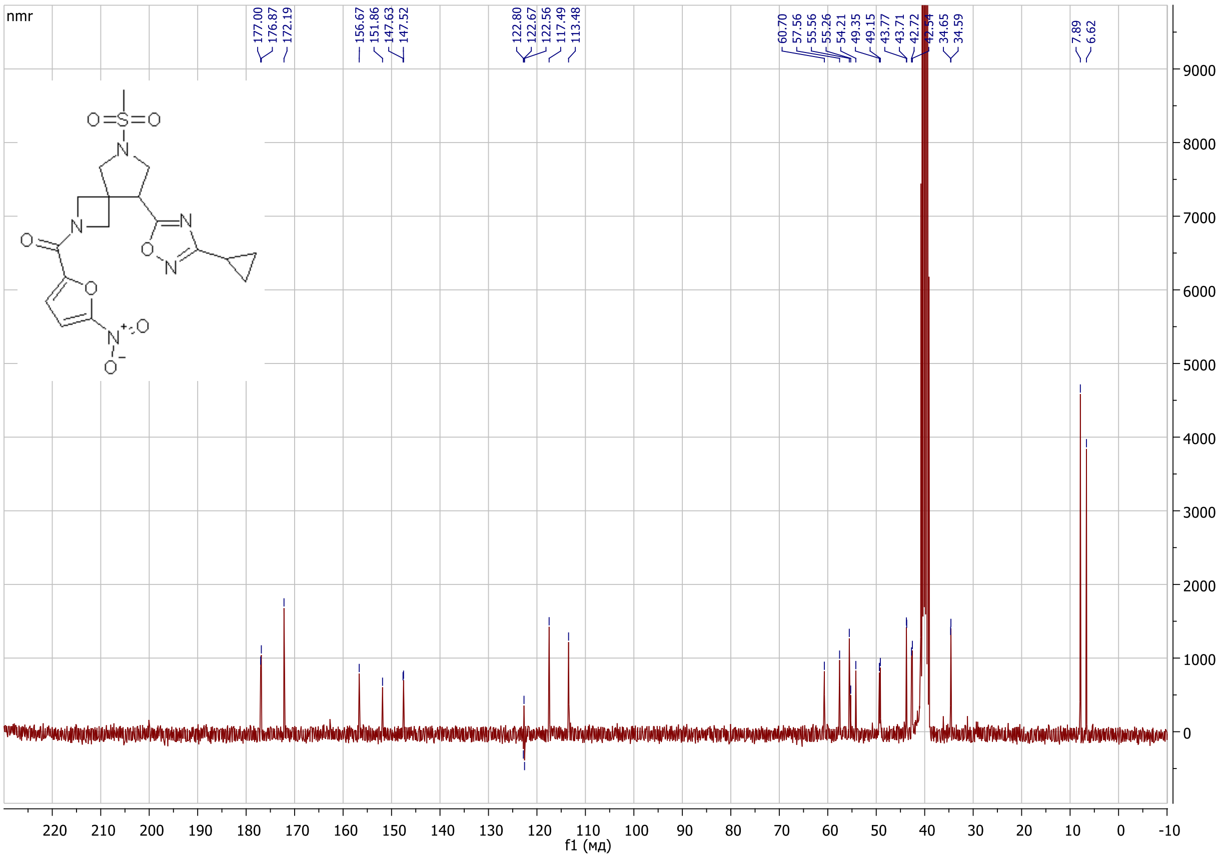

^{1}H and ^{13}C NMR spectra for compound **27**

Images of exemplary plates

Compound **21** (MIC 50 μg/mL)

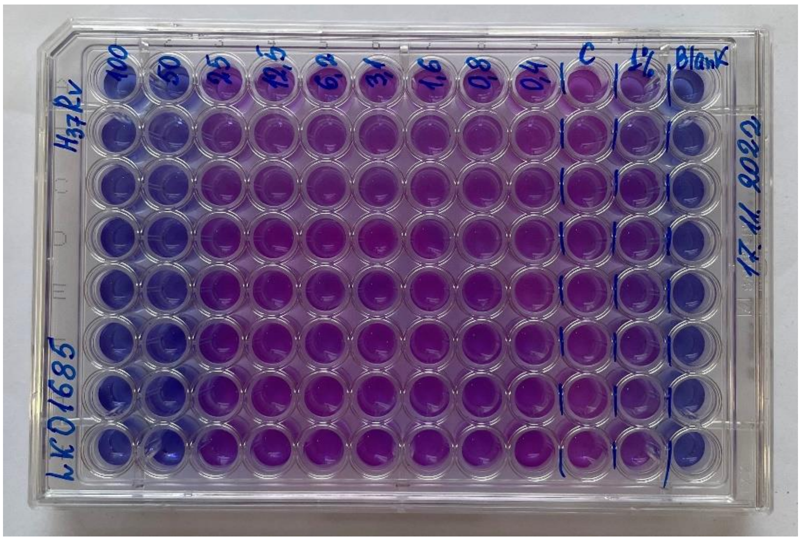

Compound **24** (MIC 6.2 μg/mL)

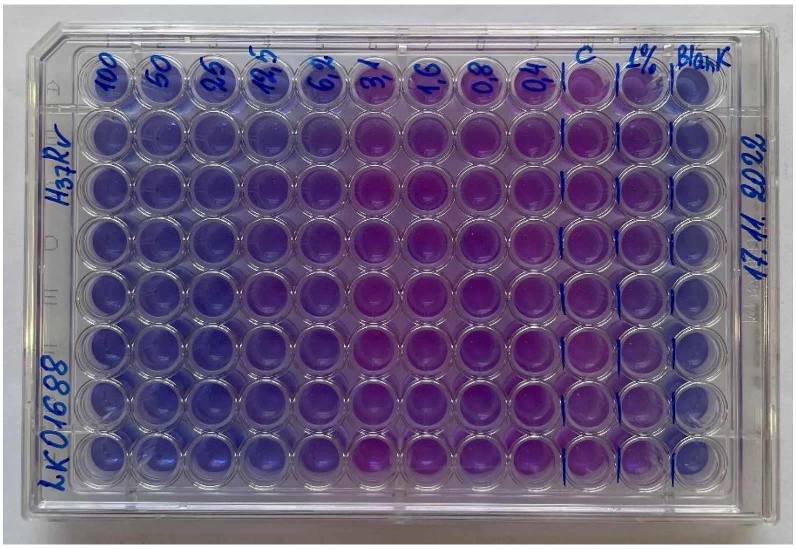

Compound **17** (MIC <0.4 μg/mL)

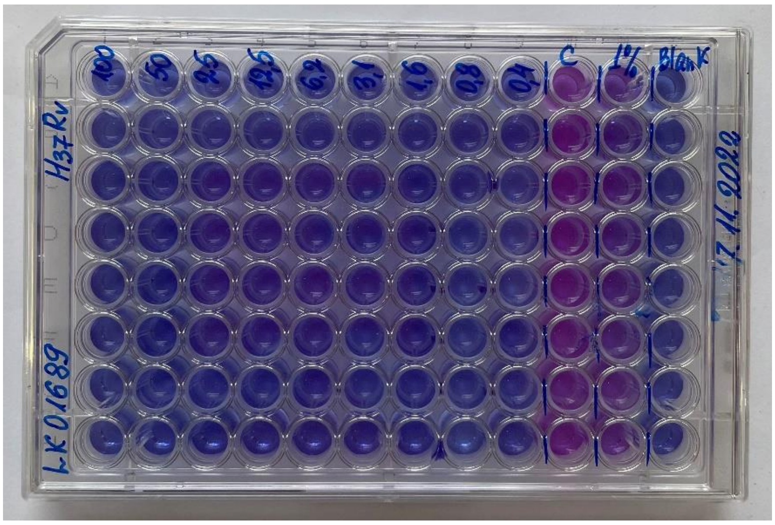

Supplement: Supplementary file 1 [file molecules-28-02529-s001.zip › molecules-2232855-supplementary-update.pdf]
